# Are digital health interventions acceptable and effective at improving medication adherence in paediatric patients with asthma? A mixed-mode systematic review, narrative synthesis, and behavioural analysis

**DOI:** 10.1080/21642850.2026.2745675

**Published:** 2026-11-11

**Authors:** Courtney Few, Selsebil Allani, Danica Cassar, Kate Hamilton-West, Clare Moloney, Jessica Walburn, Vivien Swanson

**Affiliations:** a IQVIA, Behavioural Science, Medical Communications, London, UK; b Division of Psychology, Faculty of Natural Sciences, University of Stirling, Stirling, UK; c Centre for Health Services Studies, University of Kent, Canterbury, Kent

**Keywords:** Paediatric asthma, digital health interventions, medication adherence, behaviour change theory, systematic review

## Abstract

**Background:**

Despite being important for asthma management, medication adherence remains suboptimal among paediatric patients. Digital Health Interventions (DHIs) offer a cost-effective and convenient approach to target the behavioural determinants of adherence and have been effective for other chronic conditions. This paper provides a comprehensive review and behavioural analysis of DHIs designed to improve medication adherence in paediatric patients with asthma.

**Methods:**

A mixed-mode systematic review (PROSPERO: CRD42022320097) was conducted by two independent reviewers. Seven databases were searched up to March 2025 for studies evaluating DHIs designed for paediatric patients with asthma and/or their caregivers to improve medication adherence (primary outcome). Secondary outcomes were patient quality of life (QoL), user-experiences, and DHI features or strategies and their associated theoretical constructs using the Theoretical Domains Framework (TDF). Quantitative and qualitative data were extracted and narratively synthesised.

**Results:**

Fifteen studies were included. All eleven studies that statistically assessed medication adherence reported significant results, in at least one context: either compared with controls (*N* = 5), baseline (*N* = 2), or within sub-groups (*N* = 4), but overall confidence was low. All three studies that qualitatively assessed adherence reported improvements, and with relatively high confidence. Three studies assessed QoL, but only one reported a significant improvement. Based on the user-experiences reported by ten studies, DHIs were well-accepted, with some usability concerns, and varying satisfaction and engagement. Financial incentives and pharmacist chat were well-praised and statistically effective features for improving medication adherence, commonly mapped onto Environmental context & resources, Reinforcement, Social influences, Intentions, and Behavioural Regulation.

**Conclusion:**

Due to study and review limitations, we conclude that multi-component DHIs are potentially acceptable and effective at improving medication adherence in paediatric patients with asthma. However, more high-quality theoretically informed longitudinal studies tailored to patient/caregiver unmet needs and barriers are needed to strengthen the evidence base on DHIs.

## Introduction

### Burden of asthma

Asthma is a chronic respiratory health condition characterised by wheezing, coughing, and shortness of breath; whereby exposure to allergens or stressors can result in (potentially fatal) asthma attacks (national health service, NHS, 2025). Since asthma is incurable, patients usually manage their condition using two types of prescribed inhalers: (i) preventer—administered daily to help reduce inflammation of the airways and prevent asthma symptoms (e.g. inhaled corticosteroids, ICS), and (ii) reliever—administered when symptoms become more severe (e.g. short-acting beta-agonists, SABA). Additionally, the management of asthma and related comorbidities often requires consideration of environmental factors, namely pet ownership, and exposure to tobacco smoke, air pollutants and pollen, since they are associated with increased risk of childhood asthma (Wang et al., 2025a) and worsening asthma outcomes (Annesi-Maesano et al., 2023; Docherty et al., 2025; Song et al., 2025).

Asthma is a common condition, with 300 million cases worldwide (Merhej & Zein, 2023). In the United Kingdom (UK), there are approximately 7.2 million people diagnosed with asthma (Asthma + Lung UK, 2024), and the prevalence is higher in paediatric (≤19 years) than adult populations (National Institute for Health and Care Excellence, 2013; Soriano et al., 2020). In England alone, approximately 25,000 children are hospitalised annually for asthma attacks (Mukherjee et al., 2022), and 54 paediatric asthma-related deaths were recorded between 2019 and 2023 (National Child Mortality Database, 2024). Additionally, paediatric asthma imposes a substantial clinical and economic burden in the UK, with mean annual NHS costs estimated at £ 1,086 per child and £ 297 per exacerbation (Kennedy et al., 2024). Despite individual differences concerning the onset, symptoms, trajectory, and related comorbidities of paediatric asthma, recent research suggests that there may be four distinct phenotypes: (1) almost controlled asthma, (2) early-onset asthma with atopic dermatitis and late-onset allergic rhinitis, (3) early-onset asthma only and (4) intermediate-onset asthma and late-onset allergic rhinitis (Yoon et al., 2024). These phenotypes highlight the diverse nature of asthma, and its potential utility in predicting clinical and economic outcomes and informing health treatments and services.

### Medication adherence

Medication adherence refers to the extent to which patients take their medications as prescribed by their healthcare providers (Specialist Pharmacy Service, 2023). Despite the availability of effective asthma medication in high income countries (Bissell et al., 2019), adherence remains a significant challenge among paediatric patients with asthma (Dahlén, 2022). For example, in the UK, it is approximated that only 36% of paediatric patients with asthma aged between 5 and 16 are adherent to their preventer inhaler (Paracha et al., 2023). Importantly, poor medication adherence is associated with suboptimal asthma control, defined as an inability to adequately manage symptoms or prevent exacerbations (De Keyser et al., 2020). In a UK primary care cohort, 41% of children aged 5 to 16 years were reported to have poorly controlled asthma (Lo et al., 2022). Poor medication adherence in paediatric patients with asthma is associated with poorer biological, psychological and social outcomes, including higher risk of asthma attacks, more hospital admissions, lower quality of life (QoL), and more absence from school (Andrenacci et al., 2022; Chongmelaxme et al., 2020; Engelkes et al., 2015; Lizano-Barrantes et al., 2023; Paracha et al., 2023; Serhal et al., 2020; Wong & Morton., 2021).

Medication adherence is a complex health behaviour that is influenced by inter-related factors, including barriers and facilitators with varying modifiability (Fan et al., 2021). For example, paediatric patients may not adhere to their asthma medication due to lack of access to medication, inaccurate health beliefs, forgetfulness, incorrect inhaler technique, and shame/embarrassment from medicine-taking in front of peers (De Keyser et al., 2020; Margolis et al., 2022; McCrossan et al., 2024; Rehman et al., 2020; Takkinsatian et al., 2022). Moreover, caregiver perceptions, such as concerns about medication safety and necessity (Rehman et al., 2020), and busy family lifestyles (McCrossan et al., 2024), can also adversely affect their child’s adherence to asthma medication. Exploring these inter-related factors helps researchers and healthcare professionals (HCPs) understand why some patients do not adhere to their asthma medication, and what type of intervention or service may be needed to support them. Since asthma management in paediatric patients typically involves multiple medications (i.e. preventer & reliever), and stakeholders (patients & caregivers), and is influenced by various biopsychosocial factors (e.g. distinct phenotypes, health beliefs, exposure to allergens), an effective intervention for improving medication adherence for this cohort would benefit from being theoretically informed (i.e. conceptualises the barriers), and multi-component (i.e. holistically addresses the barriers) (De Keyser et al., 2020; Rehman et al., 2020). For this review, a multi-component intervention is an intervention that targets multiple stakeholders, uses multiple devices or modalities (e.g. combination of digital and face-to-face elements), or multiple strategies to address various behavioural factors (Wang et al., 2025b).

### Behaviour change theories

Behavioural science approaches provide an awareness of how behaviours are changed and sustained. Although a variety of behavioural change theories have been used to understand medication adherence and inform intervention design (e.g. Pontinha et al., 2025), this review utilised the capability, opportunity, and motivation model of behaviour (COM-B) model (Michie et al., 2011) and the theoretical domains framework (TDF) (Atkins et al., 2017) due to their strengths in being accessible, systematic, practical, well-validated, and widely applicable (Atkins, 2017; Brown et al., 2024; Cowdell & Dyson, 2019; Whittal et al., 2021). As with any other behaviour change theory, the COM-B and TDF are not without limitations or criticism, but these do not undermine the utility and usefulness of such frameworks, since they offer a coherent and systematic approach to categorise behavioural determinants (e.g. Ofori et al., 2024) that can then map onto specific behaviour change techniques (BCTs) to develop effective health interventions (e.g. Buchanan et al., 2021; Eaton et al., 2024). According to the COM-B model, behaviour is determined by three general interacting components: the patients’ capability (physical & psychological), motivation (automatic & reflective), and opportunity factors (social & physical). The TDF extends this understanding by synthesising behavioural theories and theoretical constructs to identify 14 specific domains (e.g. knowledge, reinforcement, and social influences) that influence behaviour.

### Digital health interventions

Digital health interventions (DHIs) are an increasingly popular type of intervention delivered through technology to promote positive health behaviours and support condition management (Armitage et al., 2020). DHI modalities include smartphones apps, text-messaging, telephone or video calls, websites, patient portals, wearable technologies, and other digital devices or services (Murphy, 2021; Murray et al., 2016; Willis et al., 2022). Benefits include improving accessibility to medical care in patients (especially if socially isolated), improving efficiency and workloads in HCPs, and reducing financial burden on the patient and healthcare systems (Brewer et al., 2020; Gentili et al., 2022; Murphy et al., 2020; Willis et al., 2022).

DHIs have also been applied and proven to be effective in improving medication adherence across a variety of health conditions. Reviews suggest app-based interventions can increase medication adherence in patients living with cardiovascular disease, psoriasis, mental health conditions, Parkinson's disease, and those with multiple conditions (Armitage et al., 2020). More specifically, evidence demonstrates the positive impact of DHIs on medication adherence in paediatric populations, including text messaging for adolescents with sickle cell disease (Badawy et al., 2018) and liver transplants (Sayegh et al., 2022). Furthermore, DHIs have been shown to improve control (Farzandipour et al., 2024), QoL (Gümüş et al., 2024), and knowledge (Al Raimi et al., 2022) in paediatric patients with asthma, as well as lowering hospital admissions (Gümüş et al., 2024). Therefore, DHIs have the potential to be effective at improving medication adherence and other outcomes in patients with chronic health conditions, including asthma and paediatric populations.

Previous systematic reviews have demonstrated the benefit of DHIs on medication adherence in asthma but have limitations, including being arguably outdated (e.g. Morrison et al., 2014), largely dominated by studies conducted in white, western, high-income countries (van der Kamp et al., 2023), or reductionist by focusing on only one type of DHI or study design. For example, Ologundudu et al. (2025) exclusively reviewed studies that investigated smart inhalers. Although a narrow intervention scope provides the homogeneity desirable for meta-analysis, it limits comparisons across intervention types and may overlook effective multi-component interventions that integrate multiple digital devices or services (Wiecek et al., 2020). Moreover, Osama et al. (2024)’s review was restricted to quantitative studies, and in Ramsey et al. (2020)’s review, only randomised controlled trials (RCTs) were eligible for inclusion. Despite RCTs offering stronger quantitative evidence, systematic reviews that aim to inform future intervention development and decision-making are recommended to provide a comprehensive overview of quantitative, qualitative, and mixed-methods studies (Stern et al., 2021). These reviews demonstrate the promise of DHIs for asthma but highlight the need for an updated and comprehensive review accounting for multiple modalities for DHIs and study designs, and with a need to focus on the paediatric asthma population since they have a higher incidence rate and face unique additional challenges, such as the involvement of multiple stakeholders (i.e. patient and caregiver).

The aim of the current systematic review was to provide an up-to-date and comprehensive synthesis of quantitative, qualitative, and mixed-methods studies investigating the effectiveness of all types of DHIs for patients or their caregivers to improve medication adherence in paediatric patients with asthma. Additionally, we explored the impact of DHIs on QoL and user-experiences to capture patient and caregiver perspectives on usability, acceptability, engagement, and satisfaction. Finally, we conducted a behavioural analysis by mapping the DHIs onto the COM-B components and TDF domains to systematically identify the theoretical constructs associated with the most well-praised and effective digital features or behavioural strategies for behaviour change to inform future DHI design.

### Review questions

#### Primary

1. Are digital health interventions, targeting paediatric patients with asthma or their caregivers, effective at improving adherence to asthma medication?

#### Secondary

1. Are digital health interventions, targeting paediatric patients with asthma or their caregivers, effective at improving quality of life in paediatric patients with asthma?
2. What are the user-experiences (usability, acceptability, engagement, and satisfaction) of paediatric patients with asthma or their caregivers when using digital health interventions?
3. What digital features or behavioural strategies were well-praised and effective for behaviour change (i.e. improving medication adherence) and what are their associated theoretical constructs?

## Methods

### Protocol and registration

The systematic review followed the PROSPERO protocol, registered in May 2022—CRD42022320097. Since protocol registration, and due to the underestimated size of available literature, the scope of the initial multi-condition review was revised to focus exclusively on paediatric patients with asthma. Information was reported following two guidelines: (1) preferred reporting items for systematic reviews and meta-analyses (PRISMA) (Page et al., 2021) (see supplemental materials 1 & 2) and (2) synthesis without meta-analyses (SWiM) (Campbell et al., 2020) (see supplemental material 3). This project was completed without funding and for a professional health psychology doctorate placement. Therefore, there were limitations in scope and capabilities regarding eligibility, synthesis and theoretical mapping, including the lack of capacity for translation, backward and forward reference searching, meta-analysis, and contacting authors for identification of BCTs or for any other information.

### Eligibility criteria

Randomised and non-randomised intervention studies (i.e. qualitative, quantitative, and mixed-method study designs) published in English as journal articles were included. Eligible studies must have delivered a DHI through a digital device (e.g. computer, smartphone or wearable) or service (e.g. messaging or telephone calls) (Murphy., 2021) aiming to improve medication adherence, as a primary outcome, by targeting paediatric patients with asthma (≤19 years) and/or their caregivers. To be included, quantitative studies must have assessed medication adherence between-groups (i.e. intervention versus control group) and/or within-groups (i.e. baseline versus follow-up or post-intervention); and qualitative studies must have descriptively reported the impact of the DHI on medication adherence. Multi-component DHIs that incorporated a minimal face-to-face (F2F) component alongside digital delivery were included; interventions with substantial F2F delivery were excluded. Studies that did not assess medication adherence or delivered a DHI with the primary aim of improving another outcome (e.g. asthma knowledge) were excluded. Studies that recruited patients with asthma who were older than 19 years old or had a mixed sample of paediatric and adult patients were excluded. Journal articles published in languages other than English and review papers were excluded.

### Information sources

Seven databases were searched - PsycINFO, MEDLINE, PubMed, CINAHL, Scopus, Web of Science, and Cochrane Library - in May 2022 and updated in March 2025.

### Search strategy

The search strategy was developed and adapted to each database using free-text terms and controlled vocabulary, including words relating to children, asthma, DHIs, and medication adherence (see supplemental material 4). The search strategy was amended and re-run to account for the change of scope, focusing exclusively on paediatric patients with asthma. The PRISMA flowchart was updated to reflect this change (see Figure 1).

**Figure 1.**
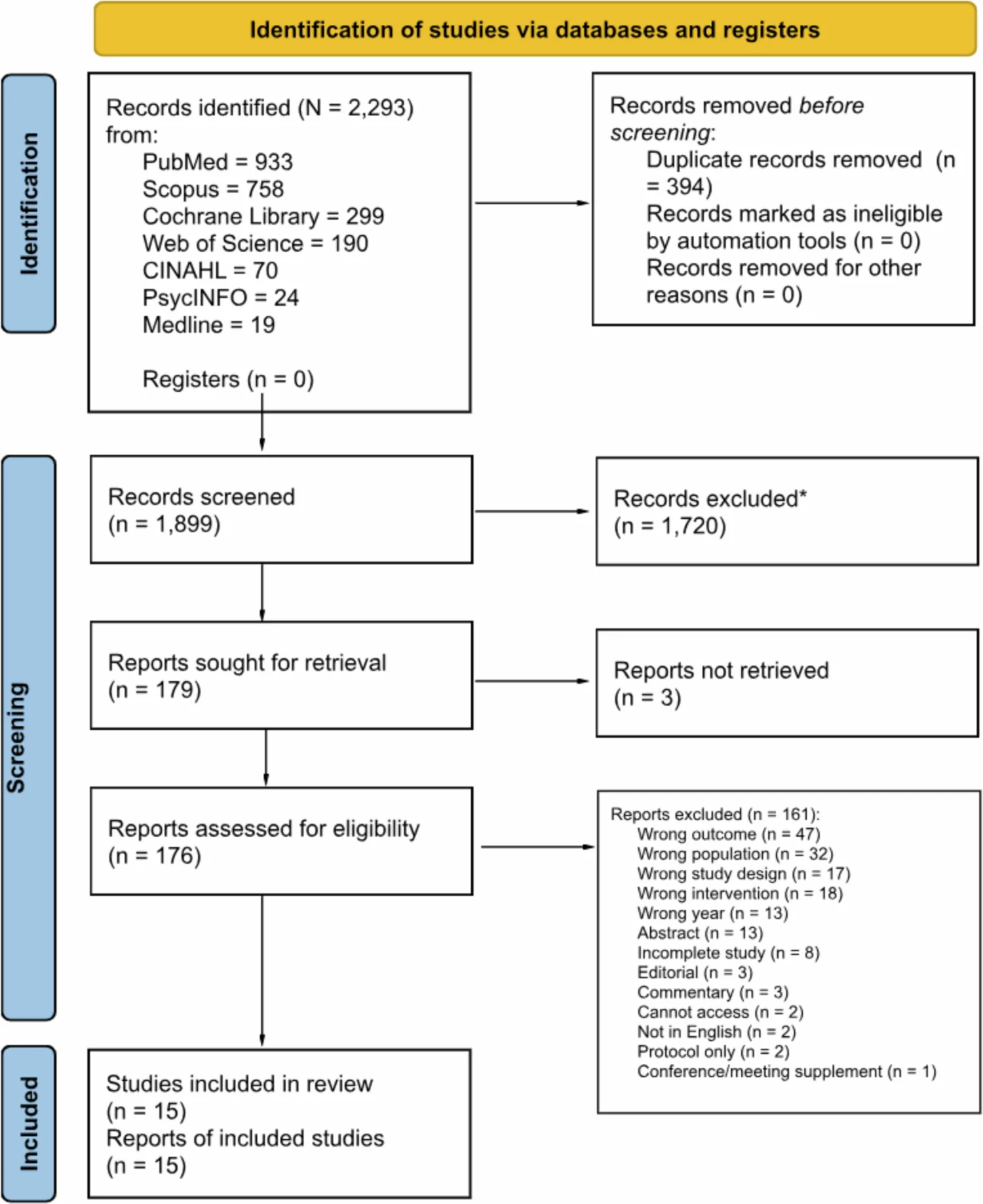
PRISMA flowchart of study identification and screening process. Note. The template was sourced from Page et al. (2021). Symbols & abbreviations: = , equals to, *n* = number, PRISMA = Preferred Reporting Items for Systematic Reviews and Meta-analyses.

### Selection process

After removing duplicates using Endnote (version 20 & 21, Clarivate, 2020; Clarivate, 2023), all titles and abstracts were imported into Rayyan QCRI software (version 2022, Ouzzani et al., 2016) and full texts were obtained. Two reviewers independently screened all records in the first (CF and DC) and updated search (CF and SA). Any discrepancies were resolved through discussion.

### Data collection process

Two authors (CF and SA) independently extracted data using Microsoft Excel (version 2304, Microsoft Corporation, 2023). Authors extracted: title, author, country, study design, participant characteristics, intervention characteristics, and results for primary (medication adherence) and secondary outcomes (patient QoL, user-experiences, digital features and strategies). Intervention characteristics were extracted in accordance with the template for intervention description and replication (TIDieR) checklist (Hoffmann et al., 2014). To adequately address the research questions, authors extracted all relevant descriptive (e.g. frequencies, percentages, and averages) and inferential values (e.g. t-values, chi-square, *p* values, standard deviation, Cohen’s d, odds ratio, and confidence intervals) from quantitative data. Effect sizes were extracted, if reported, and calculated using an online website: University of Colorado, Colorado Springs Effect Size Calculator (Becker, 2000), where possible and relevant. However, for qualitative data, the authors extracted all finalised reporting of qualitative analyses, including themes and quotes. All extracted data were cross-checked between the two reviewers to resolve discrepancies and ensure consistency. Where support was needed, the wider research team were consulted during team meetings.

Two reviewers (CF and SA) independently mapped the DHIs of the included studies onto the COM-B components and TDF domains, using the Atkins et al. (2017) paper for guidance. Two additional columns were added to the data extraction table for intervention characteristics to enable authors to perform theoretical mapping. After cross-checking between CF and SA, an additional reviewer (CM) provided expert oversight over the theoretical mapping.

### Assessment of study quality

Two reviewers (CF and SA) independently assessed the quality of included studies using the mixed methods appraisal tool (MMAT) (Hong et al., 2018), which is applicable across multiple study designs. It contains two screening questions with ‘Yes’ or ‘No’ responses, and five quality assessment criterion questions with the following responses: ‘Yes’ (i.e. criterion fully met), ‘No’ (i.e. criterion not met), ‘Somewhat’ (i.e. criterion partially met), or 'Can’t Tell’ (i.e. insufficient information to judge). Minor discrepancies were discussed and resolved between the two authors. No studies were excluded due to quality as per Hong et al. (2018)’s suggestion. Authors decided against using a summative score or a cut-off point to determine study quality since it is discouraged by Hong et al. (2018). Instead, study quality was synthesised qualitatively by highlighting key quality concerns (if any) per study design. Also, for added context, authors calculated the percentage of ‘Yes’ and ‘Somewhat’ responses per study and reported average percentages of these two responses per study design. Alongside MMAT, authors also considered effect size reporting, relative number of pilot or feasibility studies, and the consistency in the reporting of significant or positive findings when determining confidence in evidence per outcome or grouping.

### Synthesis methods

Findings were synthesised into a written narrative and using tabular and graphical formats (i.e. Table 1 and Figure 2). Results for medication adherence were grouped by data type, i.e. quantitative data (from quantitative and mixed-method studies) and qualitative data (from qualitative and mixed-method studies). Quantitative results for medication adherence and QoL were (further) categorised into (i) between-groups, and (ii) within-groups. A meta-analysis was not statistically appropriate or feasible due to substantial clinical and methodological heterogeneity across included studies. More specifically, studies (i) delivered various DHIs (i.e. devices, modalities, strategies/features), (ii) targeted various stakeholders (i.e. different target users, paediatric age groups, and service providers), and (iii) used various approaches to measuring and analysing medication adherence (i.e. different measures, sub-groups, timepoints and statistical tests). However, since studies were not suitable for meta-analysis, a harvest plot (Ogilvie et al., 2008) was designed to graphically represent and synthesise the quantitative statistical evidence for medication adherence, providing an overview of the variation in direction (significant vs non-significant), study quality (% of ‘Yes’ responses), measures (device-recorded vs self-reported), and groupings (within vs between groups in total and sub-sample populations).

**Figure 2.**
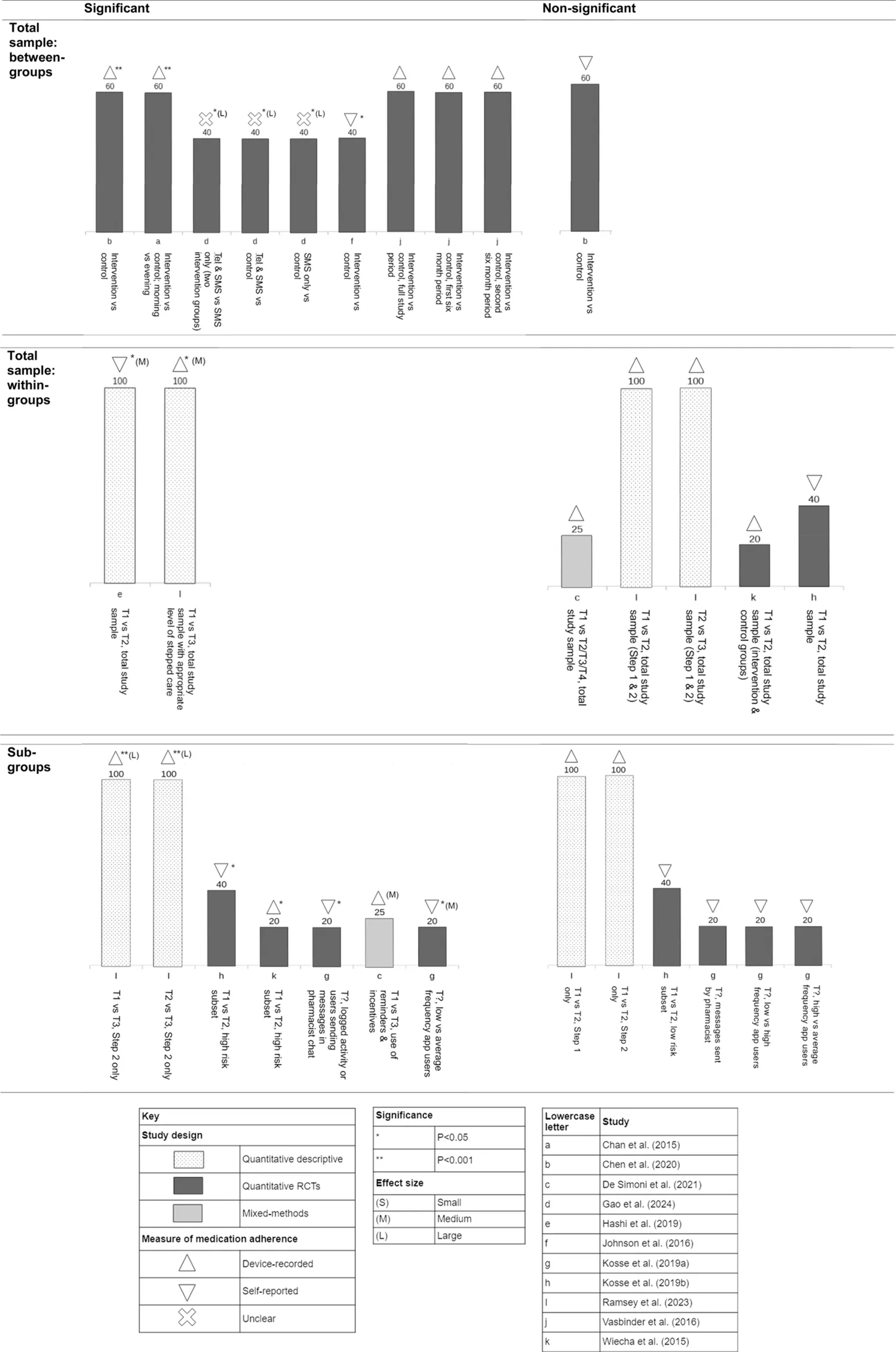
Harvest plots for the quantitative evidence on medication adherence. Note: Authors used the following two papers for guidance: Ogilvie et al. (2008) and Crick et al. (2015). The harvest plots were created manually using the LibreOffice suite (version 25.2.5.2, The Document Foundation, 2026). Results visualised under ‘significant’ represent significant improvements in medication adherence (positive direction), and results visualised under ‘non-significant’ represent non-significant results relating to medication adherence, regardless of direction. Significant decline in medication adherence was not visually represented in the harvest plots as it was only reported by one study for a particular sub-group (Step 1 only for T1 vs T3 and T2 vs T3, Ramsey et al., 2023). Each study is represented by a lower-case letter (in the x-axis) and a bar. Studies with multiple relevant findings, were given multiple bars to differentiate findings. To support interpretation, a caption is given below the x-axis. The study quality was represented by bar height (y-axis), specifically capturing the percentage of ‘Yes’ responses on the MMAT. The study design was represented by the bar colour. The measure used to assess medication adherence was represented by shapes above the bars. Finally, significance and effect sizes were represented by asterisks and capitalised letters, respectively. Symbols & abbreviations (beyond the key): RCTs = Randomised Controlled Trials, SMS = Short Message Service, Tel = Telephone, T = timepoint, vs = versus, ? = question mark (unclear), & = and.

Results for user-experiences were categorised into acceptability, usability, engagement, and satisfaction. While these dimensions are not drawn from a particular framework, they reflect common conceptualisations of user-experiences in digital health research. For example, usability and satisfaction are essential to the international organisation for standardisation (ISO) 9241-11 guidelines (Gunter et al., 2016; International Organization for Standardization, 2018), while acceptability and usability are key elements in the technology acceptance model (TAM) (Davis, 1989​​​; Kureshi et al., 2023). Also, the Perski & Short (2021) framework conceptualises acceptability into different facets, such as, burden, perceived effectiveness, and affective attitude; and Perski et al. (2020) provides a self-reported measure for engagement in digital behaviour interventions. In this review, acceptability concerned the perceived validity of the DHI in terms of purpose and modality (i.e. can DHIs be used for asthma management/medication adherence?), whereas usability concerned the perceived validity of the DHI in terms of digital literacy and functionality (i.e. do the DHIs actually work and do users understand how to use them?). Satisfaction concerned user perceptions around relevance, benefits and likeability of the DHI, and engagement concerned how frequently users used or plan to use the DHI.

Results for theoretical mapping were synthesised per: (i) digital feature or behavioural strategy, (ii) study, and (iii) theoretical construct (i.e. frequency count). The behavioural analysis was performed in extension to the theoretical mapping by highlighting the behavioural target (i.e. what barriers to medication adherence were addressed) and behavioural impact (i.e. what was the effect on medication adherence, and user-experiences) of each feature or strategy. The behavioural target was inferred by CF and SA from the full-text papers of included studies and the wider literature. The behavioural impact was collated from the synthesised results for each outcome of interest.

## Results

Across seven databases, 2293 papers were identified, and 176 were screened at full text, resulting in 15 eligible papers for inclusion.

### Study & outcome characteristics

Most studies were conducted in the United States of America (USA, *N* = 7) and the rest in the Netherlands (*N* = 3), China (*N* = 2), Japan (*N* = 1), New Zealand (*N* = 1), and the UK (*N* = 1) (see Table 1). Most included papers were RCTs (*N* = 8), the remaining studies were qualitative (*N* = 3), descriptive non-randomised quantitative (*N* = 2), and mixed methods (*N* = 2). Overall, 1292 patients and 42 patient-caregiver dyads were recruited, with the paediatric patient ages ranging from 1 to 17 years old. As classified by the NHS age range bandings (Age—NHS digital service manual, 2024), most participants were ‘teenagers’ (*N* = 7), and the rest were ‘child’ (*N* = 4), both ‘child’ and ‘teenager’ (*N* = 2), or ‘toddler’ (*N* = 2). Most studies assessed and/or monitored adherence to preventer inhaler (*N* = 9), and some to preventer and reliever inhalers (*N* = 6).

Studies had two timepoints (*N* = 6), three timepoints (*N* = 3), four timepoints (*N* = 2), six timepoints (*N* = 1), twelve timepoints (*N* = 1), or it was unclear (*N* = 2). All the studies assessed the primary outcome of medication adherence (*N* = 15). Most studies assessed medication adherence using digital tools (*N* = 10), where the assessment was either automated, i.e. recorded by the device itself (e.g. electronic monitoring devices, EMDs) (*N* = 6), or manually recorded on the device via self-report (e.g. apps and web-diaries) (*N* = 4). Questions used to assess self-reported medication adherence were sourced from the following validated questionnaires: medication adherence report scale (MARS, *N* = 2) (Thompson et al., 2000), adherence with asthma management for caregivers of preschoolers (AAMCP, *N* = 1) (Hashi et al., 2019), and asthma control test (ACT, *N* = 1) (Nathan et al., 2004).

The remaining studies assessed medication adherence using interviews (*N* = 4) - either semi-structured or focus groups, or the method of assessment was unclear (*N* = 1). Many studies conducted sensitivity and sub-groups analyses (*N* = 9) to assess the differentiated impact of: the measure of adherence (*N* = 1), level/step of care (*N* = 1), socio-demographic factors (i.e. adherence at baseline or timing of medication administration) (*N* = 3) or individual digital features (*N* = 4) on medication adherence. Several studies assessed the secondary outcome of QoL (*N* = 3), using a version of the following validated questionnaire: Paediatric Asthma Quality of Life Questionnaire (PAQLQ; Juniper et al., 1996; Juniper et al., 1999). Most studies assessed the secondary outcome of user-experiences (*N* = 10), either during intervention delivery (*N* = 6) or post-intervention (*N* = 4). User-experiences were assessed using interviews (*N* = 6), (online) surveys/questionnaires (*N* = 4), device data on engagement (*N* = 2), and/or study data on participation and retention (*N* = 1), or the method of assessment was unclear (*N* = 1). Some studies analysed socio-demographic differences in engagement (*N* = 2).

### Intervention characteristics

All DHIs aimed to improve adherence to (preventer) asthma medication and/or asthma management in paediatric patients with asthma. For some studies, the design process for the DHI involved theory (*N* = 3), e.g. eHealth theoretical models, or consultation with potential users and stakeholders (*N* = 2), or both (*N* = 3) (see appendix 1). Ramsey et al. (2023)’s DHI was adapted from a previous study and Nichols et al. (2020) modified their DHI during the iterative design & development phase. Five studies tailored their DHI, according to participants’ age (i.e. adolescents) (*N* = 1), timing for medication administration (*N* = 3), or personal barriers to medication adherence (*N* = 1). One study reported that their DHI was subject to fidelity checks and three studies tested the usability or quality of their digital devices, including the success of pairing devices or syncing data (e.g. EMD to app).

Most studies were conducted in a ‘real world’ setting, e.g. at home (*N* = 11), and others were conducted in a clinical setting, e.g. recruited and partly delivered at hospital (*N* = 4). DHIs targeted only patients (*N* = 9), both patients and their caregivers (*N* = 4), or only caregivers (*N* = 2). Studies used various electronic devices and services, most commonly smartphone apps (*N* = 10), EMD (*N* = 9), and web-based (*N* = 5). The most common DHI features and strategies were digital reminders (*N* = 14), digital user-monitoring (*N* = 14), access to data-driven digital services (e.g. personalised insights based on user-monitored data) or physical resources (e.g. medication lists) (*N* = 12), digital or F2F social support (*N* = 11), digital or F2F education (*N* = 10), and digital one-to-one professional support (*N* = 9) (see Table 2). Some studies had a control group (*N* = 9), which involved usual care with/without informational resources (*N* = 4), access to the device but without the active component (e.g. reminders & rewards) (*N* = 4), or it was unclear (*N* = 1).

Many DHIs were delivered by HCPs and/or researchers who provided generic or personalised medical, informational, emotional, or technical support (*N* = 11), including pharmacists, nurses, respiratory therapists, and trained psychology doctoral students. Although most studies delivered such support digitally (e.g. via chat features), three studies delivered HCP- or researcher-led education/training F2F. For most studies, participants received reminders or were instructed to monitor their outcomes, including medication adherence, daily (*N* = 6), twice daily (*N* = 3) or either (*N* = 1, i.e. adjustable to daily or twice daily by users). Otherwise, the frequency of reminders or user-monitoring were set weekly (*N* = 2), or it was unclear (*N* = 1). Interestingly, some studies provided (additional) alerts or reminders when medication doses/data entries were missing (*N* = 3) or in response to high-risk outcome values (*N* = 2).

**Table 1.** Summary of essential characteristics of included studies and their results on medication adherence.

Study & country	Design	Sample	Intervention	Medication adherence
***Mixed methods** (N = 2)*				
Cushing et al. (2016) USA	2-arm mixed methods feasibility study **Two** **timepoints:** T1 (baseline), T2 (12-week follow-up)	**Size:** 7 (*recruited)* 5 (*completed*) **Age in years (mean, range):** 14.1, 11-18 (NHS—Teenager)	**Intervention:** Multi-component intervention—Novel mhealth management system **Targeted user:** Patient **Devices:** (1) Smartphone app, and (2) EMD—sensor attached to inhaler **Digital features and behavioural strategies:** Reminders; customisation gamification; in-app rewards; user-monitoring; and access to data **Control:** Access to the digital tools (inhaler sensor & app) without reminders or rewards	**Medication:** Only preventer inhaler **Measure**: Self-reported adherence via interviews **Results**: An improvement from baseline to follow-up in the proportion of participants who forgot to take or skipped their preventer inhaler. Significance and effect size were not reported and could not be calculated. **T1:** All participants (7/7, 100%) reported sometimes forgetting to use their preventer inhalers and most participants (5/7, 74.43%) reported that when symptoms were managed, they did not use their preventer inhaler. **T2:** Most participants (4/5, 80%) reported that they occasionally forgot to take their preventer inhaler and some participants (2/5, 40%) reported that when symptoms were controlled, they did not take their preventer inhaler.
De Simoni et al. (2021) UK	Non-randomised mixed-methods feasibility study **Four timepoints:** T1 (baseline week 0), T2 (week 8), T3 (week 16), T4 (week 24)	**Size:** 10 (*recruited*) 8 (*completed*) **Age in years (mean, range):** 14.2, 12-16 (NHS—Teenager)	**Intervention:** Multi-component intervention—an electronic reminder and incentives intervention **Targeted user:** Patient **Devices:** (1) Smartphone app, (2) EMD—smart inhaler, and (3) web portal **Digital features and behavioural strategies:** Reminders; financial incentives; F2F skills training; education; user-monitoring; and access to data **Control:** N/A	**Medication:** Only preventer inhaler **Measure**: Device recorded adherence via EMD (Smart inhaler) **Results**: Significant improvement in preventer inhaler from baseline to follow-up (T3) among a sub-group of the study sample that was able to access and engage with the DHI, with a medium effect size (calculated). Throughout the study, there was no statistically significant change in adherence with preventer inhaler in the total study sample. There were observable positive trends from T1 to T2, and then a decline from T2 to T4. For the three participants able to pair the EMD with the app, receive reminders and self-monitor via the app throughout the study, adherence to preventer inhaler with reminders and incentives was higher at T3 0.86 (SD 0.05) compared to T1 0.51 (SD 0.07), with a calculated effect size of D = 5.75. Mean adherence: **T1:** 0.53 (SD = 0.20, *N* = 10) **T2:** 0.66 (SD = 0.18, *N* = 9) **T3:** 0.64 (SD = 0.28, *N* = 8) **T4:** 0.52 (SD = 0.24, *N* = 8) Calculated effect sizes (Cohen’s D): **T1 vs T2**: 0.68 (medium) **T1 vs T3:** 0.45 (small) **T1 vs T4:** -0.06 **T2 vs T3:** -0.08 **T2 vs T4:** -0.66 **T3 vs T4:** -0.46
***Quantitative RCTs** (N = 8)*				
Chan et al. (2015) New Zealand	2-arm RCT **Four timepoints:** T1 (baseline), T2 (2-month follow-up), T3 (4-month follow-up), T4 (6-month follow-up)	**Size:** 220 (*randomised*) 213 (*completed*) **Age in years (mean, SD):** Intervention: 8.9, 2.5 Control: 8.9, 2.6 (NHS—Child)	**Intervention:** Multi-component intervention - EMD with audiovisual reminder feature enabled **Targeted user:** Patient **Device:** (1) EMD—smart inhaler **Digital features and behavioural strategies:** Audiovisual reminders; user-monitoring; access to data; and caregiver involvement **Control:** Access to the digital tool (EMD) without audiovisual reminders	**Medication:** Preventer & reliever inhalers **Measure**: Device-recorded adherence via SmartTrack for preventer inhalers and Smart inhaler Tracker for reliever inhalers **Results:** Significant improvement in medication adherence in the intervention group, compared with controls. Effect size was not reported and could not be calculated. The median percentage of adherence was 84% in the intervention group (10th percentile 54%, 90th percentile 96%), compared with 30% in the control group (8%, 68%; *P*-value < 0.0001). A higher proportion of participants in the intervention group had > 70% adherence than participants in the control group. Across T2, 3, & 4, the control group had better adherence in the evening than in the morning, whereas the intervention group had better adherence in the morning than in the evening (*P*-value = 0.0003). Overall adherence declined in the intervention and control groups over time, with no difference in the rate between the two (*P*-value = 0.10). Mean percentage of adherence—Intervention: **T2:** 91% **T3:** 84% **T4:** 79% Mean percentage of adherence—Control: **T2:** 40% **T3:** 33% **T4:** 27%
Chen et al. (2020) China	Multicentre, single-blind, parallel-group clinical RCT **Six timepoints**: T1, T2, T3, T4, T5, T6 (monthly follow ups)	**Size:** 96 (*randomised*) 86 (*completed*) **Age in years (mean, SD):** Intervention: 2.15, 0.8* Control: 2.275, 1.02* *Converted from months to years (NHS—Toddler)	**Intervention:** Multi-component intervention—Electronic monitoring combined with IMS-based weekly feedback plus reminders **Targeted user:** Patient & caregiver **Devices:** (1) Smartphone app, and (2) EMD—sensor attached to inhaler **Digital features and behavioural strategies:** Reminders; user-monitoring; skills training; education and feedback from HCP; and caregiver involvement **Control:** Access to the digital device (EMD) without reminders or feedback through the IMS component	**Medication:** Only preventer inhaler **Measure**: Two measures - (i) device-recorded adherence via SmartTrack Device and (ii) self-reported adherence by caregiver via two questions **Results:** Significant improvement in device-recorded adherence to preventer inhaler in the intervention group, compared with controls. Effect size was not reported and could not be calculated. Device-recorded adherence: the mean adherence was better in the intervention group (80.0%), than the control group (45.9%), with a difference of 34.0% (95% CI, 26.8–41.3%; *P*-value < 0.001). The mean adherence difference (95% CI) at T3 and T6 for the intervention group, compared with the control group was 35.2% (17.3–53.1%) and 47.3% (29.7–64.9%), respectively. *Mean adherence—intervention:* T1: 92.6 (SD = 19.7) T2: 87.5 (SD = 28.1) T3: 79.9 (SD = 33.5) T4: 74.0 (SD = 41.0) T5: 73.4 (SD = 42.4) T6: 72.3 (SD = 41.5) *Mean adherence—control:* T1: 77.8 (SD = 28.7) T2: 58.9 (SD = 41.3) T3: 44.8 (SD = 44.9) T4: 36.1 (SD = 43.0) T5: 33.1 (SD = 44.9) T6: 25.0 (SD = 36.0) Self-reported adherence by caregivers: no significant differences were found in mean adherence rates between the intervention group (89.7%) and the control group (92.7%) (*P*-value = 0.452). *Mean adherence—intervention:* T1: 93.0 (SD = 20.3) T2: 85.3 (SD = 27.9) T3: 92.3 (SD = 20.2) T4: 91.1 (SD = 23.1) T5: 90.5 (SD = 23.1) T6: 86.4 (SD = 27.0) *Mean adherence—control:* T1: 95.3 (SD = 13.4) T2: 94.9 (SD = 14.1) T3: 96.3 (SD = 11.4) T4: 92.8 (SD = 18.4) T5: 87.6 (SD = 24.0) T6: 89.5 (SD = 21.5)
Gao et al. (2024) China	3-arm RCT **Two timepoints:** T1 (baseline at week 0), T2 (week 12)	**Size:** Total: 120 (*enrolled*) 95 (*completed*) *N* completed per group: Telephone & SMS group: 37 SMS group: 32 Control: 26 **Age in years (mean, SD):** 7.89, 3.01 (NHS—Child)	**Intervention:** Multi-component intervention **-** Telephone & SMS intervention **Targeted user:** Caregiver **Device:** (1) Smartphone - text & telephone **Digital features and behavioural strategies:** Reminders; F2F education; and caregiver involvement **Control:** Access to the F2F general guidance and verbal reminders for revisits without telephone or SMS intervention	**Medication:** Only preventer inhaler **Measure**: Unclear—compliance assessment **Results:** Significantly better adherence to preventer inhaler in both intervention groups (Tel + SMS; SMS only), compared with controls, with a large effect size. There was a statistically significant difference in adherence to preventer inhaler between the three groups (χ2 = 28.388, *P*-value < .05). Tel + SMS group: all of the participants in this group who completed T2 (86.0%) adhered to their preventer inhaler (37/37), which was significantly different from both the SMS only group and the control group (χ2 = 7.862, *P*-value < .01; χ2 = 28.497, *P*-value < .01). SMS only group: some of the participants in this group who completed T2 (56.0%) adhered to their preventer inhaler (18/32), which was significantly different from the control group (χ2 = 8.215, *P*-value < .01). Control group: only a minority of the participants in this group who completed T2 (19%) adhered to their preventer inhaler (5/26). Effect sizes - OR (95% CI): Tel + SMS vs SMS only: 5 (1.6—14.0) Tel + SMS vs control: 27 (6.7—93) SMS only vs control: 5.4 (1.7—16)
Johnson et al. (2016) USA	2-arm feasibility RCT **Three timepoints:** T1 (baseline at week 0), T2 (7-day period), T3 (follow-up at week 3)	**Size**: 98 (*randomised*) 89 (*completed*) **Age in years (mean, SD):** Intervention: 14.17, 1.83Control: 13.93, 1.54 (NHS—Teenager)	**Intervention:** Multi-component intervention—text-message based reminder system **Targeted user:** Patient **Devices:** (1) Smartphone - text, and (2) MMH web application (usable on a smartphone, desktop or tablet) **Digital features and behavioural strategies:** Time-tailored reminders; additional reminder for ‘held’ doses; access to structured medication lists; user-monitoring; access to data; onboarding instructions and video; financial compensation; caregiver involvement; and technical support/helpdesk **Control:** Access to the F2F general guidance and verbal reminders for revisits without telephone or SMS intervention	**Medication:** Preventer & reliever inhalers **Measure:** Self-reported adherence via web-app question about adherence to preventer inhaler **Results**: Significant improvement in adherence to preventer inhaler & QoL in the intervention group, compared with controls. Effect size was not reported and could not be calculated. Seven-day adherence to preventer inhaler significantly improved in the intervention group, compared with controls, with an average of 1 day gain of adherence, and a median change from 4 to 6 days in the former (*P*-value = 0.011) vs no median change in the latter. For the intervention group, these numbers were consistent with observed behaviours documented through MMH. Seven-day adherence remained unchanged in participants who did not receive reminders. Intervention: **T1**: 4.25 **T2**: 4.86 *Change*: 0.61 Control: **T1**: 5.17 **T2**: 3.83 *Change*: 1.35
Kosse et al. (2019a) Netherlands	Cluster RCT (*only data for the intervention group was reported*) **Timepoints—unclear** The trial lasted for 6 months with adherence questionnaires being sent out fortnightly 14 times	**Size**: Intervention: 103 (*consented*) 87 (*analysed*) 27 pharmacists were recruited. **Age in years (mean, SD):** 15.0, 2.0 (NHS—Teenager)	**Intervention:** Multi-component intervention - ADAPT **Targeted user:** Patient **Devices:** (1) Smartphone app, and (2) desktop app for HCPs **Digital features and behavioural strategies:** Reminders (alarms & push notifications); in-app educational short movies; user-monitoring; access to data; peer chat; goal setting; and feedback, education, and support from HCPs **Control:** Access to usual care and online educational materials without additional medication management support	**Medication:** Only preventer inhaler **Measure**: Self-reported adherence via two in-app MARS inspired questions **Results**: Significant improvement in adherence to preventer inhaler among a sub-group of the intervention group that initiated and engaged with the pharmacist chat feature. Effect size was not reported and could not be calculated for this finding. Total app use was not associated with a difference in medication adherence (*P*-value = 0.12). Use of the CARAT questionnaire (*P*-value = 0.26), adherence questions (*P*-value = 0.65), short movies (*P*-value = 0.80), or peer chat (*P*-value = 0.21) also did not affect the adherence outcome. Adherence according to engagement: Logged activity in pharmacist chat significantly improved medication adherence (MARS score increased with 0.1 points per message; *P*-value = 0.03). Medication adherence was positively impacted when participants sent messages to pharmacists (*P*-value = 0.01, MARS score increased with 0.3 points per chat message), but not when pharmacists send messages to participants (*P*-value = 0.06). The low-frequency app users had lower rates of medication adherence compared with the average group (MARS 19.3 vs 21.4; *P*-value = 0.04), with a medium effect size (calculated). Adherence scores - mean (SD): Total: 20.4 (3.0) Low-frequency app users (≤10 times): 19.2 (3.9) Average-frequency app users (>10 and ≤ 25 times): 21.4 (3.5) High-frequency app users (>25 times): 20.3 (4.2) Adherence scores—*P*-values and calculated effect sizes: Low vs average: *P*-value = 0.04, D = 0.59 (medium) Low vs high: *P*-value = 0.30, D = 0.27 (small) High vs average: *P*-value = 0.35, D = -0.28 (small)
Kosse et al. (2019b) Netherlands	Cluster 2-arm RCT **Two Timepoints:** T1 (baseline at month 0), T2 (follow-up at month 6)	**Size**: Total: 253 (*consented*) 234 (*completed*) Intervention: 103 (*consented*) 87 (*completed*) Control: 150 (*consented*) 147 (*completed)* **Age in years (mean, SD):** Total: 15.1,1.9 Intervention: 15.0, 2.0 Control: 15.2, 1.9 (NHS—Teenager)	**Intervention:** Multi-component intervention - ADAPT **Targeted user:** Patient **Devices:** (1) Smartphone app, and (2) desktop app for providers **Digital features and behavioural strategies:** Reminders/alarms; user-monitoring; access to data; educational short movies; peer chat; goal setting; and feedback, education, and support from HCPs **Control**: Unclear	**Medication:** Preventer & reliever inhalers **Measure**: Self-reported adherence via in-app MARS questions **Results**: Significant improvement in adherence to preventer inhaler among a sub-group of the intervention group that had low baseline adherence. Effect size was not reported and could not be calculated for this finding. No significant effect of the intervention on the MARS score for the total study population (*P*-value = 0.25, OR = + 0.60, CI: -0.43, 1.63). However, there was an interaction between MARS baseline score and the intervention (*P*-value = 0.02). Sensitivity analysis showed that adherence rates of patients with low baseline adherence (*N* = 76; MARS ≤ 19) increased by 1.42 points in the intervention group (*N* = 26), whereas it decreased by 0.70 points in the control group (*N* = 50). There was a significant intervention effect (MARS + 2.12; *P*-value = 0.04) in poorly adherent participants. This intervention effect was stronger (MARS + 2.52, *P*-value = 0.02) in poorly adherent participants with uncontrolled asthma (*N* = 74; MARS ≤ 20 and CARAT ≤ 24). No significant effect of the intervention in improving medication adherence when using the MARS cut-off score (≥ 23) as an indicator, OR 1.07 [CI 0.54; 2.20]. Sensitivity analysis (varying from MARS ≥ 19 to ≥ 24) also showed no effect of the intervention on medication adherence. At baseline, 33.3% (*N* = 29) of the intervention group and 40.1% (*N* = 59) of the control group were adherent to medication based on the MARS cut-off (score ≥ 23). This percentage increased in the intervention group to 36.8% (*N* = 32) and decreased in the control group to 38.8% (*N* = 57). Intervention (total): **T1:** 20.4 (3.9) **T2**: 19.9 (4.0) Control (total): **T1**: 20.4 (4.0) **T2**: 19.3 (5.1) Intervention effect: *P*-value: 0.25, effect size: + 0.60 (−0.43; 1.63)
Vasbinder et al. (2016) Netherlands	Multicentre 2-arm RCT **Twelve timepoints:** T1 to T12 (monthly follow-up)	**Size**: Total: 219 (*randomised*) 209 (*analysed*) Intervention: 108 (*randomised*) 101 (*analysed*) Control: 111 (*randomised*) 108 (*analysed*) **Age in years (mean, SD):** Intervention: 7.8, 2.2 Control: 7.7, 2.1 (NHS—Child)	**Intervention:** Multi-component intervention—RTMM SMS **Targeted user:** Patient & caregiver **Devices:** (1) Smartphone—text, and (2) EMD—sensor attached to inhaler **Digital features and behavioural strategies:** Time-tailored reminders; additional reminder for missed/unrecorded medication; user-monitoring; and caregiver involvement **Control:** Access to usual care (initial inhalation instruction & automated pharmacy information systems)	**Medication:** Only preventer inhaler **Measure**: Device-recorded adherence via RTMM **Results**: Significant improvement in adherence to preventer inhaler in the intervention group, compared with controls. Effect size was not reported and could not be calculated. Overall adherence: Over the full study period, adherence in the intervention group was 69.3% (95% CI 65.5–73.4%) and 57.3% (95% CI 52.8–61.7%) in the control group. The overall difference (12.0%) was statistically significant (95% CI 6.7–17.7%). The average estimated treatment effect over T1 to T6 (15.0%, 95% CI 9.3–20.7%) was larger than in T7 to T12 (9.0%, 95% CI 2.4–16.3%), but both were statistically significant. Effect of SMS reminders on adherence: SMS reminders were sent 56.8% of preventer inhaler doses in the intervention group, and approximately half of these reminders (53.3%) led to timely administration. Especially during the first months of the study, participants in the intervention group were substantially more adherent than participants in the control group. In both groups, adherence decreased steadily during the first 6 months, after which it remained stable and statistically significant for most measurement times.
Wiecha et al. (2015) USA	Prospective pilot 2-arm RCT **Two timepoints:** T1 (two baseline home visits that are two weeks apart), T2 (6-month follow-up)	**Size**: Total: 58 (*randomised*) 42 (*analysed*) Intervention: 37 (*randomised*) 28 (*analysed*) Control: 21 (*randomised*) 14 (*analysed*) **Age in years (mean, SD, range):** Intervention: 11.9, 2.0, 8-16 Control: 12.9, 3.0, 7-17 (NHS—Child & Teenager)	**Intervention:** Multi-component intervention—BB **Targeted user:** Patient & caregiver **Devices:** (1) EMD—sensor attached to inhaler, and (2) web-based platform for users & providers **Digital features and behavioural strategies:** Alerts of dangerous values; feedback and support from asthma specialists; discussion board with peers and HCPs; user-monitoring; access to data; education and quizzes; skills training; in-app rewards; financial incentives; and caregiver involvement **Control:** Access to usual care, an asthma education manual, and peak flow meter	**Medication:** Only preventer inhaler **Measure**: Device-recorded adherence via DOSER **Results**: Significant improvement in adherence to preventer inhaler among a sub-group of the intervention group that had low baseline adherence. Effect size was not reported and could not be calculated. No significant improvement in medication adherence in the total intervention and control groups when compared to baseline. However, adherence to preventer inhaler significantly improved from T1 to T2 among the subgroup of the intervention with low baseline adherence (< 75%). Adherence scores: Intervention - Total: **T1** mean: 38.0, mean change at **T2**: + 11.2, *P*-value: 0.30 Control - Total: **T1** mean: 45.9, mean change at **T2**: -4.4, *P*-value: 0.81 Intervention - High-risk participants: **T1** mean: 16.3, mean change at **T2**: + 29.8, *P*-value: 0.01 Control - High risk participants: **T1** mean: 39.2, mean change at **T2**: - 5.0, *P*-value: 0.81 *P*-value for differences in changes: Total: 0.46 High-risk participants: 0.10
***Quantitative descriptive** (N = 2)*				
Hashi et al. (2019) Japan	Cohort pre-post interventional study (descriptive quantitative) **Two timepoints:** T1 (baseline at week 0), T2 (study completion at week 4)	**Size:** 58 (*recruited*) 45 (*completed*) **Age in years (mean, SD):** 3.47, 1.42 (NHS—Toddler)	**Intervention:** Multi-component intervention - Caregiver web-based diaries intervention **Targeted user**: Caregiver **Devices:** (1) Web-based diaries, and (2) email **Digital features and behavioural strategies:** Reminders; support and feedback from researchers; user-monitoring; and caregiver involvement **Control:** N/A	**Medication:** Only preventer inhaler **Measure**: Self-reported adherence via AAMCP inspired web-based diary entries **Results**: Significant improvement in adherence to preventer inhaler post-intervention, compared to baseline, with a medium effect size (calculated). The average AAMCP score was 49.78 ± 7.20 at T1 and increased to 52.13 ± 6.25 at T2, and this was a statistically significant difference (T-value = −3.07, *P*-value = 0.004, 95% CI [−3.90, −0.81]), with a calculated effect size of D = 0.35. Most participants (53.3%, 24/45) administered the preventer inhaler as prescribed (i.e. daily). However, some participants (46.6%, 21/45) could not administer the preventer inhaler as prescribed (i.e. only half of the doses were given or not at all).
Ramsey et al. (2023) USA	Pilot baseline intervention (descriptive quantitative) **Three timepoints:** T1 (baseline, 4-week duration) T2 (Step 1, 4-8 weeks duration) T3 (Step 2, 5-week duration until study end)	**Size**: 30 (*enrolled*) 26 (*completed*) **Age in years (mean, SD):** 14.70, 1.57 (NHS—Teenager)	**Intervention:** Multi-component intervention—Technology-Assisted Stepped-Care Inter2020vention *Step 1:* App-based digital medication reminders and adherence monitoring *Step 2:* Telehealth behavioural intervention and app-based adherence feedback **Targeted user:** Patient **Devices:** (1) Smartphone app, and (2) telehealth behavioural sessions **Digital features and behavioural strategies:** Reminders; user-monitoring; access to data; F2F skills training by HCP; goal setting; feedback, education, and support from researcher via telehealth sessions; modification; and tailoring **Control:** N/A	**Medication:** Only preventer inhaler **Measure**: Device-recorded adherence via EMD **Results**: Significant improvement in adherence to preventer inhaler post-intervention, compared to baseline, when participants received appropriate stepped care, with a medium effect size. No significant improvement in adherence for the full sample from T1 to T2 (T-value = −.47, *P*-value = .641, D = .25). However, adherence significantly improved from T1 (40%) to T3 (58%) when participants received the appropriate intervention according to the stepped-care model, with a medium effect size (T-value = −2.14, *P*-value = .048, D = .52). For the subsample that received only Step 1, adherence declined from T1 (69%) to the T3 (46%) (T-value = 4.26, *P*-value = .013, D = 1.90). Although adherence initially increased to 73% during the first four weeks of Step 1, the effect size was small (T-value = .04, *P*-value = .432, D = .35). For participants who progressed onto Step 2, adherence remained relatively stable from T1 (30%) to T2 (33%; T-value = −.22, *P*-value = .826, D = .07), highlighting the need for a more intensive Step 2 intervention. In this subset, adherence significantly improved from T1 (30%) to T3 (65%, T-value = −5.63, *P*-value < .001, D = 1.70), with a large effect size. **Mean difference in adherence scores, M (SD), *P* Value:** Full sample **T1 to T3:** .19 (.37), .048 **T1 to T2:** .02 (.20), .641 **T2 to T3:** .11 (.36), .165 Only received step 1 **T1 to T3**: .27 (.14), .013 **T1 to T2**: .04 (.12), .432 **T2 to T3:** 30 (.21), .009 Received step 2 **T1 to T3:** 40 (.24), < .001 **T1 to T2:** .02 (.24), .826 **T2 to T3:** .31 (.25), < .001
***Qualitative** (N = 3)*				
Kan et al. (2021) USA	Qualitative study **Timepoints—unclear** Interviews were conducted between March and July in 2019, one interview per participant	**Size:** 20 caregiver-child dyads (*interviewed*) **Age in years (mean, SE):** 8.7, 0.6 (NHS—Child)	**Intervention:** Multi-component health intervention—EMM-based iTRACC intervention **Targeted user:** Patient & caregiver **Devices:** (1) Smartphone app, (2) EMD—sensor attached to inhaler, (3) web portal (for HCPs), (4) email (for HCPs) **Digital features and behavioural strategies:** Time-tailored reminders; push notifications for missed medication; user-monitoring; access to data and reports; alerts notifying HCPs of concerning changes (i.e. increase in reliever inhaler usage or decrease in preventer inhaler usage); feedback from HCP; and caregiver involvement **Control:** N/A (only the intervention arm was interviewed in this study)	**Medication:** Preventer & reliever inhalers **Measure**: Self-reported adherence via interviews **Results**: Did not assess significance, however participants descriptively reported improvements in adherence to preventer & reliever inhalers because of the DHI. Participants reported that different digital features supported them to adhere to their preventer & reliever inhalers, and asthma management overall—a key theme. For adherence to preventer inhalers, participants reported improvement with app reminders, using the DHI to establish a routine or schedule that was mostly maintained after study end, using the pollen warnings to prepare for triggers, and having an increased awareness overall of their child’s asthma-related needs. Participants who had already established reliable asthma management routines pre-intervention reported appreciating the technology but admitted that it did not change their behaviours. For adherence to reliever inhalers, participants felt that one of the most useful features was the ability to monitor reliever inhaler use during asthma exacerbations. Participants reported that reviewing their child’s reliever inhaler use aided them in identifying triggers or patterns of asthma exacerbations. For example, a parent would not send their child outside to play on high-trigger days because of pollen or weather changes. They also reported that the app replaced pen and paper or other previous methods in tracking reliever inhaler use. Participants described pulling up the app record for the doctor at clinic visits, enabling them to provide an accurate account to the doctor and preventing them from having to rely on their memory, which was less accurate.
Nichols et al. (2020) USA	Qualitative pilot (*but includes one table with quantitative results*) **Three timepoints:** T1 (Phase 1 - feasibility & acceptability assessment), T2 (Phase 2 - intervention delivery, enrolment), T3 (Phase 2—intervention delivery, 2-month visit)	**Size**: 22 caregiver-child dyads (*completed study intervention*) 19 caregiver-child dyads (*participated in post-intervention interviews*) **Age in years (mean, SD):** Total: 10.89, 2.73 Phase 1 (*N* = 14): 8-10 = 9, 64 11-13 = 3, 21 14-16 = 2, 14 Phase 2 (*N* = 8): 8-10 = 4, 50 11-13 = 2, 25 14-16 = 2, 25 (NHS—Child & Teenager)	**Intervention:** Muti-component intervention—the SAMS app **Targeted user:** Patient **Devices**: (1) Smartphone—app & text, (2) EMD—cap attached to inhaler, and (3) telehealth sessions **Digital features and behavioural strategies:** Reminders; user-monitoring; access to data; skills training; feedback, support and motivational interviewing from HCP; caregiver involvement; and modification **Control:** N/A	**Medication:** Preventer & reliever inhalers **Measure**: Self-reported adherence via interviews (Phase 1 & 2) and surveys (Phase 2) **Results:** Did not assess significance, however participants descriptively reported improvements in adherence to preventer inhaler because of the DHI. Both phases: Some participants describe the benefit of capturing inhaler use on improving inhaler technique and the ability to correctly adhere to asthma medication. The benefit of reminders on medication adherence was a recurring theme throughout interviews. The use of technology to increase medication adherence and accuracy of medication administration was supported by both children and parents. Although, intentions were strong to follow medical advice, the realities of balancing demanding schedules and daily responsibilities pose challenges to actual adherence. Phase 2: **T1**: 3.8 (0.7) **T2**: 4.2 (0.7) *P*-value: 0.30 Patient quotes: “*It helps me. Like it reminds me that I need, in case I forget to take my medicine or anything, it reminds me to stop whatever I’m doing and go take my medicine*.” [Child #1, phase 2] "*I think that’s good because you can learn how to take your medicine better so that it’ll work better.*" [Child #8, phase 1]
Schneider et al. (2020) USA	Qualitative, exploratory study **Two timepoints:** T1 (mid-point interview), T2 (follow-up interview at study completion)	**Size**: 20 (*recruited & participated*) **Age in years (mean, SD, range):** 14.4, 1.6, 12-17 (NHS—Teenager)	**Intervention:** Multi-component intervention - Asthma self-management mobile app **Targeted user:** Patient **Devices:** (1) Smartphone app, and (2) EMD—peak flow meter **Digital features and behavioural strategies:** Reminders; user-monitoring; access to data (visual graphs); goal tracking; skills training; customisation; feedback and support from HCP; and tailoring **Control:** N/A	**Medication:** Preventer & reliever inhalers **Measure**: Self-reported adherence via interviews **Results**: Did not assess significance, however participants descriptively reported improvements in adherence to preventer inhaler because of the DHI. Most participants (*N* = 17) participants reported that the app [prompted them to administer their preventer inhaler regularly and on-time, resulting in more consistent adherence. Some participants (*N* = 8) appreciated the benefit of getting specific instructions for reliever inhaler use when their asthma was in the yellow or red zone. App use also reminded two users to take their inhaler with them to school or other out-of-home activities. Several participants thought the improved medication adherence resulted in an overall better asthma control and fewer exacerbations. Patient quotes: "*It was good, because it helped me remember to take my medicine every day (even though I already do that). Some days I missed it because I was too busy*.” “*Sometimes I forget to take my medicine, and it sends me a text, and I’m like, ‘Oh yeah, I need to take my medicine.’ Because sometimes I will forget.*”

Note: Studies were grouped by study design and ordered in alphabetical order of first author's surname within each grouping. Columns were inspired by the PICOS framework - Population, Intervention, Comparison, Outcomes and Study design (Amir-Behghadami & Janati, 2020).

NHS bandings: toddler = 1 to 3 years, child = 4 to 12 years, teenager = 13 to 19 years, young people = 16 to 24 years.

For quantitative results/studies, the key significant finding was highlighted and reported first, followed by more detailed results. For qualitative results, the key finding was summarised, followed by more detailed results and patient quotes.

Symbols: =, equals to; et al, and others.

Abbreviations: AAMCP = Adherence with Asthma Management for Caregivers of Preschoolers, ADAPT = Adolescence Adherence Patient Tool, App = Application, BB = BostonBreathes, CARAT = The Control of Allergic Rhinitis and Asthma Test, CI = Confidence Interval, DHI = Digital Health Intervention, EMD = Electronic Monitoring Device, EMM = Electronic Medication Monitoring, F2F = Face to face, HCP = Health Care Professional, IMS = Instant messaging software, iTRACC = Improving Technology-Assisted Recording of Asthma Control in Children, MARS = Medication Adherence Rating Scale, mHealth = Mobile Health, MMH = MyMediHealth, *N* = Number, NHS = National Health Service, N/A = Not Applicable, QoL = Quality of Life, RCT = Randomised Controlled Trial, RTMM = Real-Time Medication Monitoring, SD = Standard Deviation, SAMS = Smartphone Asthma Management System, SE = Standard Error, SMS = Short Message Service, T = Timepoint, Tel = Telephone, UK = United Kingdom, USA = United States of America.

### Study quality

According to the MMAT criteria, the two mixed-methods and eight quantitative RCT studies presented the most quality concerns, and the three qualitative and two quantitative descriptive studies presented little to no quality concerns (see appendix 2). A consistent issue across study designs was lack of transparent reporting, particularly within the methodology, such as the intervention development process, intervention fidelity, and how asthma preventer and reliever medications were targeted differently. The mixed-method studies received an average rating of 12.5% for ‘Yes’ and 37.5% for ‘Somewhat’, with quality concerns relating to an inadequate rationale for using a mixed-methods design, and lack of integration between data types (i.e. qualitative and quantitative data addressed different research questions or outcomes). The RCT studies received an average rating of 42.5% for ‘Yes’ and 25% for ‘Somewhat’, with key quality concerns revolving around lack of (or failing to report on) blinding, randomisation or allocation concealment. Also, some RCTs reported marginal or substantial differences in socio-demographic factors or drop-out rates between intervention and control groups. Whereas the quantitative descriptive and qualitative studies received an average rating of 100% and 93.3% for ‘Yes’, respectively; thus, presenting with little to no quality concerns according to MMAT.

### Medication adherence - quantitative findings

Eight quantitative RCT studies, two mixed-methods studies, and two quantitative descriptive studies presented quantitative findings for medication adherence (*N* = 12) (see Table 1). Except for the mixed-methods study that did not assess statistical significance for its quantitative results (Cushing et al., 2016), all remaining quantitative studies (*N* = 11) reported at least one significant finding in relation to medication adherence. Nonetheless, due to the mixed quantitative results on medication adherence across and within studies (see Figure 2), along with quality concerns (*N* = 10), inclusion of pilot or feasibility studies (*N* = 5), and lack of reporting of effect sizes for results (*N* = 9), the confidence in the quantitative evidence is relatively low. However, it was possible to calculate effect sizes in some cases (*N* = 3), and Kosse et al., (2019b) only reported effect sizes for their non-significant findings (Kosse et al., 2019b). Based on available data or calculations, studies demonstrated medium to large effect sizes (*N* = 5).

*Between-groups.* Five RCTs reported significantly greater self-reported or device-recorded adherence in the (total) intervention group compared to (total) controls (Chan et al., 2015; Chen et al., 2020; Gao et al., 2024; Johnson et al., 2016; Vasbinder et al., 2016), one of which reported a large effect size (Gao et al., 2024). However, Chen et al. (2020) reported a significant improvement in device-recorded, but not self-reported, medication adherence. Interestingly, Chan et al. (2015) also found that the intervention group reported significantly better adherence in the morning, whereas the control group (i.e. EMD without audio-visual reminders) reported better adherence in the evening.

*Within-groups.* Two quantitative descriptive studies reported significant improvements in medication adherence from baseline to final follow-up in the total study sample, with medium effect sizes (Hashi et al., 2019; Ramsey et al., 2023). Hashi et al. (2019) reported this finding with self-reported medication adherence and from timepoint 1 to 2 (after 4 weeks), whereas Ramsey et al. (2023) reported this finding with device-recorded medication adherence from timepoint 1 to 3 (after approx. 17 weeks) when participants received the appropriate intervention according to the stepped care model. However, Ramsey et al. (2023) did not observe this effect when comparing timepoint 1 to 2 (after 8 to 12 weeks), and three studies found no significant improvement in medication adherence from baseline to final follow-up in their total study or intervention group (De Simoni et al., 2021; Kosse et al., 2019b; Wiecha et al., 2015). Additionally, Cushing et al. (2016) reported an improvement in medication adherence but did not assess statistical significance, whereby a lower proportion of participants reported non-adherence (due to: i–forgetting or ii -controlled symptoms) at follow-up (i–80%, ii–40%), compared to baseline (i–100%, ii–74.43%).

However, some studies reported significant improvements in medication adherence within specific sub-groups, concerning clinical risk, level or direction of engagement, or level of stepped care (De Simoni et al., 2021; Kosse et al., 2019a; Kosse et al., 2019b; Ramsey et al., 2023; Wiecha et al., 2015). Two studies found a significant improvement in the high-risk subset of the intervention group with low medication adherence at baseline (MARS ≤ 19 or device-recorded medication < 75%) (Kosse et al., 2019b; Wiecha et al., 2015), but this effect was not observed for the low-risk subset (MARS ≥ 23) (Kosse et al., 2019a). Moving on to engagement, Kosse et al., 2019a found that users sending messages to pharmacists and logged activity on the pharmacist chat app feature within the intervention group was associated with significant improvements in self-reported medication adherence. Interestingly, this effect was not observed when the messages were sent by the pharmacists. De Simoni et al. (2021) only found significant improvements in device-recorded medication adherence with reminders and incentives for users who were able to pair the EMD with the app, receive reminders and self-monitor throughout, with a medium effect size. Kosse et al. (2019a) additionally reported that average-frequency app users (>10 and ≤ 25 times) had significantly higher rates of medication adherence compared to low-frequency app users (≤10 times), with a medium effect size. Surprisingly, no other comparisons between high (>25 times), average and low frequency app users led to significant differences in medication adherence.

Furthermore, Ramsey et al. (2023) also reported a significant improvement in medication adherence from timepoint 1 to 3 within the subset of study sample that progressed onto step 2, with a large effect size. But they did not observe an effect when comparing baseline to timepoint 2 for the step 2 sub-sample or when comparing baseline to any follow-up timepoint for the step 1 only sub-sample. Finally, some studies reported a (significant) decline in medication adherence over time (from 5 weeks to 12 months after baseline) in their total study sample (Chan et al., 2015; De Simoni et al., 2021; Vasbinder et al., 2016) or within a specific subgroup (only received Step 1, Ramsey et al., 2023).

### Medication adherence - qualitative findings

Three studies (Kan et al., 2021; Nichols et al., 2020; Schneider et al., 2020) presented qualitative evidence on medication adherence. Despite having fewer studies (*N* = 3) and one of which being a pilot, confidence in the qualitative evidence for medication adherence is higher due to providing consistently positive findings and performing well in the critical appraisal via MMAT. Overall, studies reported positive effects of their DHI on medication adherence in most or some participants. Medication reminders improved the consistent and timely administration of medication adherence (Kan et al., 2021; Schneider et al., 2020) and education on inhaler use improved correct administration (Nichols et al., 2020).

### Quality of life

Three RCT studies investigated patient QoL (see appendix 3). Although studies used similar self-reported measures (i.e. PAQLQ), this review’s confidence in the evidence for QoL was low due to insufficient data on significant findings (and no effect size reported) and concerns around study quality.

*Between-groups*. One study reported that patient QoL scores significantly increased in the intervention group, compared to the control group (Johnson et al., 2016). In contrast, two studies found no significant difference in patient QoL scores between the intervention and control groups (Kosse et al., 2019b; Vasbinder et al., 2016).

### User-experiences

Ten studies captured the experiences of patients or caregivers on using their DHI (see appendices 3 & 4), of which, some were pilot or feasibility studies (*N* = 6) and/or had quality concerns (*N* = 6).

*Acceptability.* Many users considered the DHI acceptable, convenient, and practical for asthma management. According to users, the preferred modality for a DHI was a smartphone app, preferred HCP for support was an asthma nurse, and preferred modality for receiving feedback was through SMS messages.

*Usability.* Many users considered the DHI easy to use. However, there were potential difficulties in using multiple devices, when caregivers were unfamiliar with technology and when multiple caregivers were involved in asthma management. Many users experienced technical difficulties, particularly relating to medication reminders & user-monitoring, which hindered user engagement, satisfaction, and the ability to manage asthma. Unsurprisingly, technical support was considered valuable and the ability to use the DHI offline was suggested.

*Engagement.* Users were more engaged at the start of the DHI or with certain features (e.g. user-monitoring) but also expressed interest in the continued use of the DHI after study completion. Also, users engaged more with the DHI after receiving reminders. However, engagement varied across studies. Poor engagement did not always reflect disinterest or dissatisfaction with the DHI since barriers to engagement usually involved technical issues, lack of time, and limited access (e.g. due to phone prohibitions or forgetting to carry the peak-flow). Interestingly, two studies investigated demographic differences in DHI engagement. Johnson et al. (2016) found that a larger proportion of African American participants did not engage with the DHI, and Kosse et al. (2019a) found that female participants engaged with the DHI at a significantly higher frequency and duration than male participants.

*Satisfaction.* Many users reported high levels of satisfaction overall and valued the DHI features available to them, particularly in studies that consulted patients in the DHI design process. Users reported many perceived benefits including improved medication adherence, asthma management, patient health, and communication with HCPs. There were mixed levels of user satisfaction around the specificities of each DHI feature, e.g. the frequency of medication reminders. Generally, users were dissatisfied with the DHI if it didn’t address their concerns, preferences (e.g. how recorded data is visualised), meet/consider their needs (e.g. education was not helpful for users who already understood the importance of adherence), or was inefficient (e.g. tedious password processes & timeouts). Finally, dissatisfaction was often framed as opportunities for improvement, e.g. paediatric patients desired more gamification, customisation, and incentives.

### Theoretical mapping and behavioural analysis

The main behavioural targets addressed by the DHIs were forgetting, poor knowledge or skills related to asthma management, low self-efficacy, and lack of access/uptake of support (see Tables 2 and 3). The behavioural impact of the DHI features and strategies was largely positive, demonstrating their benefit on medication adherence and asthma management overall. However, there were mixed findings on statistical significance and user-experiences and various suggestions for improvement to take into consideration.

The statistically effective digital features for improving medication adherence were reminders (De Simoni et al., 2021), user-monitoring (De Simoni et al., 2021), incentives (De Simoni et al., 2021), and pharmacist chat (Kosse et al., 2019a). These digital features were commonly associated with the TDF domains of Memory, attention, and decision processes, Environmental context & resources, Intentions, Behavioural Regulation, Skills, Social influences, and Reinforcement. However, the digital features that weren’t associated with significant improvements in medication adherence were user-monitoring of medication adherence and asthma control (Kosse et al., 2019a), short movies (Kosse et al., 2019a), and peer chat (Kosse et al., 2019a).

Moreover, the consistently well-praised digital features or behavioural strategies were incentives, tailoring, customisation, gamification, access to user-monitored data and goal setting/tracking. These digital features and behavioural strategies commonly mapped onto the TDF domains of Goals, Reinforcement, Knowledge, Behavioural Regulation, Beliefs about consequences, Beliefs about capabilities, Environmental context & resources, Intentions, and Social Influences. Whereas the digital features or behavioural strategies with mixed appraisal were digital reminders, user-monitoring, education or training, professional monitoring, and peer chats, since they were ineffective for behaviour change in participants who were already adherent, and literate in health & digital technology, or did not adequately address patient concern, needs and preferences.

All individual studies (*N* = 15) mapped onto all three COM-B components and the following TDF domains: Memory, attention, & decision Processes, Behavioural regulation, Skills, Environmental Context & resources, and Intentions. On average, individual studies approximately mapped onto 11 TDF domains, and TDF domains mapped onto 12 studies (see appendices 5 & 6).

**Table 2.** Theoretical mapping and behavioural analysis of DHI features and strategies.

DHI features and strategies	TDF mapping	Behavioural target	Behavioural impact
**Digital reminders** (*N* = 14) Standardised or tailored reminders for medication, outcome assessments & telehealth visits. Users were often reminded on a daily or weekly basis to log data on a digital device.	Memory, attention, and decision processes; Environmental context & resources; Intentions	Forgetting, competing routines, low habit strength, cognitive overload, executive dysfunction	Receiving reminders was associated with statistically significant improvements, with a medium effect size (De Simoni et al., 2021), and quantitative improvements (Johnson et al., 2016; Vasbinder et al., 2016) in medication adherence Digital reminders were considered helpful in improving adherence to asthma medication, especially to preventer inhalers, and in establishing a routine for asthma management, unless asthma was previously well-managed Digital reminders helped users to overcome barriers to medication adherence (e.g. forgetting) and improved engagement with the DHI Some users were satisfied with the mode, frequency, and timing of reminders, particularly when tailored or self-set. Digital reminders via SMS or in-app notifications were broadly acceptable to users However, there was variable engagement, and some users were dissatisfied, preferring less frequent and shorter medication reminders
**Digital user-monitoring** (*N* = 14) Patient or caregiver monitoring of medication intake and additional outcomes (e.g. symptoms, emotions, peak flow value). Users were often expected to log or respond to questions on a daily or weekly basis via a digital device.	Behavioural regulation; Beliefs about capabilities; Skills; Emotion; Environmental context & resources; Intentions	Poor asthma management, low locus of control and self-efficacy, low habit strength	User-monitoring was associated with significant improvements in medication adherence in one study, with a medium effect size (De Simoni et al., 2021), but not another (Kosse et al., 2019a) Digital user-monitoring was considered helpful and beneficial in improving adherence to asthma medication and other clinical outcomes (e.g. asthma control), unless asthma was previously well-managed Many users felt that digital monitoring features improved asthma management overall, by facilitating shared responsibility between patient and caregiver, encouraging independent self-management in adolescent patients with asthma, and supporting communication with HCPs User-monitoring features may have improved the actual ability (i.e. skill) and perceived confidence (i.e. self-efficacy) to manage asthma However, users had variable engagement with this feature and expressed dissatisfaction with the lack of customisation and excessive password entries
**Data-driven digital services and physical resources** (*N* = 12) Access to user-monitored data, including personalised insights or reports, and the ability to compare current entries to previous entries. Users or HCPs receiving (additional) reminders or alerts based on missing medication data or dangerous values for other asthma outcomes (e.g. peak flow). Access to printable structured medication lists and dosing schedule attachable to medication.	Environmental context & resources; Knowledge; Behavioural regulation; Beliefs about capabilities; Beliefs about consequences; Intentions	Poor asthma management, lack of awareness of poor asthma control/status, poor understanding of triggers and patterns of behaviour	Access to user-monitored data, especially in the form of visual/graphic summaries, enables users to track progress in their asthma management, identify behavioural patterns, and contextualise problems. Such data enables caregivers to make informed decisions to avoid asthma exacerbations (e.g. preventing outside play during high-pollen days) Colour-coding user-monitored outcomes facilitated interpretation of data Monitoring adherence to reliever and preventer medications provides meaningful data for patients, caregivers, and HCPs
**Digital or F2F social support** (*N* = 11) Support from caregivers or peers in the form of F2F involvement and digital chats and discussion boards.	Social influences; Social role & identity; Knowledge; Skills	Lack of caregiver involvement in asthma management, lack of access to peer support, little to no exposure to peers with asthma, stigma around asthma and medicine-taking in public	Caregiver involvement is particularly important when supporting toddlers and children with asthma Although there was low or variable engagement with peer support related features, users recognised their benefit in promoting social connection and acceptance Use of peer chat was not associated with significant improvements in medication adherence (Kosse et al., 2019a) Users suggested having age-specific group chats and using celebrity role model testimonials
**Digital or F2F education** (*N* = 10) Education, skills training, or informational resources on asthma (e.g. short movies), the importance or administration of asthma medication (e.g. inhaler technique), or how to use the digital tool(s) (e.g. demonstration videos). Education and skills training was often provided by HCPs or researchers.	Knowledge; Skills; Beliefs about capabilities; Beliefs about consequences; Social influences; Goals; Intentions; Environmental context & resources	Inaccurate health beliefs, incorrect inhaler technique, poor digital literacy	Education and skills training improved users' ability to correctly administer asthma medication and engage with the DHI Users engaged well with and expressed interest in more video-based education and skills training However, users that already have accurate health beliefs (e.g. understand the importance of adherence) or are digitally literate may not benefit from educational features or strategies Short movies were not associated with significant improvements in medication adherence (Kosse et al., 2019a)
**Digital 1:1 professional support** (*N* = 9) Personalised support from HCPs or researchers at designated times (e.g. telehealth visits/sessions), on-demand (e.g. pharmacist chat) or based on user-monitored data. Support can be informational (e.g. constructive feedback), emotional (e.g. encouragement and motivational interviewing), and/or technical.	Social influences; Optimism; Goals; Intentions; Emotions; Knowledge; Skills; Behavioural regulation; Environmental context & resources; Beliefs about capabilities; Beliefs about consequences	Pessimism, low locus of control, lack of personal accountability, lack of awareness/knowledge of how their asthma management can be optimised	Logged activity in the pharmacist chat, especially when initiated by the user/patient, was associated with significant improvements in medication adherence (Kosse et al., 2019a) Patients and caregivers valued the convenience, efficiency and usefulness of two-way communication with HCPs, believing that it has improved their asthma management There were mixed attitudes towards professionals having access to user-monitored data to provide personalised support; some caregivers considered it reassuring, others considered it anxiety-inducing Users preferred to receive professional support from asthma nurses over other HCPs, and preferred to receive support via texting or video chat services Although users valued digital support from HCPs, they don’t believe it should replace F2F care
**Tailoring and modification** (*N* = 6) Tailoring the DHI to befit the personal characteristics or circumstances of the target audience. Modifying the DHI from a previous study or during an iterative process.	Social influences; Knowledge; Intentions; Behavioural regulation; Environmental context & resources; Goals	Poor user-experience, including unmet needs and lack of relevance	DHIs were generally well-accepted, with smartphone-based features and services being particularly praised for aligning with adolescent patient and caregiver use of technology Tailoring and modifications likely improved the efficacy and acceptability of the DHI However, many DHIs highlighted the need for tailoring since some users considered the features or strategies irrelevant or the DHI was considered challenging when caregivers lacked digital literacy or multiple devices were involved in asthma care
**Financial incentives & digital interactivity** (*N* = 5) Financial compensation for study participation or completion. Financial rewards (e.g. gift cards) for medication adherence or engagement with DHI features. Gamification and customisation features, including changing the design of their page, data entry, and personal profile or hero avatar.	Reinforcement; Social role & identity; Memory, attention & decision processes; Environmental context & resources	Low motivation for participation, engagement or adherence, digital features being too boring or not relatable, busy schedules	Financial incentives and gamification helped improve adolescent patients’ motivation and likelihood to remember to adhere to their asthma medication Although not all users engaged with the customisation features, the interactivity of the DHIs was well-praised, particularly the use of quizzes, customisation and gamification Many users considered the financial incentives appropriate and effective Users expressed interest in having more gamification, customisation and interactivity in DHIs, as well as using more modern designs
**Digital goal setting & tracking** (*N* = 4) Goal setting based on user-monitored data against individual asthma action plan or within telehealth behavioural intervention sessions. Goal tracker feature that allows user-monitored data to be compared to personal goals.	Goals; Intentions; Knowledge; Skills; Behavioural regulation; Environmental context & resources	Poor asthma management and action planning, lack of awareness of progress in asthma management	Goal setting and tracking features support users with action planning Integrating goal tracking features into user-monitored data was considered helpful to compare data entries to personal goals over time

Note: Features and strategies were ordered in terms of frequency (i.e. how many studies applied said feature/strategy). Behavioural target refers to the relevant or addressed barriers to each feature or strategy. Behavioural impact refers to the reported effect of each feature or strategy on the outcomes of interest, namely medication adherence and user-experiences. All the digital features and behavioural strategies mapped onto all three COM-B components, thus did not need to be listed in the table.

Symbols: =, equals to. 1:1, one to one.

Abbreviations: COM-B = Capability, Opportunity, and Motivation model of Behaviour; DHI = Digital Health Intervention, F2F = Face to Face, HCPs = Health & Care Professionals, *N* = Number of applicable studies, SMS = Short Messaging Service, TDF = Theoretical Domains Framework.

**Table 3.** Synthesis of findings per research question.

Research question	Synthesised findings
**Primary:** are DHI effective at improving medication adherence in paediatric patients with asthma?	DHIs were effective at significantly improving medication adherence in paediatric patients with asthma in at least one context (compared to baseline, controls, or within specific subgroups) (*N* = 11), with some demonstrating medium to large effect sizes (*N* = 5) DHIs have mixed effectiveness within individual studies at improving medication adherence in paediatric patients with asthma (*N* = 6), these discrepancies resulted from differences in measures (e.g. for device-recorded but not self-reported), timepoints/level of stepped care (e.g. for timepoint 3 but not 2), level or direction of engagement (e.g. for low vs average frequency app users but not low vs high frequency app users) or risk (e.g. for high-risk but not low-risk patients, based on different MARS cut-off points) Based on sub-group analyses (*N* = 5), DHIs were effective at significantly improving medication adherence in paediatric patients with asthma who were poorly adherent at baseline, could access the DHI or engaged well with specific digital features, or progressed onto step 2 of care (with a medium effect size) DHI led to positive quantitative trends in medication adherence but not statistically assessed (*N* = 1) DHIs were considered effective at improving medication adherence according to qualitative data (*N* = 3)
**Secondary:** are DHIs effective at improving QoL in paediatric patients with asthma?	DHI was effective at significantly improving QoL in paediatric patients with asthma, when compared to controls (*N* = 1) DHIs were not effective at significantly improving QoL in paediatric patients with asthma, when compared to controls (*N* = 2)
**Secondary:** what are the user-experiences of paediatric patients with asthma or their caregivers with DHIs?	Many users positively appraised their experience with the DHI in terms of usability, acceptability, and satisfaction (*N* = 10) Negative user-experiences mostly related to suboptimal usability and engagement, and dissatisfaction around specific aspects of the DHI features (*N* = 10)
**Secondary:** what digital features or behavioural strategies were well-praised and effective for behaviour change (i.e. improving medication adherence) and what are their associated theoretical constructs?	The statistically effective digital features for improving medication adherence were reminders, incentives, and pharmacist chat (*N* = 2), associated with the TDF domains of Memory, attention, and decision processes, Environmental context & resources, Intentions, Behavioural Regulation, Social Influences, and Reinforcement The most consistently well-praised digital features and behavioural strategies were incentives (*N* = 3), tailoring (*N* = 2), customisation (*N* = 2), gamification (*N* = 2), access to user-monitored data (*N* = 9), and goal setting/tracking (*N* = 1), associated with the TDF domains of Goals, Reinforcement, Knowledge, Behavioural Regulation, Beliefs about consequences, Beliefs about capabilities, Environmental context & resources, Intentions, and Social Influences

Note: The findings from the randomised or descriptive quantitative studies (*N* = 10), the qualitative studies (*N* = 3), and the mixed-methods studies (*N* = 2) were synthesized per research question.

Symbols: =, equals to, ?, question mark.

Abbreviations: DHI = Digital Health Intervention, MARS = Medication Adherence Rating Scale, *N* = Number of applicable studies, QoL = Quality of Life, TDF = Theoretical Domains Framework.

## Discussion

### Principal findings

The current mixed-mode systematic review explored the effectiveness of DHIs in improving medication adherence and QoL in paediatric patients with asthma. All included studies delivered multi-component interventions, involving multiple target audiences (i.e. patients & their caregivers, *N* = 4), devices (*N* = 13), features or strategies (*N* = 15), or stepped care (*N* = 1). All eleven quantitative studies that conducted statistical analyses, reported significant improvements in medication adherence in at least one context: either compared to controls (*N* = 5), baseline (*N* = 2), or only within specific sub-groups (*N* = 4): participants that could access and engage well with the DHI (*N* = 2) or had low baseline adherence (i.e. high risk) (*N* = 2). For some of these findings, studies were able to demonstrate medium to large effect sizes. To caveat, there were observable mixed results across and within studies and relatively low confidence in the quantitative evidence, including the study that reported positive quantitative trends without assessing statistical significance (*N* = 1). The qualitative findings, with a relatively high confidence in evidence, favourably supports the effectiveness of DHI in improving medication adherence (*N* = 3). However, there was insufficient evidence and low confidence in evidence to determine the effectiveness of DHIs in improving QoL in paediatric patients with asthma; since only one of three studies reported a significant improvement.

Additionally, this mixed-mode systematic review explored the user-experiences of patients and/or their caregivers, and the appraisal and efficacy of digital features and behavioural strategies for behaviour change and their associated theoretical constructs. Across the ten studies that assessed user-experiences, the DHIs were generally well-accepted, with many users reporting positive experiences concerning ease of use, perceived benefits on multiple outcomes, and high overall satisfaction. Nevertheless, some negative user-experiences were reported, typically relating to sub-optimal usability due to technical difficulties, low or variable engagement, and dissatisfaction around the specificities of digital features. The statistically effective digital features were reminders, incentives, and pharmacist chat (*N* = 2), most associated with the TDF domains of Memory, attention, and decision processes, Environmental context & resources, Intentions, Behavioural Regulation, Skills, Social Influences, and Reinforcement. The most consistently well-praised digital features and behavioural strategies were incentives, tailoring, customisation, gamification, access to user-monitored data and goal setting/tracking, most associated with the TDF domains of Goals, Reinforcement, Knowledge, Behavioural Regulation, Beliefs about consequences, Beliefs about capabilities, Environmental context & resources, Intentions, and Social Influences. User-monitoring had mixed findings statistically and qualitatively, and the following digital features or behavioural strategies also had mixed qualitative findings: reminders, user-monitoring, education or training, professional monitoring, and peer chats.

### Medication adherence

In this review, all included studies demonstrated improvements, quantitatively or qualitatively, in medication adherence in all or some of their participants because of their multi-component DHI (*N* = 15), informed by theory and/or co-design (*N* = 8). Statistical significance was established in most of the quantitative studies (*N* = 11), and some of these findings had medium to large effect sizes (*N* = 5). This positive finding is consistent with the wider literature on similar DHIs for improving medication adherence in (paediatric) patients with various chronic health conditions (Bezabih et al., 2021; Modi et al., 2026; Shih & Cohen., 2020). Nevertheless, since most studies failed to report effect sizes for their quantitative findings (*N* = 10), had quality concerns (*N* = 11), or were pilot or feasibility studies (*N* = 6), evidence should be interpreted cautiously.

Additionally, the mixed results for medication adherence highlight key methodological considerations, rather than evidence against DHIs. The mixed results within and across studies suggest that the effectiveness of DHIs may necessitate or be contingent on providing a higher level of stepped care (Ramsey et al., 2023), users accessing and being engaged with the DHI (De Simoni et al., 2021; Kosse et al., 2019a), or targeting high-risk paediatric patients with asthma (Kosse et al., 2019b; Wiecha et al., 2015). Meaning, due to the diverse nature of asthma and varied levels of engagement, one should expect to find discrepancies in medication adherence results—and these may map onto the distinct phenotypes for paediatric asthma and other socio-demographic factors. Although Chen et al. (2020) reported a discrepancy in adherence results that support the reliability of device-recorded over self-reported measures, future researchers are still encouraged to adopt a multitude of measures for medication adherence, such as triangulation, to corroborate the efficacy of their DHI.

The digital features that were consistently well-praised and statistically effective for improving medication adherence were financial incentives and pharmacist chat. These two features typically targeted the barriers of low motivation and lack of access to personalised professional support and commonly mapped onto the TDF domains of Environmental context & resources, Reinforcement, Social influences, Intentions, Behavioural Regulation, and Memory, attention & decision processes. These findings affirm previous literature demonstrating the efficacy of financial incentives and pharmacist-led digital features on improving medication adherence in patients with chronic health conditions (Bell et al., 2026; Tran et al., 2022). Financial incentives were beneficial in (i) increasing motivation to adhere to asthma medication, (ii) implicitly reminding users to take medication, and (iii) encouraging engagement with digital features and devices. The pharmacist chat was beneficial, particularly when initiated by the adolescent patient with asthma, since it offers an accessible and timely opportunity to ask questions and receive personalised answers and feedback. Therefore, future DHIs should consider delivering both digital features, caregivers might consider providing financial incentives to encourage their children to adhere to their asthma medications, and health services should facilitate for adolescent patients with asthma to contact their local pharmacist for support. However, further research is needed to better understand different types of incentives (e.g. monetary vs non-monetary), approaches (e.g. gain vs loss framing), and target behaviours (e.g. medication adherence vs study completion) and their impact on behaviour change in paediatric asthma (Kenyon et al., 2022).

The digital features and behavioural strategies that were consistently well-praised but not necessarily statistically analysed were customisation, gamification, access to user-monitored data, goal setting/tracking, and tailoring. These features and strategies targeted various barriers, including, poor understanding of behavioural patterns, asthma management, action planning/behavioural regulation/problem-solving, and negative user-experiences. They were also associated with TDF domains of Goals, Knowledge, Intentions, Behavioural regulation, Environmental context & resources, Beliefs about consequences, Beliefs about capabilities, and Social influences. The literature supports the acceptability, perceived benefit and even efficacy of these digital features and strategies for improving medication adherence in patients with other chronic health conditions (Adeghe et al., 2024; Mair et al., 2023; Tran et al., 2022). Many users valued customisation and gamification features, but it is still unclear whether they improve medication adherence directly or indirectly by encouraging more user engagement with DHI features that positively impact adherence. Future DHIs should consider integrating incentives into gamification and educational features, where improvements in engagement and medication adherence correspond to a point system unlocking non-monetary rewards (e.g. stickers) at predefined milestones (Grossman et al., 2017).

Moreover, accessing user-monitored data and goal setting/tracking features were considered beneficial for improving adherence to both preventer and reliever medications and asthma management overall as they enable users to: (i) review progress (i.e. compare current entries to previous entries and personal goals), (ii) identify behavioural patterns (e.g. asthma exacerbations occurring more often on high-pollen days), (iii) plan their actions/regulate their behaviour/problem-solve accordingly (e.g. switching to indoor play on high pollen days), and (iv) conveniently and accurately communicate or share medical data with HCPs. Thus, future DHIs are recommended to include access to user-monitored data and integrate customisation and goal setting/tracking features into user-monitoring. Additionally, tailoring during design and delivery (i.e. modification) was jointly beneficial in ensuring positive user-experiences and the delivery of relevant targeted support effective for behaviour change. For example, like Ramsey et al. (2023), future DHIs should consider delivering telehealth behavioural sessions tailored to the unique barriers of the patient (or their caregivers) to improve their medication adherence; albeit this personalised approach is more time-consuming and has limited scalability. Overall, more research is needed to statistically assess the differentiated impact of tailoring, customisation, gamification, goal setting/tracking, and access to user-monitored data on medication adherence, particularly since access to data (*N* = 11) was not provided by all studies that delivered user-monitoring features (*N* = 14).

The digital features and behavioural strategies with mixed results regarding statistical significance or appraisal by users were digital reminders, user-monitoring, professional monitoring, education or training, and peer chats. These features and strategies typically targeted the barriers of forgetting, low habit strength, poor asthma management, low understanding of health (outcomes), and lacking ability to action plan/regulate behaviour/problem-solve. These features dynamically addressed various TDF domains, including Memory, attention, and decision processes, Skills, Behavioural Regulation, Knowledge, Social Influences, Environmental context & resources, Intentions, and Emotions. Despite several studies establishing a (statistically) quantitative improvement in adherence to preventer inhaler medication due to digital medication reminders (*N* = 3), this feature had mixed appraisal. Digital medication reminders were only considered effective for users who needed support with reminding or habit formation, but not for users who were already adherent. This is validated by two of the included studies that only found significant improvements in medication adherence in patients who were poorly adherent at baseline (Kosse et al., 2019b; Wiecha et al., 2015). Reminders were particularly useful when tailored to the users’ expected time of administration, delivered after missed doses, or set by the users themselves. Interestingly, Chan et al. (2015) found that the intervention group that received digital reminders were more adherent to preventer inhalers in the morning than in the evening, which is a pattern supported by the wider literature (Almonacid et al., 2021). Therefore, paediatric patients with asthma or their caregivers who are struggling with medication adherence are recommended to set up morning digital medication reminders.

Similarly, user-monitoring had mixed results concerning statistical significance and user appraisal; although user-monitoring benefits some, it provides little value for users with well-established routines and well-managed asthma. Nonetheless, this review demonstrates how monitoring a variety of outcomes - including adherence to preventer and reliever inhalers, emotions, symptoms, peak flow value, and environmental factors - can provide context and convenience to asthma management (e.g. how peak flow value is related to asthma status and emotional state) and facilitate collaborative care between patients and caregivers. Future researchers may wish to extend current understanding of asthma management by enabling the monitoring of additional medications (e.g. antihistamines for allergic rhinitis), and a broader spectrum of environmental factors (e.g. family smoking status) and health behaviours (e.g. physical activity, eating habits, and sleep hygiene). Also, future DHIs could benefit from better utilisation of research on habit and contextual factors to support asthma management and long-term maintenance of medication adherence (Badawy et al., 2020; Kenyon et al., 2022), particularly since some studies in this review observed a decline in adherence over time (*N* = 4).

Additionally, professional monitoring, education, and training, including skills training and motivational interviewing, received mixed appraisal. These features and strategies, delivered digitally or F2F, were considered beneficial when participants had low self-efficacy, incorrect inhaler technique, inaccurate health beliefs (e.g. believing adherence to preventer inhaler is unrelated asthma control), low digital literacy or low motivation to adhere to medication. However, these features and strategies were not beneficial to participants who were already digitally literate or understood how to administer their medication and why it is important. To account for the user preferences and concerns highlighted in this review, future DHIs should consider recruiting nurses or pharmacists to deliver informational or emotional support via video (for generic education or skill-based demonstrations) or text-based formats (for personalised comments or feedback), in conjunction with F2F care. Moreover, some users considered professional monitoring (i.e. HCPs or researchers accessing user-monitored data to provide personalised support) reassuring and helpful, whilst other users considered it anxiety-inducing, in line with the wider literature in relation to data privacy concerns (Baruffati et al., 2026; Giebel et al., 2023). Future DHIs may wish to consider opt-out options for features that involve professional monitoring or data-sharing to overcome barriers to participation.

Finally, the peer chat and discussion board features had relatively poor engagement and did not statistically yield significant results. This was an unexpected finding since paediatric patients with asthma noted their concern with stigma around medicine-taking in front of their peers (Nichols et al., 2020) and the value of digital peer support features was recognised in this review and among other paediatric patient populations in the wider literature (Liverpool et al., 2020). Rather than discrediting the utility of digital peer support features, future researchers should consider designing age-specific group chats or providing celebrity role model testimonials, as suggested by users, to better determine their efficacy on medication adherence and QoL. Overall, the mixed findings on digital reminders, user-monitoring, professional monitoring, and education/training are inconsistent with research that evidences their efficacy in other chronic health conditions (Kim et al., 2025; Smith et al., 2023; Xu et al., 2025) but corresponds to research that highlights that DHIs need to address the unmet needs and preferences of the target audience to be effective (Wilson et al., 2024). Therefore, to ensure user engagement and effective behaviour change, future DHIs should consider amending their eligibility criteria or screening participants to ensure the recruitment of paediatric patients with asthma or caregivers with unmet needs and/or experience hard to access services. Also, future research should conduct a needs assessment prior to intervention development to identify patient or caregiver barriers to medication adherence and draw on existing literature to identify relevant and effective BCTs (Mair et al., 2023).

### Quality of life

Two of the three RCT studies that quantitatively assessed patient QoL reported no significant difference between the intervention and control groups despite reporting significant differences in medication adherence, either between groups (Vasbinder et al., 2016) or within-groups for a high-risk subset (Kosse et al., 2019b). This is inconsistent with previous literature (Snoswell et al., 2021) and may be due to ceiling effect, where paediatric patients with asthma reported high levels of QoL at baseline (Kosse et al., 2019b). Alternatively, paediatric patients with asthma may have required specific digital features or behavioural strategies to observe an effect on their QoL, in other words, there may be a missing active ingredient (Vasbinder et al., 2016). Johnson et al. (2016) reported significant improvements in QoL in the intervention group, compared to controls, despite having a closer follow-up and using similar DHIs and controls to Vasbinder et al. (2016), since both studies delivered text-based time-tailored reminders and user-monitoring. However, unlike Johnson et al. (2016), Vasbinder et al. (2016) also provided financial compensation, access to data, onboarding instructions and video, and technical support. Although Johnson et al (2016)’s findings are more consistent with previous reviews on DHIs for patients with asthma (Snoswell et al., 2021) and other chronic health conditions (Lorca-Cabrera et al., 2020), more research is needed to determine whether these additional digital features and behavioural strategies impact patient QoL. More generally, it was surprising to discover that most studies did not assess QoL considering its well-observed association with asthma control (Jaimini et al., 2018; Lee et al., 2018) and medication adherence (Belachew et al., 2023; Kosse et al., 2020; Valero-Moreno et al., 2023). Overall, based on the data available, this review cannot conclusively determine the efficacy of DHIs on QoL in paediatric patients with asthma.

### User-experiences

The DHIs were considered an acceptable intervention for improving medication adherence in paediatric patients with asthma, which aligns with previous literature (Butler et al., 2020; Cheng et al., 2022; Gilbey et al., 2020). However, the consistently positive results on acceptability, largely based on pilot or feasibility studies, may be indicative of selection effect, whereby data is heavily based on motivated users that participated long enough, due to having a favourable experience of the DHI, to report their favourable opinions. Future researchers are encouraged to collect data on reasons for withdrawal to contextualise drop-out rates and determine the impact of selection effect. Overall, users perceived practical (e.g. more convenient asthma management), personal (e.g. improved self-efficacy, skills, and knowledge), and clinical benefits (e.g. improved asthma control and communication with HCPs; and fewer exacerbations/hospitalisations) to engaging with the DHIs. However, the reported user-experiences highlight key design and research considerations.

During the development of future DHIs, researchers should account for the target patients’ developmental (i.e. NHS banding), and medical needs (e.g. asthma control), as well as the educational needs of patients and their caregivers (e.g. health beliefs & digital literacy). The digital features explored in this review could be further optimised with more automation (e.g. date of entry for user-monitoring) (Vitija et al., 2022), modernisation (e.g. app design), and better consideration of user needs, preferences (e.g. customisation) (Abraham et al., 2019), and lifestyle compatibility. For example, since the use of multiple digital (network) devices was considered challenging or inconvenient (Kan et al., 2021; Schneider et al., 2020), future researchers should seek to develop novel technologies that integrate the distinct utilities of multiple devices and that could be usable off-line and at school or work. Overall, future DHIs should adopt evidence-based theoretical frameworks (Morrison, 2014) and patient-based approaches (Yardley et al., 2015) involving co-design and tailoring to ensure optimal user-experiences and efficacy of their DHIs (Malloy et al., 2023; O’Connor et al., 2023).

Furthermore, since user engagement varied widely across studies and was associated with significantly better medication adherence in average frequency-app users, compared to low frequency app users, with a medium effect size (Kosse et al., 2019a), more research is needed to further investigate and understand the causes of low engagement and explore effective strategies for enhancing engagement. Notably, included studies observed lower engagement patterns in African American and male participants compared to their white and female counterparts, which corresponds to previous research demonstrating poorer uptake of health services and psychosocial support amongst these populations (Bamidele et al., 2022; Ogundare et al., 2026; Tshuma et al., 2024). Moreover, researchers should further explore the impact of socio-demographic factors, including internet accessibility and health literacy (e.g. Secchi et al., 2022), and income or employment status (e.g. Hashi et al., 2019), on uptake and efficacy of DHIs to contextualise health inequalities and identify opportunities for personalisation and additional support (Farre et al., 2023). Since technical issues may have adversely affected the efficacy of DHIs and led to unfavourable user-experiences, future researchers are recommended to conduct usability testing of their DHI, using ‘inquiry’ methods with potential users - such as think-aloud studies (e.g. Ramasawmy et al., 2024) - and ‘inspection’ methods with experts (Maqbool & Herold, 2023). Also, researchers should provide digital literacy education during onboarding, especially for grand-caregivers, technical support throughout DHI delivery (Schneider et al., 2020) and surveys to evaluate usability post-delivery (Teufel et al., 2018).

### Strengths and limitations

This review provides an updated and comprehensive overview of DHIs for improving medication adherence in paediatric patients with asthma across multiple study designs, different types or modalities of DHIs and the paediatric age range, as defined by the NHS. However, there were limitations. For example, excluding papers that assessed medication adherence not as a primary outcome and non-English papers could have resulted in the omission of potentially relevant findings. Future researchers ought to consider a more lenient inclusion criteria on outcomes, leverage translation services to overcome language barriers and consider the impact of having eligible studies from mostly high-income countries on the generalisability of their findings. Furthermore, study quality was assessed using MMAT and resulted in multiple quality concerns, including inadequate rationale and reporting. Despite offering researchers the ability to suitably appraise studies with different designs, the MMAT tool makes it challenging to determine study quality, which is needed to effectively compare studies and assess eligibility for sensitivity analysis, in which the tool offers little guidance for. Authors extended the MMAT and considered additional factors to determine confidence in evidence; however, future reviewers are encouraged to formally assess certainty of evidence, such as using the Grading of Recommendations Assessment, Development, and Evaluations (GRADE) tool (Guyatt et al., 2008).

Although a narrative synthesis allows for a rich and exploratory approach to data analysis, it is more prone to bias due to its subjective nature; nevertheless, the use of two independent reviewers mitigated this risk. To support the narrative synthesis and conceptualisation of user-experiences, researchers categorised this outcome into four distinct dimensions, despite the lack of standardised definitions and assessments and these dimensions being closely related, theoretically and practically (Newton et al., 2021; Perski & Short, 2021). This review highlights the utility of and the need for more research on multi-faceted theoretical conceptualisations of user-experiences to help guide future intervention design and evaluation (Mclaughlin et al., 2021; Perski & Short, 2021; Perski et al., 2017). A meta-analysis, which offers a more statistically robust analysis of data, was not conducted due to the heterogeneity of studies (though not formally assessed) and being deemed unsuitable considering the descriptive aims. However, future reviewers are recommended to meta-analyse their behavioural outcomes, and where relevant and feasible, to differentially meta-analyse sub-groups to determine the statistical impact of each digital feature or behavioural strategy across different clinical characteristics and levels of engagement. Considering data was not meta-analysed, and to avoid resorting to the discouraged practice of vote counting (McKenzie & Brennan, 2019), authors applied the SWiM guidance, such as reporting effect sizes, and produced harvest plots to visualise heterogenous quantitative data on medication adherence. Moreover, this review highlights the need for researchers to develop accessible guidance on how to create harvest plots and share best practices to minimise the risk of misinterpretation.

The researchers mapped the DHIs of included studies onto the COM-B and TDF and applied a theoretical behavioural lens to understand how the DHIs had been planned, developed, and implemented, offering potentially useful guidance for DHI design to ensure content is clearly defined and effective for behaviour change. However, both models are criticised for their over-simplification (i.e. reduces broad concepts into few theoretical constructs) and overlap (i.e. the inter-connectedness of distinct constructs) (Francis et al., 2012; Ogden, 2016; Schenk, 2022). For example, the COM-B was not useful in differentiating between different types of DHIs, digital features, or behavioural strategies, since they all mapped onto all three components. Although using the same theories to map and analyse facilitates the comparability of DHIs, this approach does have its setbacks; it may overlook: (i) nuanced contextual factors not adequately or uniquely addressed by the COM-B & TDF and (ii) the contributions of other theoretical models and frameworks within individual studies, such as the common-sense model of self-regulatory theory (Reynolds et al., 2012) in Kosse et al (2019b)’s study. Without a detailed BCT coding, the practical benefit of our theoretical mapping and behavioural analysis for future DHI design is somewhat limited.

### Future research

To strengthen the evidence base, higher quality theoretically informed longitudinal studies are needed to determine the efficacy of DHIs in improving and maintaining medication adherence in paediatric patients with asthma. Since asthma is theorised to have distinct phenotypes, researchers are encouraged to collect relevant clinical or environmental contextual data to better identify clinical risk, consider potential moderators of intervention success, and understand health behaviours within the broader context of medical history. For example, adolescent patients with type 2 or 4 asthma may benefit most from DHIs that involve user-monitoring for adherence, symptoms and environmental factors, as both types have late-onset allergic rhinitis. Moreover, more research is needed to statistically and descriptively determine which digital features or behavioural strategies are most effective at promoting medication adherence and QoL and ensuring positive user-experiences in paediatric patients with asthma. Despite caregiver involvement being presumed for younger paediatric patients, more research is needed to explore this type of social support in adolescent patients with asthma. Since this review highlighted additional challenges for grand-caregivers, future researchers may wish to explore the impact and usability of DHIs on different types of caregivers (e.g. eldest child, parent, grandparent, childminder). Importantly, in light of recently announced UK government plans to provide stronger protections in schools for students with allergies (Bailey., 2026), future DHIs should consider targeting teaching and pastoral staff since asthma medications are often stored or administered in schools, particularly reliever inhalers for emergencies.

Additionally, researchers need to be more consistent and transparent with their reporting of the development process and intervention itself to allow reviewers to better synthesise and appraise their results. For example, Gao et al. (2024) reported using the Global Initiative for Asthma (GINA) guidelines to inform the content of their asthma education for caregivers. Moving forward, researchers are recommended to utilise, and report, policies and guidelines related to DHIs and asthma management to develop evidence-based DHI for paediatric patients with asthma, and accounting for environmental considerations, such as assessing economic and environmental benefits of DHIs (Faizan et al., 2025; Vasbinder et al., 2016). Moreover, research funds should prioritise investing in DHIs designed and delivered in low- and middle-income countries to tackle their underrepresentation and address the unique needs of paediatric patients with asthma and their families in these countries (Mortimer et al., 2022). Finally, future researchers should extend this review’s contributions by creating a taxonomy of effective BCTs in DHIs for improving medication adherence in this population.

## Conclusions

This review contributes to the literature surrounding medication adherence in paediatric patients with asthma by synthesising recent findings, and applying a behavioural analytical approach, to support future intervention design. This review also highlights implications and recommendations for individuals and families to better manage paediatric asthma and for future researchers to address limitations and gaps in the literature. To avoid over stating real-world effects and account for the limitations, this review concludes that DHIs are potentially acceptable and effective at improving medication adherence in paediatric patients with asthma, particularly when addressing the unmet needs and barriers of patients or their caregivers and using features like financial incentives and pharmacist chat. Whereas, for QoL, the evidence available was insufficient to determine the efficacy of DHIs on this outcome for this patient group. Overall, more research, particularly co-designed, theoretically informed multi-component DHIs, using RCT or mixed-method longitudinal study designs, is needed to consolidate and extend the findings from this review. Due to the rapid pace of technological advancements in the field of DHIs, researchers are recommended to regularly review the literature for any updates and developments.

## Supplementary Material

Revised Supplementary Materials not for review.docx Revised Supplementary Materials not for review.docx

## Data Availability

The data that support the review’s findings can be found in the supplemental material provided.

## Open Scholarship

This article has earned the Center for Open Science badges for Open Preregistered. The data are openly accessible at registration of study design and sampling plan of the larger project are openly accessible at https://www.crd.york.ac.uk/PROSPERO/view/CRD42022320097; registration of the hypotheses and analysis plan are openly accessible at [https://www.crd.york.ac.uk/PROSPERO/view/CRD42022320097].

## Appendices

### Appendix 1. Intervention characteristics of included studies

**Table:**

Study	Name, Who & Why	What & How	When, Where & How much	Tailoring, How Well & Modifications	Comparator
***Mixed methods** (N = 2)*					
Cushing et al. (2016)	**Name**: novel mHealth management system **Target audience:** adolescents **Aim:** to improve teenagers' ability, motivation, and triggers to adhere to asthma medication **Theory/development:** the system is informed by behavioural theory - Fogg (2009), which was applied to the context of asthma	**Mobile app:** patient can view their usage and send reminders. People with lower adherence receives more reminders. This iOS app is downloadable on an iPhone or iPad. The home screen shows the time of the previous medication use. It also illustrates with graphs the patient's daily, weekly, and monthly medication adherence. The display can be downloaded and printed. The mobile app includes a customizable “*hero”* avatar feature and an incentive scheme of up to $50 over the study period, with bonus points for longer-term adherence. **Device:** the inhaler adherence sensor helped to transfer data to the server. A silicone sleeve attaches to asthma inhaler and dispenser. When medication is taken the sensor records the time of medication use and uploads data using Bluetooth to the app for storage on a HIPAA -compliant server. The sensor devices were present to allow storage of 50 data points (~2 weeks of data)	**When:** the inhaler device should be used daily **How much:** the frequency of reminders was not specified, and it was unclear whether it depended on adherence. However, participants reported receiving multiple reminders during the day **Where:** RW setting (NR but expected)	**Tailoring:** Tested the usability and/or quality of their DHI. **How Well:** one participant's data could not be uploaded as they were unable to sync the inhaler to the iPad. Two patients reported faults with the inhaler caps. There were also faults with the length of the cable which loosened the cap as a result. **Modifications:** NR	The control group received the inhaler sensor and the app but was restricted to just data transfer with no reminders or rewards.
De Simoni et al. (2021)	**Name:** an electronic reminder and incentives intervention **Target audience:** adolescents **Aim:** to improve adherence in adolescents with severe asthma **Theory/development:** the intervention was grounded on the PAPA framework. It was also informed by results from a former qualitative study, meetings with stakeholder, and PPI.	Smartphone app: participants could pair the device with the app. Data to see medication data was able to see via transfer through Wi-Fi. Participants can receive electronic reminders. The reminders could be set by the participants themselves within the app and they could also set this to come through the app or the device. Financial incentive: incentive payments were based on how participants took their medications and how well they adhered to their medication. The incentive was capped at £ 2 per day 56 days for a maximum to £ 112 in gift cards	**When:** the intervention lasted 24-weeks **How much:** the incentive scheme took place over 8 weeks and started from the visit two, during which patients were told about their incentive phase. **Where:** RW setting (NR but expected)	**Tailoring:** Tested the usability and/or quality of their DHI. **How well:** the EMD-Hailie app sometimes presented faults with pairing. In this case, adherence data could be downloaded. There was also a help section on the app where participants could receive technical support. **Modifications:** NR	N/A
***Quantitative RCTs** (N = 8)*					
Chan et al. (2015)	**Name:** EMD with audio-visual reminder feature **Target audience:** children **Aim:** to examine the medication adherence and asthma outcomes of an EMD with audio-visual reminders on children who regularly attend the emergency rooms **Theory/development:** NR	**Device:** the device (SmartTrack) has been approved by the US FDA and it records specific data for the administration on inhaler use. It provides ringtones (14 distinct types of ringtones) where it alerts the users twice a day. The reminder continues to ring for 15 minutes or until the user has administered the correct dose.	**When/how much:** reminders were scheduled to ring twice per day, every day. The reminders rang until the correct does had been taken or after 15 minutes. **Where:** RW setting (NR but expected)	**Tailoring:** Tested the usability and/or quality of their DHI. **How well:** the devices were checked to ensure the quality control of the features. **Modifications:** NR	The control group received an EMD with the audio-visual reminder feature disabled.
Chen et al. (2020)	**Name:** electronic monitoring, weekly feedback, & reminders **Target audience:** children & their caregivers **Aim:** to increase adherence to preventer inhaler in younger children **Theory/development:** NR	Device: a chip was attached to the inhalers (SmartTrack) to track medication administration. It tracks the date, time, and number of doses taken. Data automatically transfers to the server by Bluetooth. The asthma nurse monitored each week’s data. App: WeChat was used as a messaging service which works on smartphones. If users had poor adherence rates the nurse would provide feedback through this service. Education: the caregivers were provided educational feedback sessions from a nurse on their inhaler technique. The importance of taking medications correctly was made most aware within these sessions.	**When/how much:** the nebulizer is used daily, with reminders from nurses. Weekly feedback from nurses to caregivers. **Where:** RW setting (NR but expected)	**Tailoring:** NR **How well:** NR **Modifications:** NR	The control group received an EMD without feedback and reminders. Otherwise, just like the intervention group, adherence data was collected and calculated weekly and caregiver reports were assessed monthly.
Gao et al. (2024)	**Name:** Telephone & SMS Intervention **Target audience:** caregivers **Aim:** to improve compliance with preventer inhaler in children living with asthma **Theory/development:** NR	Three groups: Telephone + SMS group (first intervention group) SMS group (second intervention group) Control group Education **:** all three groups received basic education on asthma according to GINA regiment. Medication: all three groups received budesonide/formoterol powder (80 μg/4.5 μg) for inhalation. Medication reminders **:** In both intervention groups, an educational SMS message was sent to caregivers to remind and help them to better supervise their child during medication inhalation. The Tel + SMS group also received telephone notice of a revisit. Both the SMS group and control group received notice of their next visit verbally. Revisit: Inhalation technique was examined for accuracy at each revisit. Outcome assessment: all three groups underwent assessment for asthma control level and lung function.	Education **:** delivered F2F before enrolment. Medication: one inhalation twice a day. Medication reminders **:** delivered via SMS to caregivers before each dose (2 SMSs a day) for both intervention groups. Telephone notice for revisit: to caregivers in treatment weeks 1, 4, & 12 Outcome assessment: before the start of follow-up and 12 weeks later. **Where:** clinical setting (Asthma Outpatient Department of the Affiliated Hospital of Qingdao University)	NR	The control group did not receive SMS or telephone intervention. They received general guidance for the medication during the first visit and revisits. Also, they were informed of the next visit date verbally.
Johnson et al. (2016)	**Name:** text-message based reminder system **Target audience:** adolescents **Aim:** to improve adherence to asthma medication and self-efficacy in adolescents. **Theory/development:** MMH was developed through the results of focus groups, user testing, and revisions from eight families. The research team tested the project before launching it.	MMH **:** delivered on a web application designed to run on a tablet or desktop computer. The system automatically sends a text message to the number provided. The patient can create a secure profile so that the system sends a text message to the number for a verification response. Upon verification, the patient can log in and create a medication list and schedule. All medication names are coded using the RxNorm standard. Features: there were tools for patients to create and print a structured medication list, attach a dosing schedule to each medication, request a text message reminder for each dose, and visualize medication adherence performance for each medication in the system. It also provides features such as a *“vacation*” feature that uses prescription information (dose count) entered by the patient to determine if a refill will be needed before a particular date. MMH also records natural language responses and user assertions about medication use. Users may reply to a reminder by typing a letter: (T)aking, (S)kipping, or (H)olding a dose one time. When a dose is taken or skipped, MMH creates a note in a medication log. When a dose is held, MMH asks the user when s/he expects to take the drug (in hours from the time of response) and automatically generates a reminder for that time. Caregivers were compensated at the rate of $20 at enrolment and completion of the trial. Adolescents were compensated at the rate of $20 at enrolment and $40 at completion of the trial. However, intervention participants were responsible for any text message costs. Protocol and procedure: A protocol was followed where participants were provided Instructions by email, including a 24/7 support helpline, a demo video, and detailed directions for the text message system. A research coordinator staffed a “help desk” and monitored participant activity for the first week, contacting inactive participants to troubleshoot and document problems.	**When/how much:** participants could request when the reminders set with the choice of many reminders of different medications sent within one text message. There is an option for the medication to be held up until the next scheduled does. The web app can be accessed 24/7. **Where**: clinical setting (paediatric outpatient settings at an academic medical centre)	**Tailoring:** Time-tailored doing remidnders **How well:** NR **Modifications:** NR	The control group received usual care and informational resources.
Kosse et al. (2019a)	**Name:** ADAPT **Target audience:** adolescents **Aim:** this was not reported; however, it was likely that it was to improve adherence to asthma medication in adolescents. **Theory/development:** the development of the intervention used a patient-centred approach and was influenced by a theoretical framework that was not reported.	The ADAPT app: the app was connected to a desktop application for the pharmacist. The different features of the app are described below: (1) Questionnaire (CARAT) to monitor symptoms: to monitor the control of asthma, which was made available weekly and both patients and HCPs could review the overall score The CARAT is made up of 10 questions where a total score between 0 and 30 could be obtained (>24 indicated good disease control). Patients could receive feedback for their results. (2) Medication alarm: to remind participants to take their medications. However, it was not linked to the inhaler and so rang even if a patient had already taken the dosage. (3) Short movies: to educate and motivate the patient. Topics were based on the management of asthma. Patients could see on the app when one was made available. Pharmacists could send other movies to different patients based on the patient’s needs. (4) Peer chat: to allow adolescents to talk with other peers without being moderated. This feature was recommended at the developmental stages. There were 6 topics for discussion: general, pets, going out, sports, asthma, and other. (5) Pharmacist chat: patients and pharmacists could contact one another through the chat feature. (6) Adherence questions: there was the opportunity for patients complete two questions weekly which were based on the MARS questionnaire.	**When/how much:** (1) Questionnaire (CARAT) to monitor symptoms: participants received 26 notifications to remind them to complete the questionnaire. (2) Medication alarm: this could be set to ring when each participant preferred. (3) Short movies: one movie appeared in the app per week. Over the 6-month period 21 movies became available. (5) Pharmacist chat: participants had access to the chat feature all the time. HCPs received emails when a message was sent to them. (6) Adherence questions: every two weeks two questions on medication adherence appeared on the app for the participants to complete. **Where:** RW setting (NR but expected)	**Tailoring:** NR **How well:** NR **Modifications:** NR	Details of the control group were not fully reported.
Kosse et al. (2019b)	**Name:** ADAPT **Target audience:** adolescents **Delivery:** pharmacists were the chosen healthcare provider to deliver the intervention because in the Netherlands patients generally use one pharmacy to collect all their medication prescriptions. The community pharmacists were affiliated with the UPPER. **Aim:** to support adherence in adolescents, by targeting several aspects of non-adherence behaviour. **Theory/development:** the DHI was developed with adolescents and HCPs. It was grounded in the Common-Sense Model of Self-Regulation theory.	Patients received a smartphone app which connects to the desktop app for the pharmacist. The app includes numerous features which focus on non-adherent behaviours: (1) questionnaire to monitor control of asthma, this was made available weekly and both patients and HCPs could review the overall score (2) reminders (3) informational and motivational video movies (4) chat feature to allow users to contact other peers from the study (5) pharmacist chat where users could contact their HCP (6) manual entry questions; one question was about forgetting (unintentional) and one was on the intentional decision to miss a dose. Pharmacists could send more movies to patents and contact the patients whilst monitoring their scores on the CARAT.	**When/how much:** questions appeared on the app once every two weeks. The intervention was accessed for six months. **Where:** RW setting (NR but expected)	**Tailoring:** NR **How well:** NR **Modifications:** NR	The control group received usual care.
Vasbinder et al. (2016)	**Name:** RTMM SMS **Target audience:** children & their caregivers **Aim:** to improve adherence to preventer inhaler, asthma control, QoL, and decrease likelihood of asthma exacerbations **Theory/development:** NR	e-MATIC: a RTMM was provided to all children, and this was connected to a pMDI which records the administration details. This transfers data automatically to the study database in real-time. Children within the intervention group received the RTMM and reminders.	**When/how much:** if a dose had been missed a tailored SMS reminder was sent after 15 minutes. **Where:** RW setting (NR but expected)	**Tailoring:** SMS reminders were time tailored **How well:** NR **Modifications:** NR	The control group received RTMM without tailored SMS reminders.
Wiecha et al. (2015)	**Name:** BB **Target audience:** children & their caregivers **Delivery:** HCPs were expected to provide care in either the control or intervention groups. **Aim:** (1) to improve adherence to preventer inhaler in children. (2) to improve communication between HCPs and patients. (3) to improve HCPs understanding of patient health between consultation visits. **Theory/development:** the DHI was based on social cognitive theory and eHealth theoretical models. The design procedure followed usability frameworks.	The DHI is a web-based system which provides education, communication, and tracking. Every user receives a handheld PIKO digital peak flow meter with information on how to use it and the zones. Their peak flow was measure at HV1 and was displayed on the website which was coded. Red was for patients with a FEV1 on < 50%, yellow between 50-80%, and green above 80%. This then provided feedback to the user. If the measures kept exceeding the values, it was adjusted to reflect the highest number. An alert was sent to the staff when a patient was in the red zone. Children and caregivers were trained at home by a research assistant who reviewed BB login procedures, established passwords, supervised data entry of current medications and doses into the website and populated an asthma action plan visible on the website. Children were asked to navigate through the features of the website to demonstrate competence and then to make a posting to discussion boards. Caregivers were encouraged to participate with their children in the use of the website including educational activities Patient Website: every time a user logs in to tebsite they can report their symptoms, peak flow measure, missed school days, A&E visits, and limitations towards activities in their daily life. Animation was also provided where information can be viewed. Users can. Also track and report medication adherence and track their progress. Educational fun activities such as quizzes and puzzles were available to the patient along with a library full of informational resources. Users were able to view other users’ personal pages for motivation, partake in a discussion on the peer discussion board, and have discussions with HCPs. The webpage was made so that it could be updated and changed by the user. Primary Care Provider Website: the website for providers could monitor participants, review, and monitor asthma status and peak flow, symptoms, and view A&E visits. HCPs could use the discussion board to discuss topics with their patients. They also received alerts for when patients where in the red zones. Financial incentives: when the participant used each feature, they received an incentive. The reward points were displayed on the home screen The rewards were turned into gift cards. Summary forms of data for PCPs: HCPs could access a separate web app which helped them to access summaries of data.	**When/how much:** the website can be accessed at any time by either the patient or their caregiver. HCPs were notified via email when a patient had logged into the DHI. with a hotlink to their login page on BB. Data from the participants was reviewed every two months by the teams’ specialist. Specialised feedback and recommendations were then posted back to the patient via private discussion boards. At HV1, surveys were completed and the DHI was set up. At HV2, surveys were completed again and the Doser was picked up. The participants were also trained to use the DHI at this visit. **Where:** RW setting (home)	**Tailoring:** NR **How well:** NR **Modifications:** NR	The control group received usual care, information resources, and a peak flow meter.
***Quantitative descriptive** (N = 2)*					
Hashi et al. (2019)	**Name**: web-based diaries intervention **Target audience:** caregivers **Aim:** to assess the effects of maintaining web‐based diaries by the caregivers of pre-schoolers with asthma on the adherence to asthma care regimens, the acknowledgement of symptoms, and knowing their levels of stress, QoL, and family functioning. **Theory/development**: the DHI was pilot tried and tested	Diary entries **:** caregivers were asked to submit one to two daily entries Forgotten entries were to be forgotten for the full day. The entries needed to include things such as symptoms (asthma attacks, wheezing, chest tightness, etc.), activities in daily life, activity, medication administration, sleepless nights/disturbed sleeping, etc. Due to the study taking place in autumn and winter, seasonal allergies and weather was expected to be discussed too. Researcher feedback **:** researchers could provide feedback on user feedback by repeating and confirming their comments. They never provided educational feedback only supportive. Web-based data collection: Using their own devices, users could access a survey online	**When/how much:** caregivers were to manage their child’s asthma as usual and submit daily entries for one month. Researchers provided feedback weekly. Caregivers received daily reminders at 8:30 am to complete their daily diary entries. **Where:** RW setting (NR but expected)	**Tailoring:** NR **How well:** NR **Modifications:** NR	N/A
Ramsey et al. (2023)	**Name:** Technology Assisted Stepped Care Intervention **Target audience:** adolescents Assisted Stepped Care Intervention **Delivery**: trained psychology doctoral trainees & respiratory therapist **Aim:** to improve adherence to preventer inhaler in adolescents with moderate to severe-persistent asthma **Theory/development:** the intervention was adapted from a school-based telehealth adherence promotion intervention for adolescents with asthma (Lin et al., 2020)	**What:** Stepped-care intervention involves a hierarchy of interventions so that individuals move through (from Step 1 to Step 2) based on systematically assessed needs to ensure they receive the lowest intensity treatment needed to be effective. Participants received Cohero® mobile tracking sleeves (Cohero Health, Inc.) for their inhalers and an Android smartphone with a prepaid data plan and the mobile applications required for the study. Training: Participants received training from a respiratory therapist using ATS guidelines (Miller et al., 2005) on using a mobile spirometer. Step 1 - Digital (Medication) Reminders: Participants received digital medication reminders through the BreatheSmart™ app and digital push notification to complete mobile spirometry readings through the MedaCheck Habit™ app. BreatheSmart™ app transmitted the adherence data from the Cohero mobile tracking sleeves. Adherence monitoring continued during Step 1, but no adherence feedback was provided. Threshold to escalate: Participants whose average adherence was below 68% qualified for Step 2. Step 2 - Telehealth Behavioural Intervention: Participants received telehealth behavioural intervention sessions and access to adherence feedback via the BreatheSmart™ app. The manualized intervention sessions included self-monitoring strategies, discussions of individual barriers to adherence and allocation of treatment responsibility, organizational strategies to improve adherence, and guided problem-solving training.	Step 1 - Digital (Medication) Reminders **:** medication reminders received daily, lasted approximately 8 weeks M (SD) = 8.23 (3.73). Push notifications for mobile spirometry readings provided weekly for the duration of the study M (SD) = 4.48 (1.58) months. Adherence rate calculation: After 4 weeks of receiving digital medication reminders Step 2 - Telehealth behavioural intervention sessions/feedback: 4 weekly, lasted approximately 5 weeks M (SD) = 5.23 (1.76). Outcome assessment: At baseline, participants completed questionnaires with study staff. Participants were contacted by study staff to assess monthly composite severity scores and asthma control. **Where:** clinical setting (patients were approached by a research team member during an outpatient pulmonary asthma clinic appointment)	**Tailoring:** within Step 2, the guided problem-solving training was individually tailored to the adolescent’s unique barriers to adherence. **How well:** within Step 2, intervention sessions included fidelity checks for the initial 10 sessions and 20% of sessions thereafter. **Modifications:** NR	N/A
***Qualitative** (N = 3)*					
Kan et al. (2021)	**Name:** multicomponent EMM-based iTRACC intervention **Target audience:** children & their caregivers (dyads) **Aim:** to track daily intake of preventer inhaler **Theory/development:** NR	Bluetooth-enabled sensors, app, and web portal: patients received sensors which were attached to the inhaler and automatically transfers data to the smartphone, caregiver’s mobile app, and web portal. HCPs would provide calls to caregivers from the data they receive. This would track most available medications for both preventer and reliever medications. Caregivers could also enter adherence manually too. Caregivers could set up reminders at specific times for when they were planning to administer daily preventer inhaler medication. If a medication dose were missed, a push notification would alert them. Emails would be sent to HCPs in the patients change in dosages/timing. HCPs could then call caregivers to discuss the changes. The apps could provide local allergen information and reports.	**When/how much:** the mobile app can be accessed at any time by the caregiver. Caregivers can set up daily reminders at their preferred times. There are daily reports given. **Where:** clinical setting	**Tailoring:** Parents set up timed reminders for administering daily ICS medications and were notified by push notifications from the app when medications were missed. **How well:** NR **Modifications:** NR	N/A
Nichols et al. (2020)	**Name:** SAMS app **Target audience:** children **Delivery:** an asthma educator trained in motivational interviewing performed the telehealth visits. **Aim:** to improve paediatric asthma self-management. **Theory/development:** the development of the DHI was based on SDT.	Real-time asthma assessment: the app was downloaded to the user’s personal phones. The EMA was provided for two months to track asthma control, symptoms, medication use, and emotional state. Inhaler caps could provide data so that participants could view their progress. End user-guided provider interactions: participants could interact with HCPs via telehealth visits or by sending videos of inhaler technique. Reminders: reminders via SMS were sent to users to help them to remember to complete the EMA questions. It also allowed them to remember to take part in telehealth visits and when they needed to video capture inhaler technique. End user-guided provider interactions: the participant could perform the inhaler technique video via the app. When this was registered, they could see on-screen prompts to technique. The video was securely transferred to be observed by an asthma specialist educator/respiratory therapist. Feedback could them be delivered via SMS and/or telehealth visits.	**When/how much**: real-time asthma assessment: DHI users were to answer questions in the EMA daily. End user-guided provider interactions: within the two-month study period, participants were expected to use each of the HCP interaction features at least once. At week four the telehealth visits were to be provided at times that are best for the participant. Reminders: reminders were only sent if data was absent for three consecutive days. **Where:** RW setting (home and school)	**Tailoring:** Intervention was modified during the iterative design and development phase. **How well:** NR **Modifications: t**here were changes made to the data collection. Due to technical difficulties, the devices were removed. The DHI protocol was consistently updated due to participant feedback	N/A
Schneider et al. (2020)	**Name**: asthma self-management mobile app **Target audience:** adolescents **Aim of intervention:** to improve self-management in adolescent living with asthma. **Theory/development:** the DHI was based on contributions from adolescents and HCPs on their initial thoughts. The prototype was guided by asthma treatment guidelines from NIH’s NAEPP EPR.	System messages: users would receive app reminders and inspirational messages. They could also track progress and make personal goals within the system. Participants received training in peak-flow meters as per recommendations in standards of care. Peak flow readings were stored and were able to be viewed graphically. Users could view their current and previous personal goals. Peak-flow process: during this process, the subject can report their feelings by choosing an emoji face which represents their emotions, symptoms, and peak-flow measurements. Notes can also be made so users can keep track of their own thoughts/comments. Participants can receive feedback reports on their entry and measurements. The DHI would then understand the NIH NAEPP EPR 3 asthma status of each patient based on their individual peak-flow goals and reported symptoms. Asthma status was shown visually with colour coding (green specifies 80%–100% of the individual’s goal peak-flow value, yellow shows 50-80%, and red shows < 50%). Users could receive a personalised plan based on this. Profile Options: participants could change their design options on the profile page whenever they desired.	**When/how much:** the trial period lasted three months. Every user was reminded twice to enter their asthma symptoms into the app. Participants also received reminders if their readings were low. Reminders could be adjusted if needed. **Where:** RW setting (NR but expected)	**Tailoring:** the mobile app was tailored to participants' age. Questions were provided to participants of different age groups, and genders to understand unique design preferences. **How well:** NR **Modifications:** NR	N/A

Note: Studies were grouped by study design and ordered in alphabetical order of first author's surname within each grouping. Data was extracted using the TIDieR checklist (Hoffman et al., 2014), but overlapping categories were grouped for brevity.

Symbols: %, percentage; =, equals to; >, greater than; <, less than; &, and; /, or; μg, microgram; £, pound; $, dollar; ~, approximately; et al, and others.

Abbreviations: App = Application, ADAPT = Adolescence Adherence Patient Tool, A&E = Accident & Emergency, ATS = American Thoracic Society, BB = BostonBreathes, CARAT = The Control of Allergic Rhinitis and Asthma Test, DHI = Digital Health Intervention, etc = et cetera, EMA = Ecological Momentary Assessment, EMD = Electronic Monitoring Device, EMM = Electronic Medication Monitoring, EPR = Expert Panel Review, eMATIC = electronic-Monitoring of Asthma Therapy to Improve Compliance in children, F2F = Face to Face, FDA = Food and Drug Administration, FEV1 = Forced expiratory volume, GINA = Global Initiative for Asthma, HCP = Health Care Professional, HIPAA = Health Insurance Portability & Accountability Act, HV = Home Visit, iOS = Iphone Operating System, iTRACC = Improving Technology-Assisted Recording of Asthma Control in Children, M = Mean, MARS = Medication Adherence Report Scale, mHealth = mobile health, MMH = MyMediHealth, *N* = Number, NR = Not Reported, N/A = Not Applicable, NAEPP = National Asthma Education and Prevention Program, NIH = National Institute of Health, PAPA = Perceptions & Practicalities Approach, PCP = Primary Care Practitioner, pMDI = pressurised metered-dose inhaler, PPI = Patient & Public Involvement, QoL = Quality of life, RCT = Randomized Controlled Trial, RTMM = Real-Time Medication Monitoring, RW = Real World, SAMS = Smartphone Asthma Management System, SD = Standard Deviation, SDT = Self-Determination Theory, SMS = Short Message Service, TM = Trade Mark, UPPER = Utrecht Pharmacy Practice Network for Education & Research, US = United States, Wi-Fi = Wireless Fidelity.

### Appendix 2. Summary of results on study quality

**Table:**

Study	S1	S2	Q1	Q2	Q3	Q4	Q5	%
***Mixed methods** (category 5) (2 studies)*								
Cushing et al. (2016)	No	No	No	Can’t tell	Somewhat (qualitative and quantitative findings were reported separately, with limited integration of results and no visual representation of findings)	NA (qualitative and quantitative measured different things that complement one another - no triangulation needed)	Can’t tell (the authors lack transparent reporting of methods and qualitative results are not supported by participant quotes)	Y - 0%; SW—25%
De Simoni et al. (2021)	Yes	Yes	No	Somewhat - (qualitative and quantitative data addressed different research questions)	Somewhat (qualitative data addressed the acceptability of the intervention and quantitative data addressed the outcomes of the intervention including adherence)	NA (qualitative and quantitative measured different things that complement one another - no triangulation needed)	Yes	Y - 25%; SW—50%
***Quantitative RCTs** (category 2) (8 studies)*								
Chan et al. (2015)	Yes	Yes	Yes	Yes	Yes	Can’t tell	Can’t tell	Y - 60%; SW—0%
Chen et al. (2020)	Yes	Yes	Yes	Yes	Yes	Somewhat (caregivers and nurses were not blinded, but doctors and statisticians were)	Somewhat (slightly different dropout rates between intervention and control group, but no evidence of crossover)	Y - 60%; SW—40%
Gao et al. (2024)	Yes	Yes	Yes	Yes	Somewhat (complete data for 95 participants, despite 120 being enrolled, so just under 80% - > 79.1%)	Can’t tell	Somewhat (slightly different dropout rates between the three groups, but no evidence of crossover)	Y - 40%; SW—40%
Johnson et al. (2016)	Yes	Yes	Can't tell - (mentioned block randomisation, but no mention of blinding or allocation concealment)	Yes	Yes	Can’t tell	Somewhat (slightly different dropout rates between intervention and control group, but no evidence of crossover)	Y—40%; SW—20%
Kosse et al. (2019a)	Yes	Yes	Can't tell	Can’t tell	Yes	Can’t tell	Can’t tell	Y - 20%; SW—0%
Kosse et al. (2019b)	Yes	Yes	Can’t tell	Yes	Yes	No	Somewhat (different dropout rates between intervention and control group, but no evidence of crossover)	Y - 40%; SW—20%
Vasbinder et al. (2016)	Yes	Yes	Yes	Yes	Yes	Somewhat (although physicians, researchers, and patients were initially blinded for randomisation, patients were generally unblinded shortly after the start of the study period)	Somewhat (slightly different dropout rates between intervention and control group but no evidence of crossover)	Y - 60%; SW—40%
Wiecha et al. (2015)	Yes	Yes	Can't tell (randomisation methods were not reported. Also, they originally had three groups but were then merged)	Somewhat (different sized groups and aside from parental education, there were no significant differences between groups at baseline)	No	Yes	Somewhat (slightly different dropout rates between intervention and control group but no evidence of crossover)	Y - 20%; SW—40%
***Quantitative descriptive** (category 4) (2 studies)*								
Hashi et al. (2019)	No	Yes	Yes	Yes	Yes	Yes	Yes	Y - 100%
Ramsey et al. (2023)	Yes	Yes	Yes	Yes	Yes	Yes	Yes	Y - 100%
***Qualitative** (category 1) (3 studies)*								
Kan et al. (2021)	Yes	Yes	Yes	Can't tell (methods are not well justified)	Yes	Yes	Yes	Y - 80%
Nichols et al. (2020)	No	Yes	Yes	Yes	Yes	Yes	Yes	Y - 100%
Schneider et al. (2020)	Yes	Yes	Yes	Yes	Yes	Yes	Yes	Y - 100%

Note: Studies were grouped by study design and ordered in alphabetical order of first author's surname within each grouping.

The screener questions for the mixed methods appraisal tool (Hong et al., 2018) were as follows: Are there clear research questions? Do the collected data allow to address the research questions? The remaining five questions differed between study designs.

The answers to the screener questions were coded yes or no/not clear. The answers for the criteria questions were coded as ‘Yes’, ‘No’, ‘Somewhat’, ‘Can’t tell’ or ‘Not applicable’.

Percentages were calculated using the frequency of ‘Yes’ responses, and separately, ‘Somewhat’ responses, in relation to the number of (applicable) criterion questions. Questions with a not applicable response were not included in percentage calculations.

Symbols & abbreviations: et al = and others, *N* = no or not clear, Q = Question, RCT = Randomised controlled trial, S = Screener, SW = Somewhat, Y = Yes.

### Appendix 3. Summary of results on secondary outcomes

**Table:**

Study	Patient QoL	User-experiences
***Mixed methods** (N = 2)*		
Cushing et al. (2016)	NR	**Measure**: unstructured interviews **Results**: Participants reported that they received numerous reminders in the day and when they were at school but did not have their phone with them the reminders were still taking place. Suggestions for less frequent reminders when they were not being seen and less content within the messages. Three people explained that the DHI helped to remind them to adhere to their medication. They explained that if they continued using the DHI after the study period, they would tell other people living with the condition about the app. Patients said that they felt that the messages on the app reminded them about the medications that are usually forgotten especially when there are many medications to take daily. Participants stated that the DHI helped them to change behaviours and have a better routine which increased their confidence in controlling their asthma. The gaming features were valued as well as the reward system which allowed them to be motivated to adhere. The patients liked to monitor their progress. Through a points system, a graph, or displaying a number value. Two participants suggested that the app should have features where they can unlock certain levels to interact with their hero avatar more.
De Simoni et al. (2021)	NR	**Measure**: exit interviews, workshop and online survey **Results**: Most participants felt the intervention helped them to take their preventer inhalers more regularly due to reminders and self-monitoring within the app. All adolescents in the survey agreed the intervention could help improve inhaler treatment. Technical difficulties were shown to be an issue which decreases their chance of taking their medication. Participants wanted incentives as they felt this would help them be motivated to take their preventer inhaler. Adolescents agreed that the intervention could help improve adherence to inhaler treatment, with most suggesting £ 1 per dose and gift cards to collect rewards. They felt the incentives would increase motivation for taking inhalers and partly work as a reminder.
***Quantitative RCTs** (N = 5)*		
Johnson et al. (2016)	**Measure:** Mini PAQLQ **Results:** in the intervention group QoL increased from a median of 5.7–6.3 in comparison with the control group (*P*-value = 0.037).	**Measure**: usage data, including website activity and text message reminders/responses, and a usability survey. **Results**: 6 (13%) participants never used the DHI. One adolescent’s mobile service provider did not work with the text messaging service. 1 (adolescent) could not use the reminder feature. 20 participants used the medication list to add preventer medication and 18 users added reliever inhaler and allergy medications. Users added a median of 2 (SD = 1.4) medications to their medication lists (range: 0–5). Just over half of the participants used the message feature for at least one text message. There were some users who had no issues receiving or responding to reminders. Three users have problems with this but had this resolved within the first week of the study. Eight users had problems responding to reminders throughout the study. Five users managed reliever medications only and had no problems doing so. 21 (46%) users had access to reminders, and 17 people used this feature. Participants received a mean of 12 reminder alerts for the two weeks. Participants took their preventer inhaler medication daily on an average of 10 occasions in two weeks. 18 (39%) patients set up the system with an aim to improve adherence to reliever inhaler medication. Five participants logged their use of a reliever inhaler. One non-user said that DHI “*didn’t work on my phone.”* Two people thought making medication selections were “*confusing.”* One subject had issues with responding to reminders for medications and after trying three times stopped. They were still able to receive and respond to reminders that were set up manually. (78%) of participants and 18 (86%) of non DHI participants wanted to continue to use MMH. Participants were very satisfied with the DHI even though the usage was varied. Depending on the feature of the DHI, the participants attitude towards the DHI was rated between neutral to positive.
Kosse et al. (2019a)	NR	**Measure**: usage data **Results**: 87 participants had the app downloaded and of these 86 used the DHI 1975 times. Median app use per person was 17 times (IQR 6-31; range 1-113) over five months (IQR 3-6; range 0-8). It was found that females were using the DHI more than their male counterparts (median 20.5 vs 11; *P*-value = 0.01) and over six months (median 5 vs 6 months; *P*-value = 0.03). Adolescents used, on average, 3 different features of the app (IQR 3-4; range 1-5). All 5 features were used by 13% (11/87) of the adolescents. High-frequency users included more females than low-frequency users (73% [19/26] vs 44% [12/27], *P*-value = .04). Low-frequency users: 93% (25/27) completed CARAT; 56% (15/27) ≥ 1 adherence assessment; no peer chat use. Additionally, high-frequency users: 81% (21/26) messaged pharmacists and 77% (20/26) watched videos (significantly higher vs other groups). **Adherence questions:** 83% (72/87) completed ≥ 1 adherence assessment (*N* = 221); median = 2 (IQR 1–4; range 1–11) of 14 possible. **Questionnaire to monitor symptoms:** This feature was used the most with 85 (98%) of adolescents completing the questionnaire. **Short movies:** Approximately half of the users watched one movie at the very least. Overall, 21 videos where available in the app; however, a mean of four different videos were watched per person (IQR 2-6; range 1-20), and each movie was seen once (IQR 1-1; range 1-4). The movies which were on the app first had the most views. **Pharmacist chat:** 75% of people used the feature with an average of three messages (IQR 1-7; range 1-37), overall number of 347 messages. Pharmacists mostly started the chat; and half did not receive a response. 82% of pharmacists sent messages with a median of 2 messages per adolescent (IQR 1-5; range 1-20). 12 users who did start the discussion first, one-third four did not receive a message back (reasons unknown). 38 adolescents (44%) sent on a mean number of two messages (IQR 1-5; range 1-17). Overall, 34 conversations were held at least once. **Peer chat:** This was the least used feature of the DHI. 21% of users used this feature and sent an average of 150 messages. Each user sent 4.5 messages (IQR 3-11; range 2-29). The most frequent message themes were that of sports (67 messages from eight subjects), age, school, and home life (34 messages from six adolescents), and about the study (24 messages from eight users).
Kosse et al. (2019b)	**Measure:** PAQLQ **Results:** no intervention effects were observed on the scores (*P*-value > 0.05)	NR
Vasbinder et al. (2016)	**Measure:** PAQLQ **Results:** there was no significant difference between the groups at follow up based on adjusted means	NR
Wiecha et al. (2015)	NR	**Measure:** patient and provider usage of the website & surveys **Results**: Subjects were highly satisfed with the DHI with all domains having mean agreement scores of 8.5 or higher on a Likert scale of 0 (strongly disagree) to 10 (strongly agree). This was for the appearance, the ease of use, the information usefulness, and the ease of a learning system. DHI users accessed the intervention a mean score of 7 times in the first month of the study period. The average duration was 19 minutes. Most chats were between participants and nurse, and less with the other HCPs. Encouragement to take medication and guidance with using the DHI was the most frequent themes found within the messages. Then it was about difficulties with the peak flow readings. Explicit coordination of care represented about 5% of messaging content. Participants earned an average of $6 (range, $0-$40) in rewards for using the website in the 6 months. Providers agreed that the asthma specialist feedback was useful as it was easy to use. For the discussion board feature, participants responded with neutral comments. Three themes were found: improvements with communication between HCPs and patients, having the access to check their patient’s overall health, and a concern for the time it takes to use the system.
***Quantitative descriptive** (N = 2)*		
Hashi et al. (2019)	NR *(reported caregiver QoL)*	**Measure**: participants were asked for their impressions of the intervention following the conclusion of the study **Results**: Six subjects who dropped out did not provide any feedback. Two excluded participants complained about not receiving reminder e-mails over the duration of the study. Participants noted the ease of use. For example, “*It’s easier than a paper asthma diary,”* and *“Daily e‐mails (reminder mail) were good.”* Example of a negative comment made by a participant: “*I’m bothered to enter a password every time,”* and *“I’m bothered to enter the date”*
Ramsey et al. (2023)	NR	**Measure:** feasibility was assessed by calculating enrolment, participation, and retention rates throughout the study. Acceptability, usability and satisfaction were assessed using a 12-item quantitative questionnaire (0-4 scale, 4 being most positive). **Results**: Overall, the enrollment, retention, and participation rate each surpassed a prior feasibility criteria. Enrollment rate = 93.75% (two individuals declined participation out of 32 individuals contacted). Retention rate = 83.33% (one individual switched to an incompatible inhaler that required manual adherence tracking, thus were excluded from adherence analysis). Retention rate for Step 1 = 87% (26 out of 30 enrolled individuals completed Step 1). Enrollment for Step 2 = 69% (18 of 30 enrolled individuals were escalated to Step 2). Retention rate for Step 2 = 94% (17 out of 18 individuals enrolled for Step 2 completed it). Demographic differences: no significant differences in demographic variables (*P*-values > .05) between individuals that remained on Step 1 (*N* = 8) and those requiring Step 2 (*N* = 18). Participation/Engagement rate for Step 2 = 100% (all 17 individuals escalated to Step 2 attended all four behavioural sessions) Participants generally rated their study experience positively (average acceptability = 3.32). 52% rated their experience as “excellent” and 32% as “good.” Only one participant (4%) rated their experience as “poor.” Most participants (64%) expressed satisfaction with the DHI. Regarding specific aspects of the intervention, 75% of average ratings surpassed the acceptability threshold of 3.0.
***Qualitative** (N = 3)*		
Kan et al. (2021)	NR *(reported caregiver QoL)*	**Measure**: interviews **Results**: six-major themes were revealed concerning caregivers' experiences with the DHI (EMM-enhanced): **Theme 1: Compatibility with the family's lifestyle.** The technology was found to be “*easy*”. Caregivers liked that they could track their inhaler use when their child was at school. Caregivers reported that the app alerts were well-timed and served as a reminder during hectic days. A barrier was the involvement of grand-caregivers unfamiliar with technology. **Theme 2: Impact on asthma management.** Caregivers expressed that the DHI improved their management of asthma via preventer and reliever medications. Medication reminders and establishing a routine helped with daily preventive management. If a reliable routine was already established, the DHI did not lead to behavioural changes but was still appreciated by caregivers. Whereas for acute management, tracking reliever inhaler helped to identify triggers or patterns of asthma exacerbations. Caregivers also described that the app record helped them provide an accurate account to doctors at clinic visits rather than relying on memory. **Theme 3: Impact on the child’s health.** Caregivers believed that the DHI led to improvements in their child’s health, from having more energy to fewer asthma attacks, and shorter-lasting symptoms. However, some caregivers did not observe changes in their child’s health since was already well-managed before the DHI or had improved with age. The DHI helped one caregiver manage their child’s worsening asthma. **Theme 4: Emotional impact.** Caregivers felt confident and secure with a nurse monitoring their child’s medication use and liked when nurses or HCPs would call to bulletuss their child’s symptoms. However, caregivers described frustration due to technical difficulties. **Theme 5: Child's engagement in asthma management.** The sensor and app improved children's engagement in self-management. Caregivers stated that their child was better engaged with taking care of their asthma because they were interested in the technology and app. Caregivers could assign their child a certain aspect of the responsibilities, such as using the sensor cap to take a dose and watching themselves administering it correctly with a light. Children’s engagement with the technology also included monitoring themselves on the app and using the gaming features on the app—doing quizzes, tracking puffs, and reading information. **Theme 6: Recommendations.** Caregivers said that improving the sensor technology’s syncing was important; they reported that the “*synchronizing issue*” was difficult to fix and were uncertain if the sensor had issues “*just a tech issue,”* or was bulletonnecting due to battery issues. Caregivers suggested including a texting service after phone calls for follow ups from HCPs. Caregivers wanted features that would help their child to be engaged in their asthma management. Caregivers also asked that the sensor devices have greater ability to be used with different inhaler medications.
Nichols et al. (2020)	NR	**Measure**: video capture session and telehealth visit during the intervention phase **Results**: All participants took part in one video session, and one telehealth visit during the study phase. **Benefits of smartphone asthma management system** There was positive feedback on usability and modality, e.g. the DHI was easy to use: *"The tool to me is super easy for somebody that’s new to having a child with asthma. It really did help me...it’s walking you through—this is exactly how you are to do it."* [Caregiver #3, phase 2] Reminders: *"[I was] pretty comfortable ‘cause it’s like I knew I took my medicine and...it helps you remember to take your meds...I didn’t wanna get sick".* [Child #6, phase 2] Children like using technology - *"I think that it’s good because like I like computers and stuff and I like to be on the phone a lot." [Child #8, phase 1]”* A recurring theme was the benefits of reminders on medication adherence. For example, “*It helps you remember to take your meds*.” [Child #6, phase 2]. Convenience was another benefit of the DHI. Caregivers felt that SAMS conveniently facilitated communication for all parties considering they usually have busy lifestyles. The use of technology was strongly preferred by most participants as the primary means of routine communication and interaction. Although none of the participants wanted to completely replace traditional face-to-face encounters. For example*, “I think the text messages and alerts, not actually having to sit on the phone with a human and the hold times, the wait times—technology has taken over. I think it’s just so much more simpler.”* [Caregiver #12, phase 1]. Feeling supported and cared for by others were strong attributes of relatedness throughout the intervention. Participants appreciated concern over health and well-being by others. For example, *"[The intervention] reassured me that there is genuine concern about how my son is doing and how he is recovering and whatever other treatments he may need."* [Caregiver #9, phase 1]. Others expressed appreciation in knowing that providers were actively involved and concerned with their child’s well-being and care management. For example, *“That was helpful because...she was kind of doing it wrong. So, when we talked to her, she kind of told us, you know, certain ways you’re supposed to do it and count. That was real helpful.”* [Caregiver #4, phase 2]. **Challenges of smartphone asthma management system** The time required for video uploads especially when waiting to upload one video before administering a second dose. For example, *“So, you are sitting there for like four or five minutes because you take your second puff to get, you know, to film that...it was just a little difficult.”* [Caregiver #3, phase 2]. One mother described how it was difficult to hold the phone and administer and monitor. For example*, “I would have to site my phone in a neutral location, or she would have to get closer to the room in order for it to pick up. As far as I was concerned, that’s the only flaw*. [Caregiver #12, phase 1]. The DHI timing out when videoing. Bluetooth connection and proximity to the smartphone. Children reported the importance of relatedness, underscoring the importance of peer relationships and social acceptance during childhood and adolescent development. Several articulated concerns over being perceived as different, a strong desire to be considered normal, and as preferring to maintain privacy regarding the awareness of their disease with others. For example, *“But once I did it at school because she came and had lunch with me and then people were looking at it and I was mad."* [Child #2, phase 2]. *Note. Acceptability and feasibility from phase 1 were reported in Teufel et al. (2018).*
Schneider et al. (2020)	NR	**Measure:** interviews **Results:** **System messages** 18 participants thought receiving twice-daily reminders prompted them to take their medicine and to check their peak flow. Three stated that they only use the app when their reminder comes and requested more reminders*. “For me, I like it [text reminders] because it reminds me multiple times, because if it would have just told me once or twice, I probably would not have remembered to do it.”* Participants liked the frequency of the reminders and the ability to schedule them at convenient times. Five participants said that there were too many reminders to check peak flow and found them to be “*annoying”* rather than helpful. Reminders to follow up after a low peak-flow reading was perceived as beneficial to know what was next. *“When it texts ... it keeps me right on track because my phone rings and it tells me to do it [the app, peak flow]”* Two participants thought the advice could be clearer. Three participants could not recall receiving inspirational messages. Participants did think that they were useful and suggested increasing the pool of messages to avoid repetition. **Peak-flow process** Peak-flow entry process was the main way they used the app. 9 participants liked noting their symptoms using the notes feature to write how they were feeling at the time of the reading, mainly to explain low readings. Out of 20 participants, 8 appreciated the advice on how to improve their peak-flow value and when to take the reliever inhaler medication and 9 liked the *“emoji”* faces reflecting how they feel and think in terms of green, yellow, and red readings. Although users could visit their notes and AAP pages outside the peak-flow process, most (*N* = 11) did not use these features. The majority (*N* = 13) valued revisiting or sharing their notes with the physician to explain asthma patterns. Technical problems with entry of peak flow were a barrier. Peak-flow values were stored in a graph to review progress and goals. 18 people) said it was most helpful for them viewing asthma patterns over time. 11 people said the graph’s-coloured zones indicated their peak-flow measures and helped them visualize if their asthma was “*better*” or “*worse.”* For example, *“I think it’s good because you can see if you are getting better or worse or if it is the same.”* **Profile options** 18 participants hardly used the profile page. 11 expressed liked the option to change the wallpaper colour and designs and customize the “emoji” face. Participants liked having the option to customize their profile picture, yet the most specifically older participants (aged 15–17 years), did not use this feature. *“Helps with kids that just want to have their own design unique. And how I designed mine, so when I feel good, I have something that would make me think of my friends, because I like to hang out with them, and then I changed the screen for my home to whatever colour I wanted it to be, and that made it nice. So, you can have your own unique design*.” **Recommendations for improvement** Participants felt it was good and provides the basic needs for them to track their asthma. Overall, participants felt that the app design was outdated and could be improved by adding a larger variety of colours, backgrounds, profile pictures, customisable avatars, font options, and graphics to make it less medical). For example, “Make the profile more interactive like using Avatar. Participants wanted training materials such as videos on the app. Topics suggested: how to use the app, information on strategies to self-manage asthma, or general information about asthma. People wanted the app to incorporate role model (celebrity/athletes) testimonials as well as live video chats with HCPs to train on asthma management activities and communicate asthma information. Better communication was needed: with tech support, the app team, healthcare staff, caregivers, and finally, other teens with asthma. Participants of all ages expressed interest in having a two-way interaction option with the healthcare providers. Social media components were seen by four, mainly older male participants to connect and share how they felt with other teens. “*It would be cool to have kind of like group chat sort of, and kids with the different age group are have it kind of separated in certain age groups and people like younger kids, they can talk about may be how they feel because it’s nice to have somebody who have a same problem as you to say like you can agree on things, kind of like a support group, you can make friends and stuff that have the same problem as you and they understand you*”. Three participants thought it would be extremely helpful to communicate with others who experience similar conditions, while others did not feel strongly about it. Participants wanted fun/entertaining elements for engagement with the DHI. Suggestions included games, avatars, design elements, and incentives for app use. Participants suggested adding visual or sound effects when reaching goals or using the app. Three people mentioned adding a variety of rewards and more engaging tasks that would enhance app use. Avatar accessories, points, stickers, badges, or more app design elements were some rewards suggested for keeping users coming back to the app. For example, *“If you do it for a whole month, you can get a reward ... something that builds up to it”.* **App access** Internet access and login information were barriers to app use. e.g. “*It’s hard sometimes to use it, because when you change the password, you have to enter it a couple times until it submits it. And sometimes when you can’t get through, it has it locked down for like 15 minutes, so then I have to wait 15 minutes and then I log in.”* Limited data and inconsistent Wi-Fi connection presented barriers to use for three of the participants. Others did not want to spend money on data for it. As a solution, these adolescents suggested designing the app to perform offline with occasional data uploads to avoid dependence on an Internet connection. “*Sometimes, when I don’t have Wi-Fi, it is hard for me to document.”* Users frequently forgot their password or did not properly access their unique URL created for the study*. “I don’t like how you need to log back in every time. It’s kind of annoying.”* Participants also recounted times when their peak-flow entry would not be saved, a suggested solution was to add a feature that allows users to save the access information and direct app downloads to avoid URL mistakes.
		

Note: Studies were grouped by study design and ordered in alphabetical order of first author's surname within each grouping. Studies that did not report any data for the secondary outcomes of QoL and user-experiences were removed from this table. Results on the different dimensions of user-experiences were synthesised together under one column. In cases where studies reported multiple quotes to support the same point, XX selected the quote that was most relevant or important.

Symbols: #, number; > , greater than; ≥ , greater than or equal to; = , equals; %, percentage; £ , pound; $, dollar; et al, and others.

Abbreviations: AAP = Asthma Action Plan, App = Application, CARAT = The Control of Allergic Rhinitis and Asthma Test, DHI = Digital Health Intervention, EMM = Electronic Medication Monitoring, HCP = Healthcare Professional, IQR = Inter Quartile Range, MMH = MyMediHealth, NR = Not Reported, *N* = Number, PAQLQ = Paediatric Asthma Quality of Life Questionnaire, QoL = Quality of Life, QOL-CA = Quality of Life Assessment Score for Caregivers of Asthmatic Children, RCT = Randomised Controlled Trial, SAMS = Smartphone Asthma Management System, SD = Standard Deviation, URL = Uniform Resource Locator, vs =

### Appendix 4. Synthesis of positive and negative user-experiences for similar digital features and behavioural strategies

**Table:**

DHI feature or strategy	Positive	Negative
**General—technology**	*Acceptability:* Many users positively appraised the digital format of the interventions (Hashi et al., 2019; Kan et al., 2021) DHI was well-accepted - overall (Johnson et al., 2016; Ramsey et al., 2023) Delivering a DHI via a smartphone app was highly preferred as it is compatible with caregivers' daily phone use (Kan et al., 2021) and aligns with adolescents’ usage and interest in technology (Kan et al., 2021; Nichols et al., 2020) *Engagement:* Users were well-engaged with the DHI at first (Johnson et al., 2016; Wiecha et al., 2015) Users expressing interest or intent to continue to use the DHI after the study period (Cushing et al., 2016; Johnson et al., 2016) Retention & participation rates surpassed a priori feasibility criteria (Ramsey et al., 2023) *Satisfaction & usability:* High levels of user satisfaction with the DHI, including usability & usefulness of features (Johnson et al., 2016; Ramsey et al., 2023; Wiecha et al., 2015) Access to technical support was valued by users (Schneider et al., 2020)	*Engagement & usability:* Engagement declined over time and varied widely (Johnson et al., 2016; Wiecha et al., 2015) Some users experienced technical difficulties, including issues with receiving or responding to notifications/reminders (De Simoni et al., 2021; Hashi et al., 2019; Johnson et al., 2016); pairing or connecting digital devices (e.g. app & sensor) (De Simoni et al., 2021; Kan et al., 2021; Nichols et al., 2020), data entry, upload or syncing (Kan et al., 2021; Nichols et al., 2020; Schneider et al., 2020), forgetting log-in password (Schneider et al., 2020), or incompatibility between mobile carrier and the DHI’s text-messaging service (Johnson et al., 2016) Experiencing technical difficulties hindered engagement (Johnson et al., 2016), ability to adhere to asthma medication (De Simoni et al., 2021) or led to feelings of frustration (Kan et al., 2021) Inadequacy of time, particularly when faced with busy or overwhelming schedules, was a barrier to caregivers and adolescents accessing the DHI or engaging with its features, e.g. uploading videos capturing medicine administration (Johnson et al., 2016; Nichols et al., 2020; Schneider et al., 2020; Wiecha et al., 2015) Prohibited phone use due to school policies or losing phone privileges limits access to the DHI (Cushing et al., 2016; Schneider et al., 2020) *Satisfaction & usability:* The ability to use the DHI offline was suggested (Schneider et al., 2020) Caregivers who are less familiar with smartphone technology, such as grandparents, may find it difficult to use the DHI to support the patients’ asthma management (Kan et al., 2021) The DHI becomes challenging if multiple devices & caregivers are involved (Kan et al., 2021)
**Medication reminders, medication lists and additional reminders for missed medication**	*Engagement*: Many users set medication reminders and/or responded to them (Johnson et al., 2016; Kosse et al., 2019a), including for preventer & reliever medications Many users added their medications (preventer & reliever inhaler) to the medication lists (Johnson et al., 2016) Medication reminders helped prompt users to engage with the DHI (use the app after receiving reminders) (Schneider et al., 2020) *Satisfaction & usability:* Some users considered the medication reminders well-timed (Kan et al., 2021) Some users liked the frequency of medication reminders, the ability to schedule them at convenient times and appreciated the inspirational messages (Scheider et al., 2020) *Perceived benefits*: Many users reported that medication reminders facilitated and improved adherence to asthma medication (Cushing et al., 2016; De Simoni et al., 2021; Kan et al., 2021; Nichols et al., 2020; Schneider et al., 2020), especially adherence to preventer inhaler medication (Kan et al., 2021) Some users reported that medication reminders helped to remind those who forget or busy schedules to administer the asthma medication (Cushing et al., 2016; De Simoni et al., 2021; Kan et al., 2021; Nichols et al., 2020; Schneider et al., 2020) Helped users (that received medication reminders) to establish a routine and improve their confidence/self-efficacy in managing asthma (Johnson et al., 2016; Kan et al., 2021) The reminder alerts facilitated autonomy among children in managing their asthma, transitioning from solely a caregiver responsibility to a shared responsibility (Nichols et al., 2020)	*Engagement*: Some users who entered medications, did not create reminders, e.g. they believed they didn’t need reminders to adhere (Johnson et al., 2016) Some users did not consistently respond to all reminders to complete adherence questions (Kosse et al., 2019a) *Satisfaction & usability:* Adolescents noted that they may miss medication reminders if issued during school hours (Cushing et al., 2016) Some users found the medication reminders annoying (Schneider et al., 2020), or would have preferred less frequent and shorter medication reminders (Cushing et al., 2016) Caregivers desired more app features to involve children to reduce the need for caregiver prompting for medication adherence (Kan et al., 2021) *Perceived benefits*: Caregivers who already established a reliable asthma management routine expressed that medication reminders did not necessarily improve their adherence (Kan et al., 2021)
**Education, skills training, & support from professionals**	*Engagement*: Many adolescents watched the in-app educational short movies (Kosse et al., 2019a) Most adolescents sent or received messages within the pharmacist chat (Kosse et al., 2019a) Text messaging activity was more frequent between patients and the asthma nurse than with the PCP, indicating a preference for communication with asthma nurses (Wiecha et al., 2015) Contents of text messaging activity with HCPs frequently related to encouragement of medication adherence (emotional support) or education/discussion on asthma self-management (informational support) (Wiecha et al., 2015) All users escalated to Step 2 completed the behavioural sessions (Ramsey et al., 2023) *Satisfaction & perceived benefits:* Communication with HCPs as part of the DHI was valued by caregivers to receive (timely & targeted) feedback and improve asthma management (Kan et al., 2021; Nichols et al., 2020; Schneider et al., 2020; Wiecha et al., 2015) Educational content & feedback from HCPs improved user’s (children & caregivers) ability to correctly administer asthma medication, e.g. proper mouth placement on the inhaler (Nichols et al., 2020) Users expressed interest in receiving educational materials in the form of short videos on how to use the app, asthma pathophysiology, and strategies for asthma management (Schneider et al., 2020) Adolescents desired two-way communication with HCPs via live video chats for informational support on asthma and asthma management, and desired periodic educational video clips (Schneider et al. 2020) Users appreciated the convenience and efficiency of digital communication with HCPs (Nichols et al., 2020) Many users were satisfied with the mode of communication with HCPs, i.e. via text or video chat, but generally texting was preferred over phone calls (Kan et al., 2021; Nichols et al., 2020) Some caregivers felt secure and confident with HCP monitoring of patient data—the active presence and concern of HCPs was reassuring and appreciated (Kan et al., 2021; Nichols et al., 2020)	*Engagement & usability:* Some users did not watch any in-app educational short movies or only one (Kosse et al., 2019a) In some cases, the individual that initiated the chat (pharmacist or adolescent) did not receive a chat message back *Satisfaction:* Some caregivers had concerns around HCP monitoring as it made them feel anxious to be watched (Kan et al., 2021; Nichols et al., 2020) *Perceived benefits:* Despite users being informed of the importance of adhering to asthma medication, none of them felt that their attitudes changed on this, indicating that they already understood its importance prior to the DHI (De Simoni et al., 2021) Users warned that the digital communication with HCPs offered by DHIs should not replace face-to-face communication with HCPs (Nichols et al., 2020; Schneider et al., 2020)
**User-monitoring, with access to data**	*Engagement & usability*: Adolescents engaged most frequently with the in-app questionnaire to monitor symptoms (Kosse et al., 2019a) Most adolescents completed the adherence questions at least once (Kosse et al., 2019a) Out of all the DHI features, the peak-flow entry process was perceived to be the main way adolescents used the app (Schneider et al., 2020) Some users were able to successfully log their intake of reliever inhaler (Johnson et al., 2016) Adolescents felt that that the current version of the app provides the basic functionality needed to monitor their asthma (Schneider et al., 2020) Digital diary (web-based) considered easier than hand-writing daily diaries on paper (Hashi et al., 2019) *Satisfaction:* Children liked tracking their medication adherence and the use of graphs to display trends (Cushing et al., 2016) Adolescents liked noting their symptoms, and were pleased with the colour-coded zones as it made it easy to visualise & interpret asthma status (Schneider et al., 2020) Adolescents considered it helpful to have asthma action plan recommendations integrated into data entry for peak flow and for data summaries to be easily compared to personal goals (Schneider et al., 2020) Adolescents liked noting their emotions, including being satisfied with available ‘emoji’ faces (Schneider et al., 2020) Adolescents liked the goal tracker and found it the most helpful feature to view asthma patterns over time (Schneider et al., 2020) Tracking the use of reliever inhaler during asthma exacerbations was useful to caregivers (Kan et al., 2021) Children with asthma were not burdened by the frequency of EMA (Nichols et al., 2020) *Perceived benefits:* User-monitoring improved overall asthma management, including self-management related skills and knowledge, e.g. how peak flow related to asthma status (Cushing et al., 2016; De Simoni et al., 2021; Hashi et al., 2019; Kan et al., 2021; Nichols et al., 2020; Schneider et al., 2020; Wiecha et al., 2015) User-monitoring encouraged a shared responsibility in asthma management between paediatric patients with asthma and their caregivers and promoted patients’ autonomy & engagement in self-management, e.g. independently administer medication or enter data (Kan et al., 2021; Nichols et al., 2020; Schneider et al., 2020) User-monitoring improved caregivers’ confidence to manage their child’s asthma (Kan et al., 2021) User-monitoring & access to visual summaries enabled users to track progress, compare current entries to previous entries and know whether their asthma was getting better or worse, e.g. colour-coded asthma status (Cushing et al., 2016; Schneider et al., 2020) User-monitoring & access to visual summaries helped users to identify patterns of asthma exacerbations and how these connect to environmental triggers, e.g. pollen/weather and heavy exercise (Kan et al., 2021; Schneider et al., 2020) User-monitoring & goal tracker enabled behavioural regulation to meet personal goals, enact recommended actions or avoid exacerbations, e.g. caregivers not sending their child to play outside on high-trigger days (Kan et al., 2021; Schneider et al., 2020) Tracking emotional state helped users contextualise low peak flow readings (Schneider et al., 2020) User-monitoring improved paediatric asthma patients’ health, i.e. fewer asthma symptoms/exacerbations, leading to fewer unplanned hospital visits and less absence (from work/school) (Kan et al., 2021; Nichols et al., 2020) User-monitoring enabled caregivers to remotely verify whether their child administered their asthma medication without need to contact school staff (Kan et al., 2021) Caregivers expressed that the in-app patient record (of user-monitored data) improved & facilitated communication with HCPs at appointments, as it can be revisited or shared to provide a more accurate update than from memory (Kan et al., 2021; Schneider et al., 2020; Wiecha et al., 2015)	*Engagement:* There was a lot of variation between adolescents on engagement with in-app asthma symptom monitoring (Kosse et al., 2019a) Adherence questions appeared 14 times during study period, but on average, adolescents only completed them twice (Kosse et al., 2019a) Users expressed that they may forget or find it difficult to carry the peak-flow meter with them (as it is not built-in or attached to the smartphone), which may limit engagement (Schneider et al., 2020) *Satisfaction*: Users expressed interest in the ability to customize/change the emojis for tracking emotions (Schneider et al., 2020) Some caregivers were dissatisfied that they must enter a password and date for every web-based diary entry (Hashi et al., 2019) One child suggested displaying a number for medication adherence rather than a graph and using points earned rather than actual recorded data to track progress in medication adherence (Cushing et al., 2016) *Perceived benefits*: Some caregivers reported that user-monitoring did not improve their child’s health, as it was already well-managed before or improved with age (Kan et al., 2021)
**Incentives, interactivity & gamification**	*Engagement & usability:* Adolescents were able to make changes to their profile (Schneider et al., 2020) *Satisfaction*: Adolescents felt that the financial incentive value (£1 per preventer inhaler dose), duration (8 weeks) and method of collection (gift cards) was appropriate (De Simoni et al., 2021) User enjoyed the gaming aspects of DHI (Cushing et al., 2016; Schneider et al., 2020) Users appreciated the interactivity of their DHI - such as the use of quizzes (Kan et al., 2021) and in-app customization—such as option to change wallpaper colour, page design, profile picture and emoji faces for data entry (Schneider et al., 2020) *Perceived benefits:* Financial incentives and the point system (within the gaming features) increased adolescent’s motivation to adhere to asthma medication (Cushing et al., 2016; De Simoni et al., 2021) Financial incentives also helped adolescents to remember to self-administer medication and develop a good routine, i.e. they partly work as a reminder (De Simoni et al., 2021)	*Engagement:* Most adolescents only used the profile page minimally (Schneider et al., 2020) Many older adolescents (aged 15 to 17) did not use the profile picture feature (Schneider et al., 2020) *Satisfaction*: Adolescents felt that the app design was outdated with room for improvement on backgrounds, colours, graphics and font options (Schneider et al., 2020) Children & adolescents desired more gamification, e.g. a game where the user’s avatar navigates through a town with different asthma management scenarios (Cushing et al., 2016; Schneider et al., 2020) Adolescents desired more incentives for app use or a variety of rewards (within gamification) to make the DHI more attractive and engaging to adolescents, e.g. unlocking stickers or avatar accessories (Schneider et al., 2020) Users desired more in-app customization and interactivity to make it more personable, engaging, fun and less medical (Cushing et al., 2016; Schneider et al., 2020), e.g. having a customizable avatar for a profile or using visual and sound effects (generally or when goals were reached)
**Support from peers**	*Engagement*: Some users engaged with the peer chat feature (Kosse et al., 2019a; Wiecha et al., 2015) with topics discussed commonly relating to non-medical topics *Perceived benefits:* Some adolescents found the peer chat helpful in connecting and communicating with other adolescents with asthma and share how they are feeling (Schneider et al., 2020) *Other:* The importance of social relations and acceptance was acknowledged by adolescents (Nichols et al., 2020)	*Engagement:* Many users did not use the peer chat feature (Kosse et al., 2019a) *Other*: Users suggested the addition of celebrity role model testimonials on how they manage asthma (Schneider et al., 2020) One adolescent suggested that group chats be created for different age groups of paediatric asthma patients, as a support group (Schneider et al., 2020)

Note: User-experiences were categorised as either positive or negative (including suggestions for improvements) experiences. User-experiences relating to similar digital features and behavioural strategies were grouped together.

Symbols: &, and; et al, and others; £ , pound.

Abbreviations: App = Application, DHI = Digital Health Intervention, EMA = Ecological Momentary Assessment, HCPs = Health Care Professionals, PCP = Primary Care Practitioner.

### Appendix 5. Detailed breakdown of the theoretical mapping of each study

**Table:**

Study	TDF (digital feature/behavioural strategy)	COM-B
***Mixed methods** (N = 2)*		
Cushing et al. (2016)	– Memory, attention, & decision processes; Behavioural regulation; Environmental context & resources; Intentions (users received digital medication reminders that were more frequent for low adherence) – Knowledge; Beliefs about capabilities; Beliefs about consequences; Behavioural regulation; Environmental context & resources; Intentions; Skills (users could monitor medication adherence and access in-app insights based on monitored data) – Social role & identity; Environmental context & resources; Goals; Intentions (users could personalise the app & make a hero avatar, i.e. customisation and gamification features) – Memory, attention, & decision processes; Reinforcement; Environmental context & resources (users received in-app rewards for adherence in the form of gift cards) – Skills; Beliefs about capabilities; Goals; Intentions (users’ ability to adhere to medication was a targeted aim of the DHI, but not further addressed)	**Capability** (psychological & physical) **Opportunity** (physical) **Motivation** (automatic & reflective)
De Simoni et al. (2021)	– Memory, attention, & decision processes; Environmental context & resources; Intentions; Goals (users received digital medication reminders & can program audio reminders) – Knowledge; Beliefs about capabilities; Beliefs about consequences; Behavioural regulation; Intentions; Environmental context & resources; Skills (users could monitor medication adherence and access in-app monitored data) – Skills; Social influences; Goals; Knowledge; Intentions (participants received F2F training on how to use the app system once the EMD was connected) – Knowledge; Social influences; Goals; Intentions; Beliefs about consequences (participants were educated about the importance of taking medication correctly) – Memory, attention, & decision processes; Reinforcement; Environmental context & resources (users received financial incentives in the form of gift cards/vouchers proportionate to adherence rates)	**Capability** (psychological & physical) **Opportunity** (physical & social) **Motivation** (automatic & reflective)
***Quantitative RCTs** (N = 8)*		
Chan et al. (2015)	– Memory, attention, & decision processes; Behavioural regulation; Environmental context & resources; Intentions (users received digital audio-visual medication reminders where the ringtone stops once the correct dose was taken) – Knowledge; Beliefs about capabilities; Beliefs about consequences; Behavioural regulation; Intentions; Environmental context & resources; Social influences; Social role & identity; Skills (users could monitor medication adherence and access insights based on monitored data; caregivers were involved in data collection)	**Capability** (psychological & physical) **Opportunity** (physical & social) **Motivation** (reflective)
Chen et al. (2020)	– Memory, attention, & decision processes; Environmental context & resources; Intentions (users received digital medication reminders from nurses) – Behavioural regulation; Beliefs about capabilities; Intentions; Environmental context & resources; Skills (users could monitor medication adherence) – Knowledge; Skills; Social influences; Goals; Intentions; Social role & identity; Beliefs about consequences (caregivers were trained on medication use & educated on the importance of medication adherence by nurses; caregivers were involved in asthma management) – Social influences; Goals; Behavioural regulation; Intentions; Knowledge; Skills; Environmental context & resources (users received feedback from nurse based on their adherence rate)	**Capability** (psychological & physical) **Opportunity** (physical & social) **Motivation** (reflective)
Gao et al. (2024)	– Memory, attention, & decision processes; Environmental context & resources; Intentions (users received digital medication reminders) – Knowledge; Skills; Social influences; Social role & identity; Goals; Intentions; Environmental context & resources; Behavioural regulation (participants received asthma education based on GINA regimen to improve caregiver supervision of child administration of medication; caregivers were involved in asthma management)	**Capability** (psychological & physical) **Opportunity** (physical & social) **Motivation** (reflective)
Johnson et al. (2016)	– Memory, attention, & decision processes; Behavioural regulation; Environmental context & resources; Intentions (users received digital time-tailored medication reminders, and additional reminders for ‘held’ doses) – Knowledge; Behavioural regulation; Beliefs about capabilities; Beliefs about consequences; Environmental context & resources; Intentions; Goals; Skills (users’ self-efficacy was targeted; users could monitor medication adherence and access visual data summaries for each medication) – Knowledge; Environmental context & resources; Beliefs about capabilities; Intentions; Memory, attention & decision processes; Goals (participants could create and print a structured medication list and attach a dosing schedule to each medication; users were informed of when a refill is needed before a particular date based on user-monitored data) – Skills; Social influences; Knowledge; Intentions; Environmental context & resources (users received instructions and a demonstration video on how to use and test the web app) – Social influences; Goals; Knowledge; Social role & identity; Intentions; Environmental context & resources (users had access to 24-hour support and help desk to troubleshoot and document problems with users who have been inactive; caregivers were involved in asthma management) – Memory, attention, & decision processes; Reinforcement; Environmental context & resources (participants were financially compensated for enrolment and study completion)	**Capability** (psychological & physical) **Opportunity** (physical & social) **Motivation** (automatic & reflective)
Kosse et al. (2019a)	– Memory, attention, & decision processes; Environmental context & resources; Intentions (users received digital medication alarms to prevent forgetting, and push notification reminders to complete symptom questionnaire) – Behavioural regulation; Knowledge; Beliefs about consequences; Beliefs about capabilities; Environmental context & resources; Intentions; Skills (users could monitor medication adherence and symptoms and could access their data in-app) – Knowledge; Skills; Environmental context & resources; Intentions (users had access to in-app educational short movies on asthma topics including inhaler technique) – Social influences; Beliefs about capabilities; Goals; Intentions; Knowledge; Environmental context & resources; Behavioural regulation; Belief about consequences (users received textual feedback from pharmacists on symptom scores to improve disease control; pharmacists could send movie suggestions based on the patients’ needs and monitored data, i.e. goal setting) – Social influences; Knowledge; Optimism (users had access to pharmacist chat where they received education & motivation) – Social influences; Social role & identity; Environmental context & resources (users had access to peer chats to share experiences and discuss asthma-related topics with other patient users)	**Capability** (psychological & physical) **Opportunity** (physical & social) **Motivation** (reflective)
Kosse et al. (2019b)	– Memory, attention, & decision processes; Environmental context & resources; Intentions (users received digital medication alarms to prevent forgetting) – Knowledge; Behavioural regulation; Beliefs about consequences; Beliefs about capabilities; Environmental context & resources; Intentions; Skills (users could monitor medication adherence and symptoms and could access their data in-app) – Social influences; Professional role & identity; Beliefs about capabilities; Goals; Behavioural regulation; Knowledge; Intentions; Environmental context & resources; Optimism; Beliefs about consequences (users received textual feedback from pharmacists on symptom scores to improve disease control; pharmacists could send movie suggestions, i.e. goal setting; pharmacist chat to educate and motivate users) – Knowledge; Environmental context & resources; Intentions; Social influences; Optimism (users had access to short educational and motivational movies on asthma related topics in-app) – Social influences; Social role & identity; Environmental context & resources; Goals; Intentions (users had access to peer chat to contact other patient users)	**Capability** (psychological & physical) **Opportunity** (physical & social) **Motivation** (reflective)
Vasbinder et al. (2016)	– Memory, attention, & decision processes; Behavioural regulation; Environmental context & resources; Intentions (users received digital time-tailored medication reminders and additional reminders for missed/unrecorded medication use) – Behavioural regulation; Beliefs about capabilities; Environmental context & resources; Intentions; Skills (users could monitor medication adherence) – Social influences (caregivers were involved in asthma management)	**Capability** (psychological & physical) **Opportunity** (physical & social) **Motivation** (reflective)
Wiecha et al. (2015)	– Memory, attention, & decision processes; Behavioural regulation; Environmental context & resources; Intentions (users received digital alerts for dangerous values in peak flow) – Knowledge; Beliefs about consequences; Behavioural regulation; Beliefs about capabilities; Intentions; Social influences; Environmental context & resources; Social role & identity; Skills (users could monitor medication adherence, symptoms, absence from school, and emergency room visits; users could view graphs based on user-monitored data; users received education and could complete educational quizzes; website could be accessed by patient or caregiver) – Social influences; Social role & identity; Goals; Knowledge; Behavioural regulation; Intentions; Environmental context & resources; Beliefs about consequences (users received personalised support from asthma specialists with recommendations based on user-monitored web-based data; users had access to discussion boards with peers & PCPs; users could view other users’ personal web pages; PCP had access to the patients' asthma status; caregivers were involved in asthma management) – Knowledge; Skills; Social influences; Intentions; Social or professional role & identity; Environmental context & resources (patients, caregivers and physicians were trained on the use of BB website; patients were instructed on how to use PIKO; patients could access a library of information, asthma word searches, and complete educational quizzes/puzzles) – Memory, attention, & decision processes; Reinforcement; Environmental context & resources (users earned points for feature use that were redeemable for gift cards; users received financial incentives for website use and survey completion)	**Capability** (psychological & physical) **Opportunity** (physical & social) **Motivation** (automatic & reflective)
***Quantitative descriptive** (N = 2)*		
Hashi et al. (2019)	– Memory, attention, & decision processes; Environmental context & resources; Intentions (users received digital reminders to complete daily diary entries) – Behavioural regulation; Beliefs about capabilities; Environmental context & resources; Intentions; Skills (users could monitor medication adherence and symptoms) – Optimism; Social influences; Emotions; Intentions; Environmental context & resources (users received emotional support from researchers in the form of encouraging comments, but not informational support) – Social influences; Social role & identity; Emotion; Intentions (caregivers were involved in asthma management; parenting stress and family functioning were assessed)	**Capability** (psychological & physical) **Opportunity** (physical & social) **Motivation** (automatic & reflective)
Ramsey et al. (2023)	– Memory, attention & decision processes; Environmental context & resources; Intentions (users received digital medication reminders in Step 1; users received push notifications for weekly mobile spirometry throughout) – Behavioural regulation; Beliefs about capabilities; Environmental context & resources; Intentions; Skills (users could monitor medication adherence in Step 1) – Knowledge; Skills; Behavioural regulation; Social influences; Goals; Beliefs about capabilities; Beliefs about consequences; Intentions; Environmental context & resources (users had access to tailored telehealth behavioural intervention sessions in Step 2 which included self-monitoring strategies and guided problem-solving training, i.e. goal setting; users had access to adherence feedback in Step 2) – Knowledge; Skills; Social influences; Environmental context & resources; Goals; Intentions (participants were trained on the use of a mobile spirometer using ATS guidelines)	**Capability** (psychological & physical) **Opportunity** (physical & social) **Motivation** (reflective)
***Qualitative** (N = 3)*		
Kan et al. (2021)	– Memory, attention, & decision processes; Behavioural regulation; Environmental context & resources; Goals; Intentions (users could set digital reminders at their preferred times and were notified by push notifications for missed medications) – Behavioural regulation; Knowledge; Beliefs about consequences; Beliefs about capabilities; Environmental context & resources; Intentions; Skills (users could monitor medication adherence and environmental allergens; users had access to daily reports based on user-monitored data) – Knowledge; Social influences; Goals; Intentions; Professional role & identity; Environmental context & resources; Behavioural regulation; Beliefs about consequences (clinical staff were alerted by email and via the web portal on use of preventer and reliever inhaler and would call users to triage how to improve adherence to preventer inhaler or understand the cause of increased usage of reliever inhaler) – Emotions; Social influences; Social role & identity; Intentions (compatibility with lifestyle and emotional impact were assessed; caregivers were involved in asthma management)	**Capability** (psychological & physical) **Opportunity** (physical & social) **Motivation** (automatic & reflective)
Nichols et al. (2020)	– Memory, attention, & decision processes; Environmental context & resources; Goals; Intentions (users received digital reminders to encourage completion of the EMA questions, video capture, and participation in the telehealth visit) – Behavioural regulation; Emotion; Beliefs about consequences; Knowledge; Beliefs about capabilities; Intentions; Environmental context & resources (users could monitor their medication adherence in real-time which reduces recall bias, users also monitored symptoms, and emotional state; users could view their monitored data in-app) – Knowledge; Skills; Social influences; Optimism; Environmental context & resources; Goals; Intentions; Professional role & identity; Behavioural regulation; Beliefs about capabilities (users could interact with provider via (i) asynchronous video capture of inhaler use technique and (ii) synchronous direct-to-participant smartphone-based telehealth visit; users received personalised feedback and motivational interviewing from an asthma educator, the DHI was modified based on participant feedback) – Knowledge; Skills; Beliefs about capabilities; Goals; Intentions; Behavioural regulation (users’ competence, including the ability to recognise symptoms and use the inhaler were a targeted aim of the DHI) – Social influences; Goals (caregiver involvement in asthma management was encouraged, but not required)	**Capability** (psychological & physical) **Opportunity** (physical & social) **Motivation** (automatic & reflective)
Schneider et al. (2020)	– Memory, attention, & decision processes; Behavioural regulation; Environmental context & resources; Intentions; Goals; Social influences (users received digital reminders to use the app for monitoring and after low readings; reminders could be adjusted by users according to their preferences—i.e. customisation features) – Behavioural regulation; Knowledge; Beliefs about consequences; Goals; Beliefs about capabilities; Intentions; Emotions; Environmental context & resources; Skills (users could monitor disease patterns, symptoms, triggers, feelings, and peak-flow readings; users could access monitored data visually in the form of graphs and in comparison to their goals in-app goal tracking feature) – Social influences; Goals; Knowledge; Skills; Intentions; Behavioural regulation; Optimism; Beliefs about consequences (users received personalised feedback and inspirational messages, based on their reports and individual asthma action plan, guiding them on how to manage their asthma) – Knowledge; Skills; Social influences; Environmental context & resources; Goals; Intentions (participants were trained in the use of the peak-flow meter) – Goals; Intentions; Environmental context & resources (users were able to make changes to their profile, i.e. customisation features)	**Capability** (psychological & physical) **Opportunity** (physical & social) **Motivation** (automatic & reflective)

Note: Studies were grouped by study design and ordered in alphabetical order of first author's surname within each grouping.

In the TDF column, the mapped domains for each study were listed and the applicable digital feature(s) or behavioural strategy(ies) for groupings of domains were enclosed within brackets. In the COM-B column, the applicable components were bolded, and applicable sub-components were enclosed within brackets.

Symbols: = , equals to;/, or; &, and; et al, and others.

Abbreviations: App = Application, ATS = American Thoracic Society, BB = Bostonbreathes, COM-B = Capability, Opportunity, Motivation, Behaviour, EMA = Ecological Momentary Assessment, EMD = Electronic Monitoring Device, EMR = Electronic Medical Records, F2F = Face To Face, GINA = Global Initiaice for Asthma, RCT = Randomised Controlled Trial, TDF = Theoretical Domains Framework, PCP = Primary Care Provider.

### Appendix 6. Visual summary of theoretical mapping across studies and total frequencies per study and theoretical construct

**Table:**

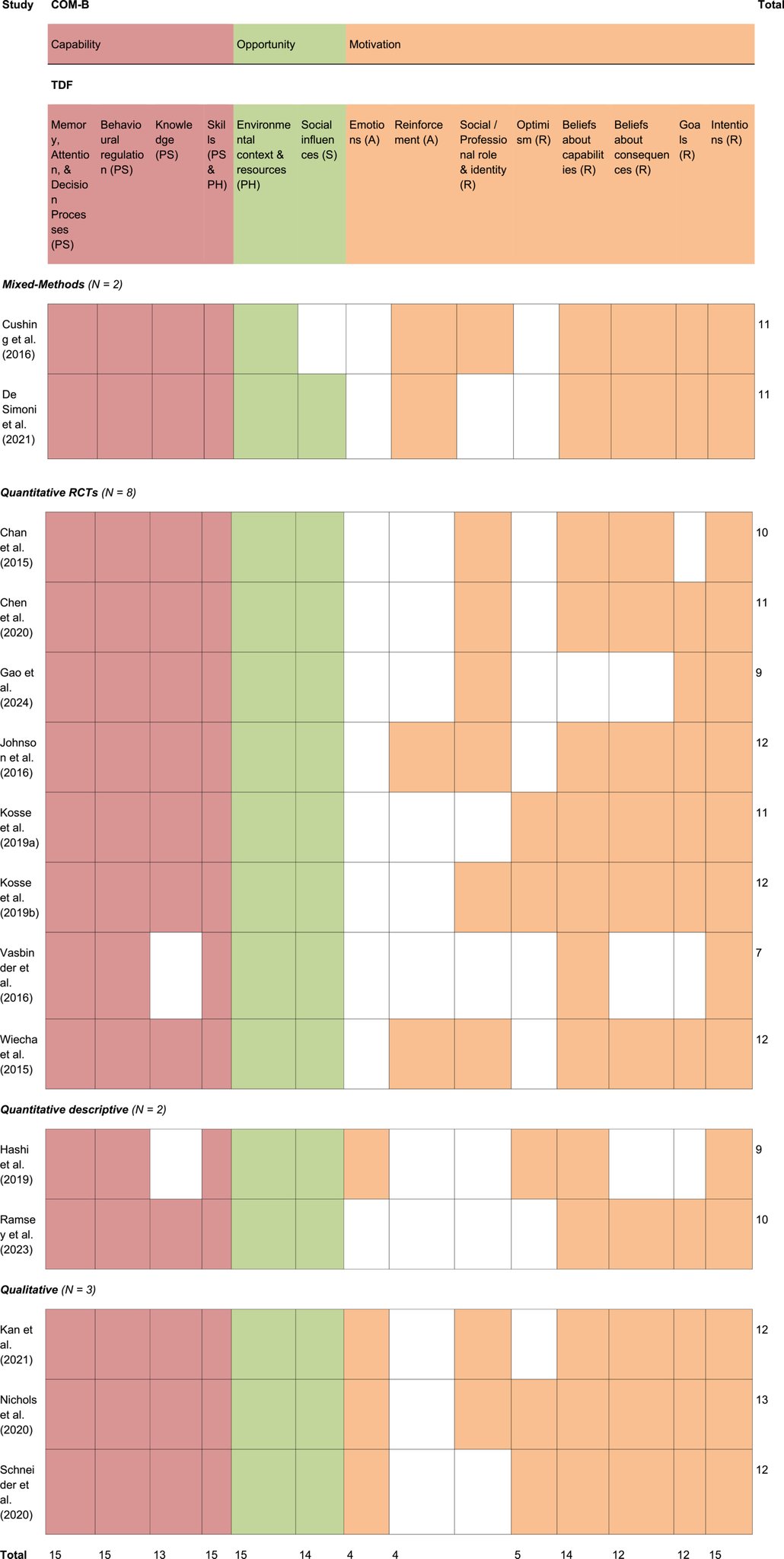

Note: Studies were grouped by study design and ordered in alphabetical order of first author's surname within each grouping.

All studies mapped onto all three COM-B components. Total in the final column refers to the total number of applicable TDF domains per study. Total in the final row refers to the total number of applicable studies per TDF domain.

Symbols: et al, and others; = , equals to.

Abbreviations: A = Automatic (motivation), COM-B = Capability, Opportunity, Motivation, Behaviour, *N* = Number, PH = Physical (capability or opportunity), PS = Psychological (capability), R = Reflective (motivation), RCT = Randomised Controlled Trial, S = Social (opportunity), TDF = Theoretical Domains Framework
