# Supplementary material for: Are digital health interventions acceptable and effective at improving medication adherence in paediatric patients with asthma? A mixed-mode systematic review, narrative synthesis, and behavioural analysis
Source: Health Psychol Behav Med. 2026 Nov 11;14(1):2745675. doi: 10.1080/21642850.2026.2745675 (PMC13761040; doi:10.1080/21642850.2026.2745675)
Supplement: Supplementary file 1 [file RHPB_A_2745675_SM1639.docx]

**Supplemental material 1**

*Completed PRISMA checklist*

| **Section & topic** | **Item #** | **Item description** | **Location of item reporting** |
| --- | --- | --- | --- |
| **TITLE** | | |  |
| Title | 1 | Identify the report as a systematic review. | 1 |
| **ABSTRACT** | | |  |
| Abstract | 2 | See the PRISMA 2020 for Abstracts checklist. | 2 |
| **INTRODUCTION** | | |  |
| Rationale | 3 | Describe the rationale for the review in the context of existing knowledge. | 3 |
| Objectives | 4 | Provide an explicit statement of the objective(s) or question(s) the review addresses. | 6-7 |
| **METHODS** | | |  |
| Eligibility criteria | 5 | Specify the inclusion and exclusion criteria for the review and how studies were grouped for the syntheses. | 7 |
| Information sources | 6 | Specify all databases, registers, websites, organisations, reference lists and other sources searched or consulted to identify studies. Specify the date when each source was last searched or consulted. | 8 |
| Search strategy | 7 | Present the full search strategies for all databases, registers, and websites, including any filters and limits used. | 8 |
| Selection process | 8 | Specify the methods used to decide whether a study met the inclusion criteria of the review, including how many reviewers screened each record and each report retrieved, whether they worked independently, and if applicable, details of automation tools used in the process. | 8 |
| Data collection process | 9 | Specify the methods used to collect data from reports, including how many reviewers collected data from each report, whether they worked independently, any processes for obtaining or confirming data from study investigators, and if applicable, details of automation tools used in the process. | 8 |
| Data items | 10a | List and define all outcomes for which data were sought. Specify whether all results that were compatible with each outcome domain in each study were sought (e.g. for all measures, time points, analyses), and if not, the methods used to decide which results to collect. | 8 |
|  | 10b | List and define all other variables for which data were sought (e.g. participant and intervention characteristics, funding sources). Describe any assumptions made about any missing or unclear information. |  |
| Study risk of bias assessment | 11 | Specify the methods used to assess risk of bias in the included studies, including details of the tool(s) used, how many reviewers assessed each study and whether they worked independently, and if applicable, details of automation tools used in the process. | 9 |
| Effect measures | 12 | Specify for each outcome the effect measure(s) (e.g. risk ratio, mean difference) used in the synthesis or presentation of results. | 9  9 |
| Synthesis methods | 13a | Describe the processes used to decide which studies were eligible for each synthesis (e.g. tabulating the study intervention characteristics and comparing against the planned groups for each synthesis (item #5)). |  |
|  | 13b | Describe any methods required to prepare the data for presentation or synthesis, such as handling of missing summary statistics, or data conversions. |  |
|  | 13c | Describe any methods used to tabulate or visually display results of individual studies and syntheses. |  |
|  | 13d | Describe any methods used to synthesize results and provide a rationale for the choice(s). If meta-analysis was performed, describe the model(s), method(s) to identify the presence and extent of statistical heterogeneity, and software package(s) used. |  |
|  | 13e | Describe any methods used to explore possible causes of heterogeneity among study results (e.g. subgroup analysis, meta-regression). |  |
|  | 13f | Describe any sensitivity analyses conducted to assess robustness of the synthesized results. |  |
| Reporting bias assessment | 14 | Describe any methods used to assess risk of bias due to missing results in a synthesis (arising from reporting biases). | N/A |
| Certainty assessment | 15 | Describe any methods used to assess certainty (or confidence) in the body of evidence for an outcome. | 9 |
| **RESULTS** | | |  |
| Study selection | 16a | Describe the results of the search and selection process, from the number of records identified in the search to the number of studies included in the review, ideally using a flow diagram. | 10-11 |
|  | 16b | Cite studies that might appear to meet the inclusion criteria, but which were excluded, and explain why they were excluded. |  |
| Study characteristics | 17 | Cite each included study and present its characteristics. | 12 |
| Risk of bias in studies | 18 | Present assessments of risk of bias for each included study. | 34 |
| Results of individual studies | 19 | For all outcomes, present, for each study: (a) summary statistics for each group (where appropriate) and (b) an effect estimate and its precision (e.g. confidence/credible interval), ideally using structured tables or plots. | 14 |
| Results of syntheses | 20a | For each synthesis, briefly summarise the characteristics and risk of bias among contributing studies. | 34 |
|  | 20b | Present results of all statistical syntheses conducted. If meta-analysis was done, present for each the summary estimate and its precision (e.g. confidence/credible interval) and measures of statistical heterogeneity. If comparing groups, describe the direction of the effect. |  |
|  | 20c | Present results of all investigations of possible causes of heterogeneity among study results. |  |
|  | 20d | Present results of all sensitivity analyses conducted to assess the robustness of the synthesized results. |  |
| Reporting biases | 21 | Present assessments of risk of bias due to missing results (arising from reporting biases) for each synthesis assessed. | NR |
| Certainty of evidence | 22 | Present assessments of certainty (or confidence) in the body of evidence for each outcome assessed. | 34 |
| **DISCUSSION** | | |  |
| Discussion | 23a | Provide a general interpretation of the results in the context of other evidence. | 49 |
|  | 23b | Discuss any limitations of the evidence included in the review. | 56 |
|  | 23c | Discuss any limitations of the review processes used. | 56 |
|  | 23d | Discuss implications of the results for practice, policy, and future research. | 50 |
| **OTHER INFORMATION** | | |  |
| Registration and protocol | 24a | Provide registration information for the review, including register name and registration number, or state that the review was not registered. | 7 |
|  | 24b | Indicate where the review protocol can be accessed, or state that a protocol was not prepared. |  |
|  | 24c | Describe and explain any amendments to information provided at registration or in the protocol. |  |
| Support | 25 | Describe sources of financial or non-financial support for the review, and the role of the funders or sponsors in the review. | 60 |
| Competing interests | 26 | Declare any competing interests of review authors. |  |
| Availability of data, code, and other materials | 27 | Report which of the following are publicly available and where they can be found: template data collection forms; data extracted from included studies; data used for all analyses; analytic code; any other materials used in the review. |  |

*Note. The table template was sourced from Page et al. (2020).*

*Symbols & abbreviations: # = number, PRISMA = Preferred Reporting Items for Systematic Reviews and Meta-analyses.*

**Supplemental material 2**

*Completed PRISMA checklist for the abstract*

| **Section & topic** | **Item #** | **Item description** | **Reported (Yes/No)** |
| --- | --- | --- | --- |
| **TITLE** | | |  |
| Title | 1 | Identify the report as a systematic review. | Yes |
| **BACKGROUND** | | |  |
| Objectives | 2 | Provide an explicit statement of the main objective(s) or question(s) the review addresses. | Yes |
| **METHODS** | | |  |
| Eligibility criteria | 3 | Specify the inclusion and exclusion criteria for the review. | Yes |
| Information sources | 4 | Specify the information sources (e.g. databases, registers) used to identify studies and the date when each was last searched. | No (sources)  Yes (date of last search) |
| Risk of bias | 5 | Specify the methods used to assess risk of bias in the included studies. | No |
| Synthesis of results | 6 | Specify the methods used to present and synthesise results. | Yes |
| **RESULTS** | | |  |
| Included studies | 7 | Give the total number of included studies and participants and summarise relevant characteristics of studies. | Yes |
| Synthesis of results | 8 | Present results for main outcomes, preferably indicating the number of included studies and participants for each. If meta-analysis was done, report the summary estimate and confidence/credible interval. If comparing groups, indicate the direction of the effect (i.e. which group is favoured). | Yes |
| **DISCUSSION** | | |  |
| Limitations of evidence | 9 | Provide a brief summary of the limitations of the evidence included in the review (e.g. study risk of bias, inconsistency, and imprecision). | Yes |
| Interpretation | 10 | Provide a general interpretation of the results and important implications. | Yes |
| **OTHER** | | |  |
| Funding | 11 | Specify the primary source of funding for the review. | N/A |
| Registration | 12 | Provide the register name and registration number. | Yes |

*Note. The table template was sourced from Page et al. (2020).*

*Symbols & abbreviations: # = number, PRISMA = Preferred Reporting Items for Systematic Reviews and Meta-analyses.*

**Supplemental material 3**

*SWiM reporting table*

| **SWiM reporting item** | **Item description** | **Page in manuscript where item is reported** |
| --- | --- | --- |
| **Methods** | | |
| 1. Grouping studies for synthesis | 1a) Provide a description of, and rationale for, the groups used in the synthesis (eg, groupings of populations, interventions, outcomes, study design)   1b) Detail and provide rationale for any changes made subsequent to the protocol in the groups used in the synthesis | 9 |
| 2. Describe the standardised metric and transformation methods used | Describe the standardised metric for each outcome. Explain why the metric(s) was chosen and describe any methods used to transform the intervention effects, as reported in the study, to the standardised metric, citing any methodological guidance consulted | 8 |
| 3. Describe the synthesis methods | Describe and justify the methods used to synthesise the effects for each outcome when it was not possible to undertake a meta-analysis of effect estimates | 9 |
| 4. Criteria used to prioritise results for summary and synthesis | Where applicable, provide the criteria used, with supporting justification, to select the particular studies, or a particular study, for the main synthesis or to draw conclusions from the synthesis (e.g., based on study design, risk of bias assessments, directness in relation to the review question) | 9 |
| 5. Investigation of heterogeneity in reported effects | State the method(s) used to examine heterogeneity in reported effects when it was not possible to undertake a meta-analysis of effect estimates and its extensions to investigate heterogeneity | 9 |
| 6. Certainty of evidence | Describe the methods used to assess the certainty of the synthesis findings | 9 |
| 7. Data presentation methods | 7a) Describe the graphical and tabular methods used to present the effects (e.g., tables, forest plots, harvest plots)   7b) Specify key study characteristics (e.g., study design, risk of bias) used to order the studies, in the text and any tables or graphs, clearly referencing the studies included | 9 |
| **Results** | | |
| 8. Reporting results | For each comparison and outcome, provide a description of the synthesised findings and the certainty of the findings. Describe the result in language that is consistent with the question the synthesis addresses, and indicate which studies contribute to the synthesis | 34 |
| **Discussion** | | |
| 9. Limitations of the synthesis | Report the limitations of the synthesis methods used and/or the groupings used in the synthesis and how these affect the conclusions that can be drawn in relation to the original review question | 56 |

*Note. The table template was sourced from Campbell et al. (2020).*

*Symbols & abbreviations: SWIM = Synthesis without meta-analysis.*

**Supplemental material 4**

*Search strategy per database*

| **Database** | **Search strategy** |
| --- | --- |
| **CINAHL** | S1 TI newborn OR AB newborn, S2 TI infant OR AB infant, S3 TI child OR AB child, S4 TI junior OR AB junior, S5 TI juvenile OR AB juvenile, S6 TI pediatric OR AB pediatric, S7 TI paediatric OR AB paediatric, S8 TI adolescen* OR AB adolescen*, S9 TI teen* OR AB teen*, S10 TI "young people" OR AB "young people", S11 TI "young adults" OR AB "young adults", S12 TI youth OR AB youth, S13 MH infant, S14 MH child, S15 MW adolescent    S1 OR S2 OR S3 OR S4 OR S5 OR S6 OR S7 OR S8 OR S9 OR S10 OR S11 OR S12 OR S13 OR S14 OR S15    S17 TI asthma OR AB asthma, S18 MH asthma    S17 OR S18    S20 TI telehealth OR AB telehealth, S21 TI telemedicine OR AB telemedicine, S22 TI telecommunications OR AB telecommunications, S23 TI telecare OR AB telecare, S24 TI telepharmacy OR AB telepharmacy, S25 TI telemonitoring OR AB telemonitoring, S26 TI teleconference OR AB teleconference, S27 TI app OR AB app, S28 TI apps OR AB apps, S29 TI mobile OR AB mobile, S30 TI "mobile health" OR AB "mobile health", S31 TI smartphone OR AB smartphone, S32 TI phone OR AB phone, S33 TI telephone OR AB telephone, S34 TI cellphone OR AB cellphone, S35 TI "mobile app" OR AB "mobile app", S36 TI "cellphone app" OR AB "cellphone app", S37 TI "smartphone app" OR AB "smartphone app", S38 TI "smart phone app" OR AB "smart phone app", S39 TI "portable app" OR AB "portable app", S40 TI wearables OR AB wearables, S41 TI "text messag*” OR AB "text messag*”, S42 TI SMS OR AB SMS, S43 TI notification OR AB notification, S44 TI reminder OR AB reminder, S45 TI online OR AB online, S46 TI internet OR AB internet, S47 TI wireless OR AB wireless, S48 TI "online intervention" OR AB "online intervention", S49 TI software OR AB software, S50 TI "computer based" OR AB "computer based", S51 TI "computer assisted" OR AB "computer assisted", S52 TI "remote intervention" OR AB "remote intervention", S53 TI "remote consultation" OR AB "remote consultation", S54 TI "digital health" OR AB "digital health", S55 TI mhealth OR AB mhealth, S56 TI m-health OR AB m-health, S57 TI ehealth OR AB ehealth, S58 TI e-health OR AB e-health, S59 TI emedicine OR AB emedicine, S60 TI webbased OR AB webbased, S61 TI "web based" OR AB "web based", S62 TI webpage OR AB webpage, S63 TI website OR AB website. S64 TI gamification OR AB gamification. S65 TI "game based" OR AB "game based", S66 TI "virtual reality" OR AB "virtual reality", S67 TI digital OR AB digital, S68 TI "digital technology" OR AB "digital technology", S69 TI laptop OR AB laptop, S70 TI computer OR AB computer, S71 TI microsoft OR AB microsoft, S72 TI MAC OR AB MAC, S73 TI IPAD OR AB IPAD, S74 TI tablet OR AB tablet, S75 TI virtual OR AB virtual, S76 TI electronic OR AB electronic, S77 TI "electronic health" OR AB "electronic health", S78 MH telemedicine, S79 MH mobile applications, S80 MH smartphone, S81 MH software, S82 MH internet, S83 MH internet-based intervention, S84 MW decision making, computer-assisted, S85 MH digital technology    S20 OR S21 OR S22 OR S23 OR S24 OR S25 OR S26 OR S27 OR S28 OR S29 OR S30 OR S31 OR S32 OR S33 OR S34 OR S35 OR S36 OR S37 OR S38 OR S39 OR S40 OR S41 OR S42 OR S43 OR S44 OR S45 OR S46 OR S47 OR S48 OR S49 OR S50 OR S51 OR S52 OR S53 OR S54 OR S55 OR S56 OR S57 OR S58 OR S59 OR S60 OR S61 OR S62 OR S63 OR S64 OR S65 OR S66 OR S67 OR S68 OR S69 OR S70 OR S71 OR S72 OR S73 OR S74 OR S75 OR S76 OR S77 OR S78 OR S79 OR S80 OR S81 OR S82 OR S83 OR S84 OR S85    S87 TI "medication adherence" OR AB "medication adherence", S88 TI "medication adherance" OR AB "medication adherance", S89 TI "medication compliance" OR AB "medication compliance", S90 TI "medication persistence" OR AB "medication persistence", S91 TI "medication non adherence” OR AB "medication non adherence”, S92 TI "medication nonadherence" OR AB "medication nonadherence", S93 TI "medication non adherance" OR AB "medication non adherance", S94 TI "medication nonadherance" OR AB "medication nonadherance", S95 TI "medication non compliance" OR AB "medication non compliance", S96 V, S97 TI "medication non persistence" OR AB "medication non persistence", S98 TI "medication nonpersistence" OR AB "medication nonpersistence", S99 TI "medication use" OR AB "medication use", S100 TI "medication control" OR AB "medication control", S101 TI "patient compliance" OR AB "patient compliance", S102 TI Nebulizer OR AB Nebulizer, S103 TI "medicine adherence" OR AB "medicine adherence", S104 TI "medicine adherance" OR AB "medicine adherance", S105 TI "medicine compliance" OR AB "medicine compliance", S106 TI "medicine persistence" OR AB "medicine persistence", S107 TI "medicine non adherence" OR AB "medicine non adherence", S108 TI "medicine nonadherence" OR AB "medicine nonadherence", S109 TI "medicine non adherance" OR AB "medicine non adherance", S110 TI "medicine nonadherance" OR AB "medicine nonadherance", S111 TI "medicine non compliance" OR AB "medicine non compliance", S112 TI "medicine noncompliance" OR AB "medicine noncompliance", S113 TI "medicine non persistence" OR AB "medicine non persistence", S114 TI "medicine nonpersistence" OR AB "medicine nonpersistence", S115 TI "medicine use" OR AB "medicine use", S116 TI "medicine control" OR AB "medicine control", S117 TI "drug adherence" OR AB "drug adherence", S118 TI "drug adherance" OR AB "drug adherance", S119 TI "drug compliance" OR AB "drug compliance", S120 TI "drug persistence" OR AB "drug persistence", S121 TI "drug non adherence" OR AB "drug non adherence", S122 TI "drug nonadherence" OR AB "drug nonadherence", S123 TI "drug non adherance" OR AB "drug non adherance", S124 TI "drug nonadherance" OR AB "drug nonadherance", S125 TI "drug non compliance" OR AB "drug non compliance", S126 TI "drug noncompliance" OR AB "drug noncompliance", S127 TI "drug non persistence" OR AB "drug non persistence", S128 TI "drug nonpersistence" OR AB "drug nonpersistence", S129 TI "drug use" OR AB "drug use", S130 TI "drug control" OR AB "drug control", S131 TI "treatment adherence" OR AB "treatment adherence", S132 TI "treatment adherance" OR AB "treatment adherance", S133 TI "treatment compliance" OR AB "treatment compliance", S134 TI "treatment persistence" OR AB "treatment persistence", S135 TI “treatment non adherence" OR AB “treatment non adherence", S136 TI "treatment nonadherence" OR AB "treatment nonadherence", S137 TI "treatment non adherance" OR AB "treatment non adherance", S138 TI "treatment nonadherance" OR AB "treatment nonadherance", S139 TI "treatment non compliance" OR AB "treatment non compliance", S140 TI "treatment noncompliance" OR AB "treatment noncompliance", S141 TI "treatment non persistence" OR AB "treatment non persistence", S142 TI "treatment nonpersistence" OR AB "treatment nonpersistence", S143 TI "treatment use" OR AB "treatment use", S144 TI "treatment control” OR AB "treatment control”, S145 MW adherence, S146 MH patient compliance, S147 MW (nebulizers and vaporizers)    S87 OR S88 OR S89 OR S90 OR S91 OR S92 OR S93 OR S94 OR S95 OR S96 OR S97 OR S98 OR S99 OR S100 OR S101 OR S102 OR S103 OR S104 OR S105 OR S106 OR S107 OR S108 OR S109 OR S110 OR S111 OR S112 OR S113 OR S114 OR S115 OR S116 OR S117 OR S118 OR S119 OR S120 OR S121 OR S122 OR S123 OR S124 OR S125 OR S126 OR S127 OR S128 OR S129 OR S130 OR S131 OR S132 OR S133 OR S134 OR S135 OR S136 OR S137 OR S138 OR S139 OR S140 OR S141 OR S142 OR S143 OR S144 OR S145 OR S146 OR S147    S16 AND S19 AND S86 AND S148 |
| **Cochrane Library** | #1 (newborn OR infant OR child OR junior OR juvenile OR pediatric OR paediatric OR adolescen* OR teen* OR "young people" OR "young adults" OR youth):ti,ab,kw (Word variations have been searched)  #2 MeSH descriptor: [Infant] explode all trees  #3 MeSH descriptor: [Child] explode all trees  #4 MeSH descriptor: [Adolescent] explode all trees  #5 #1 OR #2 OR #3 OR #4  #6 (asthma):ti,ab,kw (Word variations have been searched)  #7 MeSH descriptor: [Asthma] explode all trees  #8 #6 OR #7  #9 (telehealth OR telemedicine OR telecommunications OR telecare OR telepharmacy OR telemonitoring OR teleconference OR app OR apps OR mobile OR "mobile health" OR smartphone OR phone OR telephone OR cellphone OR "mobile app" OR "cellphone app" OR "smartphone app" OR "smart phone app" OR portable app" OR wearables OR "text messag* OR SMS OR notification OR reminder OR online OR internet OR wireless OR "online intervention" OR software OR "computer based" OR "computer assisted" OR "remote intervention" OR "remote consultation" OR "digital health" OR mhealth OR m-health OR ehealth OR e-health OR emedicine OR webbased OR "web based" OR webpage OR website OR gamification OR "game based" OR "virtual reality" OR digital OR "digital technology" OR laptop OR computer OR microsoft OR MAC or IPAD or tablet OR virtual OR electronic OR "electronic health"):ti,ab,kw (Word variations have been searched)  #10 MeSH descriptor: [Telemedicine] explode all trees  #11 MeSH descriptor: [Mobile Applications] explode all trees  #12 MeSH descriptor: [Smartphone] explode all trees  #13 MeSH descriptor: [Software] explode all trees  #14 MeSH descriptor: [Internet] explode all trees  #15 MeSH descriptor: [Internet-Based Intervention] explode all trees  #16 MeSH descriptor: [Decision Making, Computer-Assisted] explode all trees  #17 MeSH descriptor: [Digital Technology] explode all trees  #18 #9 OR #10 OR #11 OR #12 OR #13 OR #14 OR #15 OR #16 OR #17  #19 ((“Medication adherence” OR “Medication adherance” OR “Medication compliance” OR “Medication persistence” OR “Medication non adherence” OR “Medication nonadherence” OR “medication non adherance” OR “Medication nonadherance” OR “Medication non compliance” OR “Medication noncompliance” OR “Medication non persistence” OR “Medication nonpersistence” OR “Medication use” OR “Medication control” OR “Medicine adherence” OR “Medicine adherance” OR “Medicine compliance” OR “Medicine persistence” OR “Medicine non adherence” OR “Medicine nonadherence” OR “Medicine non adherance” OR “Medicine nonadherance” OR “Medicine non compliance” OR “Medicine noncompliance” OR “Medicine non persistence” OR “Medicine nonpersistence” OR “Medicine use” OR “Medicine control” OR “Drug adherence” OR “Drug adherance” OR “Drug compliance” OR “Drug persistence” OR “Drug non adherence” OR “Drug nonadherence” OR “Drug non adherance” OR “Drug nonadherance” OR “Drug non compliance” OR “Drug noncompliance” OR “Drug non persistence” OR “Drug nonpersistence” OR “Drug use” OR “Drug control” OR “Treatment adherence” OR “Treatment adherance” OR “Treatment compliance” OR “Treatment persistence” OR “Treatment non adherence” OR “Treatment nonadherence” OR “Treatment non adherance” OR “Treatment nonadherance” OR “Treatment non compliance” OR “Treatment noncompliance” OR “Treatment non persistence” OR “Treatment nonpersistence” OR “treatment control” OR “treatment use” OR “Patient compliance” OR Nebulizer)):ti,ab,kw (Word variations have been searched)  #20 MeSH descriptor: [Medication Adherence] explode all trees  #21 MeSH descriptor: [Treatment Adherence and Compliance] explode all trees  #22 MeSH descriptor: [Patient Compliance] explode all trees  #23 MeSH descriptor: [Nebulizers and Vaporizers] explode all trees  #24 #19 OR #20 OR #21 OR #22 OR #23  #25 #5 AND #8 AND #18 AND #24 |
| **Medline** | 1 newborn.m_titl., 2 infant.m_titl., 3 child.m_titl., 4 junior.m_titl., 5 juvenile.m_titl., 6 pediatric.m_titl., 7 paediatric.m_titl., 8 "adolescen*".m_titl., 9 "teen*".m_titl., 10 "young people".m_titl., 11 "young adults".m_titl., 12 youth.m_titl, 13 Infant/, 14 Child/, 15 Adolescent/    1 or 2 or 3 or 4 or 5 or 6 or 7 or 8 or 9 or 10 or 11 or 12 or 13 or 14 or 15    17 asthma.m_titl., 18 Asthma/    17 or 18    20 telehealth.m_titl., 21 telemedicine.m_titl., 22 telecommunications.m_titl., 23 telecare.m_titl., 24 telepharmacy.m_titl., 25 telemonitoring.m_titl., 26 teleconference.m_titl., 27 app.m_titl., 28 apps.m_titl., 29 mobile.m_titl., 30 "mobile health".m_titl., 31 smartphone.m_titl., 32 phone.m_titl., 33 telephone.m_titl., 34 cellphone.m_titl., 35 "mobile app".m_titl., 36 "cellphone app".m_titl., 37 "smartphone app".m_titl., 38 "smart phone app".m_titl., 39 "portable app".m_titl., 40 wearables.m_titl., 41 "text messag* ".m_titl., 42 SMS.m_titl., 43 notification.m_titl., 44 reminder.m_titl., 45 online.m_titl., 46 internet.m_titl., 47 wireless.m_titl., 48 "online intervention".m_titl., 49 software.m_titl., 50 "computer based".m_titl., 51 "computer assisted".m_titl., 52 "remote intervention".m_titl., 53 "remote consultation".m_titl., 54 "digital health".m_titl., 55 mhealth.m_titl., 56 m-health.m_titl., 57 ehealth.m_titl., 58 e-health.m_titl., 59 emedicine.m_titl., 60 webbased.m_titl., 61 "web based".m_titl., 62 webpage.m_titl., 63 website.m_titl., 64 gamification.m_titl., 65 "game based".m_titl., 66 "virtual reality".m_titl., 67 digital.m_titl., 68 "digital technology".m_titl., 69 laptop.m_titl., 70 computer.m_titl., 71 microsoft.m_titl., 72 MAC.m_titl., 73 IPAD.m_titl., 74 tablet.m_titl., 75 virtual.m_titl., 76 electronic.m_titl., 77 "electronic health".m_titl., 78 Telemedicine/, 79 Mobile Applications/, 80 Smartphone/, 81 Software/, 82 Internet/, 83 Internet-Based Intervention/, 84 Decision Making, Computer-Assisted/, 85 Digital Technology/    20 or 21 or 22 or 23 or 24 or 25 or 26 or 27 or 28 or 29 or 30 or 31 or 32 or 33 or 34 or 35 or 36 or 37 or 38 or 39 or 40 or 41 or 42 or 43 or 44 or 45 or 46 or 47 or 48 or 49 or 50 or 51 or 52 or 53 or 54 or 55 or 56 or 57 or 58 or 59 or 60 or 61 or 62 or 63 or 64 or 65 or 66 or 67 or 68 or 69 or 70 or 71 or 72 or 73 or 74 or 75 or 76 or 77 or 78 or 79 or 80 or 81 or 82 or 83 or 84 or 85    87 "medication adherence".m_titl., 88 "medication adherance".m_titl., 89 "medication compliance".m_titl., 90 "medication persistence".m_titl., 91 "medication non adherence".m_titl., 92 "medication nonadherence".m_titl., 93 "medication non adherance".m_titl., 94 "medication nonadherance".m_titl., 95 "medication non compliance".m_titl., 96 "medication noncompliance".m_titl., 97 "medication non persistence".m_titl., 98 "medication nonpersistence".m_titl., 99 "medication nonpersistence".m_titl., 100 "medication nonpersistence".m_titl., 101 "patient compliance".m_titl., 102 Nebulizer.m_titl., 103 "medicine adherence".m_titl., 104 "medicine adherance".m_titl., 105 "medicine compliance".m_titl., 106 "medicine persistence".m_titl., 107 "medicine non adherence".m_titl., 108 "medicine nonadherence".m_titl., 109 "medicine non adherance".m_titl., 110 "medicine nonadherance".m_titl., 111 "medicine non compliance".m_titl., 112 "medicine noncompliance".m_titl., 113 "medicine non persistence".m_titl., 114 "medicine nonpersistence".m_titl., 115 "medicine use".m_titl., 116 "medicine control".m_titl., 117 "drug adherence".m_titl., 118 "drug adherance".m_titl., 119 "drug compliance".m_titl., 120 "drug persistence".m_titl., 121 "drug non adherence".m_titl., 122 "drug nonadherence".m_titl., 123 "drug non adherance".m_titl., 124 "drug nonadherance".m_titl., 125 "drug non compliance".m_titl., 126 "drug noncompliance".m_titl., 127 "drug non persistence".m_titl., 128 "drug nonpersistence".m_titl., 129 "drug use".m_titl., 130 "drug control".m_titl., 131 "treatment adherence".m_titl., 132 "treatment adherance".m_titl., 133 "treatment compliance".m_titl., 134 "treatment persistence".m_titl., 135 "treatment non adherence".m_titl., 136 "treatment nonadherence".m_titl., 137 "treatment non adherance".m_titl., 138 "treatment nonadherance".m_titl., 139 "treatment non compliance".m_titl., 140 "treatment noncompliance".m_titl., 141 "treatment non persistence".m_titl., 142 "treatment nonpersistence".m_titl., 143 "treatment use".m_titl., 144 "treatment control".m_titl., 145 Medication Adherence/, 146 (treatment adherence and compliance).mp., 147 "Treatment Adherence and Compliance"/, 148 Patient Compliance/, 149 (nebulizers and vapourizers).mp., 150 "Nebulizers and Vaporizers"/    87 or 88 or 89 or 90 or 91 or 92 or 93 or 94 or 95 or 96 or 97 or 98 or 99 or 100 or 101 or 102 or 103 or 104 or 105 or 106 or 107 or 108 or 109 or 110 or 111 or 112 or 113 or 114 or 115 or 116 or 117 or 118 or 119 or 120 or 121 or 122 or 123 or 124 or 125 or 126 or 127 or 128 or 129 or 130 or 131 or 132 or 133 or 134 or 135 or 136 or 137 or 138 or 139 or 140 or 141 or 142 or 143 or 144 or 145 or 146 or 147 or 148 or 149 or 150    16 and 19 and 86 and 151 |
| **PsychINFO** | S1 TI newborn OR AB newborn, S2 TI infant OR AB infant, S3 TI child OR AB child, S4 TI junior OR AB junior, S5 TI juvenile OR AB juvenile, S6 TI pediatric OR AB pediatric, S7 TI paediatric OR AB paediatric, S8 TI adolescen* OR AB adolescen*, S9 TI teen* OR AB teen*, S10 TI "young adults" OR AB "young adults", S11 TI "young people" OR AB "young people, S12 TI youth OR AB youth, S13 MJ infant, S14 MJ child, S15 MJ adolescents    S1 OR S2 OR S3 OR S4 OR S5 OR S6 OR S7 OR S8 OR S9 OR S10 OR S11 OR S12 OR S13 OR S14 OR S15    S17 TI asthma OR AB asthma, S18 MJ asthma    S17 OR S18    S20 TI telehealth OR AB telehealth, S21 TI telemedicine OR AB telemedicine, S22 TI telecommunications OR AB telecommunications, S23 TI telecare OR AB telecare, S24 TI telepharmacy OR AB telepharmacy, S25 TI telemonitoring OR AB telemonitoring, S26 TI teleconference OR AB teleconference, S27 TI app OR AB app, S28 TI apps OR AB apps, S29 TI mobile OR AB mobile, S30 TI "mobile health" OR AB "mobile health", S31 TI smartphone OR AB smartphone, S32 TI phone OR AB phone, S33 TI telephone OR AB telephone, S34 TI cellphone OR AB cellphone, S35 TI "mobile app" OR AB "mobile app", S36 TI "cellphone app" OR AB "cellphone app", S37 TI "smartphone app" OR AB "smartphone app", S38 TI "smart phone app" OR AB "smart phone app", S39 TI "portable app" OR AB "portable app", S40 TI wearables OR AB wearables, S41 TI "text messag*” OR AB "text messag*”, S42 TI SMS OR AB SMS, S43 TI notification OR AB notification, S44 TI reminder OR AB reminder, S45 TI online OR AB online, S46 TI internet OR AB internet, S47 TI wireless OR AB wireless, S48 TI "online intervention" OR AB "online intervention", S49 TI software OR AB software, S50 TI "computer based" OR AB "computer based", S51 TI "computer assisted" OR AB "computer assisted", S52 TI "remote intervention" OR AB "remote intervention", S53 TI "remote consultation" OR AB "remote consultation", S54 TI "digital health" OR AB "digital health", S55 TI mhealth OR AB mhealth, S56 TI m-health OR AB m-health, S57 TI ehealth OR AB ehealth, S58 TI e-health OR AB e-health, S59 TI emedicine OR AB emedicine, S60 TI webbased OR AB webbased, S61 TI "web based" OR AB "web based", S62 TI webpage OR AB webpage, S63 TI website OR AB website. S64 TI gamification OR AB gamification. S65 TI "game based" OR AB "game based", S66 TI "virtual reality" OR AB "virtual reality", S67 TI digital OR AB digital, S68 TI "digital technology" OR AB "digital technology", S69 TI laptop OR AB laptop, S70 TI computer OR AB computer, S71 TI microsoft OR AB microsoft, S72 TI MAC OR AB MAC, S73 TI IPAD OR AB IPAD, S74 TI tablet OR AB tablet, S75 TI virtual OR AB virtual, S76 TI electronic OR AB electronic, S77 TI "electronic health" OR AB "electronic health", S78 MJ telemedicine, S79 MJ mobile applications, S80 MJ smartphone, S81 MJ software, S82 MJ internet, S83 MA internet-based intervention, S84 MA decision making, computer-assisted, S85 MJ digital technology    S20 OR S21 OR S22 OR S23 OR S24 OR S25 OR S26 OR S27 OR S28 OR S29 OR S30 OR S31 OR S32 OR S33 OR S34 OR S35 OR S36 OR S37 OR S38 OR S39 OR S40 OR S41 OR S42 OR S43 OR S44 OR S45 OR S46 OR S47 OR S48 OR S49 OR S50 OR S51 OR S52 OR S53 OR S54 OR S55 OR S56 OR S57 OR S58 OR S59 OR S60 OR S61 OR S62 OR S63 OR S64 OR S65 OR S66 OR S67 OR S68 OR S69 OR S70 OR S71 OR S72 OR S73 OR S74 OR S75 OR S76 OR S77 OR S78 OR S79 OR S80 OR S81 OR S82 OR S83 OR S84 OR S85    S87 TI "medication adherence" OR AB "medication adherence", S88 TI "medication adherance" OR AB "medication adherance", S89 TI "medication compliance" OR AB "medication compliance", S90 TI "medication persistence" OR AB "medication persistence", S91 TI "medication non adherence” OR AB "medication non adherence”, S92 TI "medication nonadherence" OR AB "medication nonadherence", S93 TI "medication non adherance" OR AB "medication non adherance", S94 TI "medication nonadherance" OR AB "medication nonadherance", S95 TI "medication non compliance" OR AB "medication non compliance", S96 V, S97 TI "medication non persistence" OR AB "medication non persistence", S98 TI "medication nonpersistence" OR AB "medication nonpersistence", S99 TI "medication use" OR AB "medication use", S100 TI "medication control" OR AB "medication control", S101 TI "patient compliance" OR AB "patient compliance", S102 TI Nebulizer OR AB Nebulizer, S103 TI "medicine adherence" OR AB "medicine adherence", S104 TI "medicine adherance" OR AB "medicine adherance", S105 TI "medicine compliance" OR AB "medicine compliance", S106 TI "medicine persistence" OR AB "medicine persistence", S107 TI "medicine non adherence" OR AB "medicine non adherence", S108 TI "medicine nonadherence" OR AB "medicine nonadherence", S109 TI "medicine non adherance" OR AB "medicine non adherance", S110 TI "medicine nonadherance" OR AB "medicine nonadherance", S111 TI "medicine non compliance" OR AB "medicine non compliance", S112 TI "medicine noncompliance" OR AB "medicine noncompliance", S113 TI "medicine non persistence" OR AB "medicine non persistence", S114 TI "medicine nonpersistence" OR AB "medicine nonpersistence", S115 TI "medicine use" OR AB "medicine use", S116 TI "medicine control" OR AB "medicine control", S117 TI "drug adherence" OR AB "drug adherence", S118 TI "drug adherance" OR AB "drug adherance", S119 TI "drug compliance" OR AB "drug compliance", S120 TI "drug persistence" OR AB "drug persistence", S121 TI "drug non adherence" OR AB "drug non adherence", S122 TI "drug nonadherence" OR AB "drug nonadherence", S123 TI "drug non adherance" OR AB "drug non adherance", S124 TI "drug nonadherance" OR AB "drug nonadherance", S125 TI "drug non compliance" OR AB "drug non compliance", S126 TI "drug noncompliance" OR AB "drug noncompliance", S127 TI "drug non persistence" OR AB "drug non persistence", S128 TI "drug nonpersistence" OR AB "drug nonpersistence", S129 TI "drug use" OR AB "drug use", S130 TI "drug control" OR AB "drug control", S131 TI "treatment adherence" OR AB "treatment adherence", S132 TI "treatment adherance" OR AB "treatment adherance", S133 TI "treatment compliance" OR AB "treatment compliance", S134 TI "treatment persistence" OR AB "treatment persistence", S135 TI “treatment non adherence" OR AB “treatment non adherence", S136 TI "treatment nonadherence" OR AB "treatment nonadherence", S137 TI "treatment non adherance" OR AB "treatment non adherance", S138 TI "treatment nonadherance" OR AB "treatment nonadherance", S139 TI "treatment non compliance" OR AB "treatment non compliance", S140 TI "treatment noncompliance" OR AB "treatment noncompliance", S141 TI "treatment non persistence" OR AB "treatment non persistence", S142 TI "treatment nonpersistence" OR AB "treatment nonpersistence", S143 TI "treatment use" OR AB "treatment use", S144 TI "treatment control” OR AB "treatment control”, S145 MA adherence, S146 MA treatment adherence and compliance, S147 treatment compliance, S148 MA patient compliance, S149 MA (nebulizers and vaporizers)    S87 OR S88 OR S89 OR S90 OR S91 OR S92 OR S93 OR S94 OR S95 OR S96 OR S97 OR S98 OR S99 OR S100 OR S101 OR S102 OR S103 OR S104 OR S105 OR S106 OR S107 OR S108 OR S109 OR S110 OR S111 OR S112 OR S113 OR S114 OR S115 OR S116 OR S117 OR S118 OR S119 OR S120 OR S121 OR S122 OR S123 OR S124 OR S125 OR S126 OR S127 OR S128 OR S129 OR S130 OR S131 OR S132 OR S133 OR S134 OR S135 OR S136 OR S137 OR S138 OR S139 OR S140 OR S141 OR S142 OR S143 OR S144 OR S145 OR S146 OR S147 OR S148 OR S149    S16 AND S19 AND S86 AND S150 |
| **Pubmed** | "(((((((((((((((((newborn[Title/Abstract]) OR (infant[Title/Abstract])) OR (child[Title/Abstract])) OR (junior[Title/Abstract])) OR (juvenile[Title/Abstract])) OR (pediatric[Title/Abstract])) OR (paediatric[Title/Abstract])) OR (adolescen*[Title/Abstract])) OR (teen*[Title/Abstract])) OR (""young people""[Title/Abstract])) OR (""young adults""[Title/Abstract])) OR (youth[Title/Abstract])) OR (infant[MeSH Terms])) OR (child[MeSH Terms])) OR (adolescent[MeSH Terms])) AND ((asthma[Title/Abstract]) OR (asthma[MeSH Terms]))) AND ((((((((((((((((((((((((((((((((((((((((((((((((((((((((((((((((((telehealth[Title/Abstract]) OR (telemedicine[Title/Abstract])) OR (telecommunications[Title/Abstract])) OR (telecare[Title/Abstract])) OR (telepharmacy[Title/Abstract])) OR (telemonitoring[Title/Abstract])) OR (teleconference[Title/Abstract])) OR (app[Title/Abstract])) OR (apps[Title/Abstract])) OR (mobile[Title/Abstract])) OR (""mobile health""[Title/Abstract])) OR (smartphone[Title/Abstract])) OR (phone[Title/Abstract])) OR (telephone[Title/Abstract])) OR (cellphone[Title/Abstract])) OR (""cellphone app""[Title/Abstract])) OR (""mobile app""[Title/Abstract])) OR (""smartphone app""[Title/Abstract])) OR (""smart phone app""[Title/Abstract])) OR (""portable app""[Title/Abstract])) OR (wearables[Title/Abstract])) OR (""text messag*""[Title/Abstract])) OR (SMS[Title/Abstract])) OR (notification[Title/Abstract])) OR (reminder[Title/Abstract])) OR (online[Title/Abstract])) OR (internet[Title/Abstract])) OR (wireless[Title/Abstract])) OR (""online intervention""[Title/Abstract])) OR (software[Title/Abstract])) OR (""computer based""[Title/Abstract])) OR (""computer assisted""[Title/Abstract])) OR (""remote intervention""[Title/Abstract])) OR (""remote consultation""[Title/Abstract])) OR (""digital health""[Title/Abstract])) OR (mhealth[Title/Abstract])) OR (m-health[Title/Abstract])) OR (ehealth[Title/Abstract])) OR (e-health[Title/Abstract])) OR (emedicine[Title/Abstract])) OR (webbased[Title/Abstract])) OR (""web based""[Title/Abstract])) OR (webpage[Title/Abstract])) OR (website[Title/Abstract])) OR (gamification[Title/Abstract])) OR (""game based""[Title/Abstract])) OR (""virtual reality""[Title/Abstract])) OR (digital[Title/Abstract])) OR (""digital technology""[Title/Abstract])) OR (laptop[Title/Abstract])) OR (computer[Title/Abstract])) OR (microsoft[Title/Abstract])) OR (MAC[Title/Abstract])) OR (IPAD[Title/Abstract])) OR (tablet[Title/Abstract])) OR (virtual[Title/Abstract])) OR (electronic[Title/Abstract])) OR (""electronic health""[Title/Abstract])) OR (telemedicine[MeSH Terms])) OR (mobile applications[MeSH Terms])) OR (smartphones[MeSH Terms])) OR (computer software[MeSH Terms])) OR (internet[MeSH Terms])) OR (internet based interventions[MeSH Terms])) OR (computer assisted decision making[MeSH Terms])) OR (digital technology[MeSH Terms]))) AND (((((((((((((((((((((((((((((((((((((((((((((((((((((((((((((((""medication adherence""[Title/Abstract]) OR (""medication adherance""[Title/Abstract])) OR (""medication compliance""[Title/Abstract])) OR (""medication persistence""[Title/Abstract])) OR (""medication non adherence""[Title/Abstract])) OR (""medication nonadherence""[Title/Abstract])) OR (""medication non adherance""[Title/Abstract])) OR (""medication nonadherance""[Title/Abstract])) OR (""medication non compliance""[Title/Abstract])) OR (""medication noncompliance""[Title/Abstract])) OR (""medication non persistence""[Title/Abstract])) OR (""medication nonpersistence""[Title/Abstract])) OR (""medication use""[Title/Abstract])) OR (""medication control""[Title/Abstract])) OR (""patient compliance""[Title/Abstract])) OR (Nebulizer[Title/Abstract])) OR (""medicine adherence""[Title/Abstract])) OR (""medicine adherance""[Title/Abstract])) OR (""medicine compliance""[Title/Abstract])) OR (""medicine persistence""[Title/Abstract])) OR (""medicine non adherence""[Title/Abstract])) OR (""medicine nonadherence""[Title/Abstract])) OR (""medicine non adherance""[Title/Abstract])) OR (""medicine nonadherance""[Title/Abstract])) OR (""medicine non compliance""[Title/Abstract])) OR (""medicine noncompliance""[Title/Abstract])) OR (""medicine non persistence""[Title/Abstract])) OR (""medicine nonpersistence""[Title/Abstract])) OR (""medicine use""[Title/Abstract])) OR (""medicine control""[Title/Abstract])) OR (""drug adherence""[Title/Abstract])) OR (""drug adherance""[Title/Abstract])) OR (""drug compliance""[Title/Abstract])) OR (""drug persistence""[Title/Abstract])) OR (""drug non adherence""[Title/Abstract])) OR (""drug nonadherence""[Title/Abstract])) OR (""drug non adherance""[Title/Abstract])) OR (""drug nonadherance""[Title/Abstract])) OR (""drug non compliance""[Title/Abstract])) OR (""drug noncompliance""[Title/Abstract])) OR (""drug non persistence""[Title/Abstract])) OR (""drug nonpersistence""[Title/Abstract])) OR (""drug use""[Title/Abstract])) OR (""drug control""[Title/Abstract])) OR (""treatment adherence""[Title/Abstract])) OR (""treatment adherance""[Title/Abstract])) OR (""treatment compliance""[Title/Abstract])) OR (""treatment persistence""[Title/Abstract])) OR (""treatment non adherence""[Title/Abstract])) OR (""treatment nonadherence""[Title/Abstract])) OR (""treatment non adherance""[Title/Abstract])) OR (""treatment nonadherance""[Title/Abstract])) OR (""treatment non compliance""[Title/Abstract])) OR (""treatment noncompliance""[Title/Abstract])) OR (""treatment non persistence""[Title/Abstract])) OR (""treatment nonpersistence""[Title/Abstract])) OR (""treatment use""[Title/Abstract])) OR (""treatment control[Title/Abstract])) OR (medication adherence[MeSH Terms])) OR (treatment adherence and compliance[MeSH Terms])) OR (treatment compliance[MeSH Terms])) OR (patient compliance[MeSH Terms])) OR (nebulizers and vaporizers[MeSH Terms  le/Abstract] |
| **Scopus** | TITLE-ABS-KEY ( newborn ) OR TITLE-ABS-KEY ( infant ) OR TITLE-ABS-KEY ( child* ) OR TITLE-ABS-KEY ( junior ) OR TITLE-ABS-KEY ( juvenile ) OR TITLE-ABS-KEY ( paediatric ) OR TITLE-ABS-KEY ( pediatric ) OR TITLE-ABS-KEY ( adolescen* ) OR TITLE-ABS-KEY ( teen* ) OR TITLE-ABS-KEY ( "young people" ) OR TITLE-ABS-KEY ( "young adults" ) OR TITLE-ABS-KEY ( youth ) AND TITLE-ABS-KEY ( asthma ) AND TITLE-ABS-KEY ( telemedicine ) OR TITLE-ABS-KEY ( telehealth ) OR TITLE-ABS-KEY ( telecommunications ) OR TITLE-ABS-KEY ( telecare ) OR TITLE-ABS-KEY ( telepharmacy ) OR TITLE-ABS-KEY ( telemonitoring ) OR TITLE-ABS-KEY ( teleconference ) OR TITLE-ABS-KEY ( app ) OR TITLE-ABS-KEY ( apps ) OR TITLE-ABS-KEY ( mobile ) OR TITLE-ABS-KEY ( "mobile health" ) OR TITLE-ABS-KEY ( smartphone ) OR TITLE-ABS-KEY ( phone ) OR TITLE-ABS-KEY ( telephone ) OR TITLE-ABS-KEY ( cellphone ) OR TITLE-ABS-KEY ( "mobile app" ) OR TITLE-ABS-KEY ( "cellphone app" ) OR TITLE-ABS-KEY ( "smartphone app" ) OR TITLE-ABS-KEY ( "smart phone app" ) OR TITLE-ABS-KEY ( "portable app" ) OR TITLE-ABS-KEY ( wearables ) OR TITLE-ABS-KEY ( "text messag*" ) OR TITLE-ABS-KEY ( sms ) OR TITLE-ABS-KEY ( notification ) OR TITLE-ABS-KEY ( reminder ) OR TITLE-ABS-KEY ( online ) OR TITLE-ABS-KEY ( internet ) OR TITLE-ABS-KEY ( wireless ) OR TITLE-ABS-KEY ( "online intervention" ) OR TITLE-ABS-KEY ( software ) OR TITLE-ABS-KEY ( "computer based" ) OR TITLE-ABS-KEY ( "computer assisted" ) OR TITLE-ABS-KEY ( "remote intervention" ) OR TITLE-ABS-KEY ( "remote consultation" ) OR TITLE-ABS-KEY ( "digital health" ) OR TITLE-ABS-KEY ( mhealth ) OR TITLE-ABS-KEY ( m-health ) OR TITLE-ABS-KEY ( ehealth ) OR TITLE-ABS-KEY ( e-health ) OR TITLE-ABS-KEY ( emedicine ) OR TITLE-ABS-KEY ( e-medicine ) OR TITLE-ABS-KEY ( webbased ) OR TITLE-ABS-KEY ( "web based" ) OR TITLE-ABS-KEY ( "web page" ) OR TITLE-ABS-KEY ( webpage ) OR TITLE-ABS-KEY ( website ) OR TITLE-ABS-KEY ( web ) OR TITLE-ABS-KEY ( gamification ) OR TITLE-ABS-KEY ( "game based" ) OR TITLE-ABS-KEY ( "virtual reality" ) OR TITLE-ABS-KEY ( digital ) OR TITLE-ABS-KEY ( "digital technology" ) OR TITLE-ABS-KEY ( laptop ) OR TITLE-ABS-KEY ( computer ) OR TITLE-ABS-KEY ( microsoft ) OR TITLE-ABS-KEY ( mac ) OR TITLE-ABS-KEY ( ipad ) OR TITLE-ABS-KEY ( tablet ) OR TITLE-ABS-KEY ( virtual ) OR TITLE-ABS-KEY ( electronic ) OR TITLE-ABS-KEY ( "electronic health" ) AND TITLE-ABS-KEY ( "Medication adherence" ) OR TITLE-ABS-KEY ( "Medication adherance" ) OR TITLE-ABS-KEY ( "Medication compliance" ) OR TITLE-ABS-KEY ( "Medication persistence" ) OR TITLE-ABS-KEY ( "Medication non adherence" ) OR TITLE-ABS-KEY ( "Medication nonadherence" ) OR TITLE-ABS-KEY ( "medication non adherance" ) OR TITLE-ABS-KEY ( "Medication nonadherance" ) OR TITLE-ABS-KEY ( "Medication non compliance" ) OR TITLE-ABS-KEY ( "Medication noncompliance" ) OR TITLE-ABS-KEY ( "Medication non persistence" ) OR TITLE-ABS-KEY ( "Medication nonpersistence" ) OR TITLE-ABS-KEY ( "Medication use" ) OR TITLE-ABS-KEY ( "Medication control" ) OR TITLE-ABS-KEY ( "Medicine adherence" ) OR TITLE-ABS-KEY ( "Medicine adherance" ) OR TITLE-ABS-KEY ( "Medicine compliance" ) OR TITLE-ABS-KEY ( "Medicine persistence" ) OR TITLE-ABS-KEY ( "Medicine non adherence" ) OR TITLE-ABS-KEY ( "Medicine nonadherence" ) OR TITLE-ABS-KEY ( "Medicine non adherance" ) OR TITLE-ABS-KEY ( "Medicine nonadherance" ) OR TITLE-ABS-KEY ( "Medicine non compliance" ) OR TITLE-ABS-KEY ( "Medicine noncompliance" ) OR TITLE-ABS-KEY ( "Medicine non persistence" ) OR TITLE-ABS-KEY ( "Medicine nonpersistence" ) OR TITLE-ABS-KEY ( "Medicine use" ) OR TITLE-ABS-KEY ( "Medicine control" ) OR TITLE-ABS-KEY ( "Drug adherence" ) OR TITLE-ABS-KEY ( "Drug adherance" ) OR TITLE-ABS-KEY ( "Drug compliance" ) OR TITLE-ABS-KEY ( "Drug persistence" ) OR TITLE-ABS-KEY ( "Drug non adherence" ) OR TITLE-ABS-KEY ( "Drug nonadherence" ) OR TITLE-ABS-KEY ( "Drug non adherance" ) OR TITLE-ABS-KEY ( "Drug nonadherance" ) OR TITLE-ABS-KEY ( "Drug non compliance" ) OR TITLE-ABS-KEY ( "Drug noncompliance" ) OR TITLE-ABS-KEY ( "Drug non persistence" ) OR TITLE-ABS-KEY ( "Drug nonpersistence" ) OR TITLE-ABS-KEY ( "Drug use" ) OR TITLE-ABS-KEY ( "Drug control" ) OR TITLE-ABS-KEY ( "Treatment adherence" ) OR TITLE-ABS-KEY ( "Treatment adherance" ) OR TITLE-ABS-KEY ( "Treatment compliance" ) OR TITLE-ABS-KEY ( "Treatment persistence" ) OR TITLE-ABS-KEY ( "Treatment non adherence" ) OR TITLE-ABS-KEY ( "Treatment nonadherence" ) OR TITLE-ABS-KEY ( "Treatment non adherance" ) OR TITLE-ABS-KEY ( "Treatment nonadherance" ) OR TITLE-ABS-KEY ( "Treatment non compliance" ) OR TITLE-ABS-KEY ( "Treatment noncompliance" ) OR TITLE-ABS-KEY ( "Treatment non persistence" ) OR TITLE-ABS-KEY ( "Treatment nonpersistence" ) OR TITLE-ABS-KEY ( "treatment control" ) OR TITLE-ABS-KEY ( "treatment use" ) OR TITLE-ABS-KEY ( "Patient compliance" ) OR TITLE-ABS-KEY ( nebulizer ) |
| **Web of Science** | 1: (((((((((((((((((((((((TI=(Newborn)) OR AB=(newborn)) OR TI=(infant)) OR AB=(infant)) OR AB=(child)) OR TI=(child)) OR TI=(junior)) OR AB=(junior)) OR TI=(juvenile)) OR AB=(juvenile)) OR TI=(pediatric)) OR AB=(pediatric)) OR TI=(paediatric)) OR AB=(paediatric)) OR TI=(adolescen*)) OR AB=(adolescen*)) OR TI=(teen*)) OR AB=(teen*)) OR TI=("young people")) OR AB=("young people")) OR TI=("young adults")) OR AB=("young adults")) OR TI=(youth)) OR AB=(youth) and Preprint Citation Index (Exclude – Database)    2: (TI=(asthma)) OR AB=(asthma) and Preprint Citation Index (Exclude – Database)    3: ((((((((((((((((((((((((((((((((((((((((((((((((((((((((((((((((((((((((((((((((((((((((((((((((((((((((((((((((((TI=(telehealth)) OR TI=(telemedicine)) OR TI=(telecommunications)) OR TI=(telecare)) OR TI=(telepharmacy)) OR TI=(telemonitoring )) OR TI=(teleconference)) OR TI=(app)) OR TI=(apps)) OR TI=(mobile)) OR TI=("mobile health")) OR TI=(smartphone)) OR TI=(phone)) OR TI=(telephone)) OR TI=(cellphone)) OR TI=("mobile app")) OR TI=("cellphone app")) OR TI=("smartphone app")) OR TI=("smart phone app")) OR TI=("portable app")) OR TI=(wearables)) OR TI=("text messag*")) OR TI=(SMS)) OR TI=(notification)) OR TI=(reminder)) OR TI=(online)) OR TI=(internet)) OR TI=(wireless)) OR TI=("online intervention")) OR TI=(software)) OR TI=("computer based")) OR TI=("computer assisted")) OR TI=("remote intervention")) OR TI=("digital health")) OR TI=(mhealth)) OR TI=(m-health)) OR TI=(ehealth)) OR TI=(e-health)) OR TI=(emedicine)) OR TI=(webbased)) OR TI=("web based")) OR TI=(webpage)) OR TI=(website)) OR TI=(gamification)) OR TI=("game based")) OR TI=("virtual reality")) OR TI=(digital)) OR TI=("digital technology")) OR TI=(laptop)) OR TI=(computer)) OR TI=(microsoft)) OR TI=(MAC)) OR TI=(IPAD)) OR TI=(tablet)) OR TI=(virtual)) OR TI=(electronic)) OR TI=("electronic health")) OR AB=(telehealth)) OR AB=(telemedicine)) OR AB=(telecommunications)) OR AB=(telecare)) OR AB=(telepharmacy)) OR AB=(telemonitoring)) OR AB=(teleconference)) OR AB=(app)) OR AB=(apps)) OR AB=(mobile)) OR AB=("mobile health")) OR AB=(smartphone)) OR AB=(phone)) OR AB=(telephone)) OR AB=(cellphone)) OR AB=("mobile app")) OR AB=("cellphone app")) OR AB=("smartphone app")) OR AB=("smart phone app")) OR AB=("portable app")) OR AB=(wearables)) OR AB=("text messag*")) OR AB=(SMS)) OR AB=(notification )) OR AB=(reminder)) OR AB=(online)) OR AB=(internet)) OR AB=(wireless)) OR AB=("online intervention")) OR AB=(software)) OR AB=("computer based")) OR AB=("computer assisted")) OR AB=("remote intervention")) OR AB=("remote consultation")) OR AB=("digital health")) OR AB=(mhealth)) OR AB=(m-health)) OR AB=(ehealth)) OR AB=(e-health)) OR AB=(emedicine)) OR AB=(webbased)) OR AB=("web based")) OR AB=(webpage)) OR AB=(website)) OR AB=(gamification)) OR AB=("game based")) OR AB=("virtual reality")) OR AB=(digital )) OR AB=("digital technology")) OR AB=(laptop)) OR AB=(computer)) OR AB=(microsoft)) OR AB=(MAC)) OR AB=(IPAD)) OR AB=(tablet)) OR AB=(virtual)) OR AB=(electronic)) OR AB=("electronic health") and Preprint Citation Index (Exclude – Database)  4: (((((((((((((((((((((((((((((((((((((((((((((((((((((((((((((((((((((((((((((((((((((((((((((((((((((((((((((((((((TI=("medication adherence")) OR AB=("medication adherence")) OR TI=("medication adherance")) OR AB=("medication adherance")) OR TI=("medication compliance" )) OR AB=("medication compliance" )) OR TI=("medication persistence")) OR AB=("medication persistence")) OR TI=("medication non adherence” )) OR AB=("medication non adherence” )) OR TI=("medication nonadherence" )) OR AB=("medication nonadherence" )) OR TI=("medication non adherance" )) OR AB=("medication non adherance" )) OR TI=("medication nonadherance" )) OR AB=("medication nonadherance" )) OR TI=("medication non compliance")) OR AB=("medication non compliance")) OR TI=("medication noncompliance" )) OR AB=("medication noncompliance" )) OR TI=("medication non persistence" )) OR AB=("medication non persistence" )) OR TI=("medication nonpersistence" )) OR AB=("medication nonpersistence" )) OR TI=("medication use")) OR AB=("medication use")) OR TI=("medication control" )) OR AB=("medication control" )) OR TI=("patient compliance")) OR AB=("patient compliance")) OR TI=(Nebulizer)) OR AB=(Nebulizer)) OR TI=("medicine adherence" )) OR AB=("medicine adherence" )) OR TI=("medicine adherance" )) OR AB=("medicine adherance" )) OR TI=("medicine compliance" )) OR AB=("medicine compliance" )) OR TI=("medicine persistence")) OR AB=("medicine persistence")) OR TI=("medicine non adherence" )) OR AB=("medicine non adherence" )) OR TI=("medicine nonadherence")) OR AB=("medicine nonadherence")) OR TI=("medicine non adherance" )) OR AB=("medicine non adherance" )) OR TI=("medicine nonadherance" )) OR AB=("medicine nonadherance" )) OR TI=("medicine non compliance" )) OR AB=("medicine non compliance" )) OR TI=("medicine noncompliance" )) OR AB=("medicine noncompliance" )) OR TI=("medicine non persistence" )) OR AB=("medicine non persistence" )) OR TI=("medicine nonpersistence" )) OR AB=("medicine nonpersistence" )) OR TI=("medicine use")) OR AB=("medicine use")) OR TI=("medicine control")) OR AB=("medicine control")) OR TI=("drug adherence" )) OR AB=("drug adherence" )) OR TI=("drug adherance" )) OR AB=("drug adherance" )) OR TI=("drug compliance" )) OR AB=("drug compliance" )) OR TI=("drug persistence" )) OR AB=("drug persistence" )) OR TI=("drug non adherence" )) OR AB=("drug non adherence" )) OR TI=("drug nonadherence" )) OR AB=("drug nonadherence" )) OR TI=("drug non adherance" )) OR AB=("drug non adherance" )) OR TI=("drug nonadherance" )) OR AB=("drug nonadherance" )) OR TI=("drug non compliance" )) OR AB=("drug non compliance" )) OR TI=("drug noncompliance" )) OR AB=("drug noncompliance" )) OR TI=("drug non persistence" )) OR AB=("drug non persistence" )) OR TI=("drug nonpersistence")) OR AB=("drug nonpersistence")) OR TI=("drug use")) OR AB=("drug use")) OR TI=("drug control" )) OR AB=("drug control" )) OR TI=("treatment adherence" )) OR AB=("treatment adherence" )) OR TI=("treatment adherance")) OR AB=("treatment adherance")) OR TI=("treatment compliance" )) OR AB=("treatment compliance" )) OR TI=("treatment persistence")) OR AB=("treatment persistence")) OR TI=(“treatment non adherence" )) OR AB=(“treatment non adherence" )) OR TI=("treatment nonadherence")) OR AB=("treatment nonadherence")) OR TI=("treatment non adherance" )) OR AB=("treatment non adherance" )) OR TI=("treatment nonadherance" )) OR AB=("treatment nonadherance" )) OR TI=("treatment non compliance")) OR AB=("treatment non compliance")) OR TI=("treatment noncompliance" )) OR AB=("treatment noncompliance" )) OR TI=("treatment non persistence" )) OR AB=("treatment non persistence" )) OR TI=("treatment nonpersistence")) OR AB=("treatment nonpersistence")) OR TI=("treatment use")) OR AB=("treatment use")) OR TI=("treatment control” )) OR AB=("treatment control” ) and Preprint Citation Index (Exclude – Database)  5: #1 AND #2 AND #3 AND #4 and Preprint Citation Index (Exclude – Database)    6: #1 AND #2 AND #3 AND #4 and Preprint Citation Index (Exclude – Database)    7: (#1 AND #2 AND #3 AND #4) NOT (SILOID==("PPRN")) |
|  |  |

*Note. Search strategies combined controlled vocabulary and free-text terms for population, asthma, digital interventions, and medication adherence using Boolean operators. Syntax and field tags were adapted per database; preprints were excluded where applicable.*

*Symbols & abbreviations: # = number, AB = Abstract, KW = keyword, MeSH = Medical Subject Headings, MA = Major Descriptor, MJ = major topics, m_titl. = mapped title field, PPRN = Preprint Record Number, S = Search, SILOID = Silo Identifier, TI = Title, TITLE-ABS-KEY = Title, Abstract, and/or Keyword.*
